# *Operando* and *Postmortem* Study of Iridium-Decorated Titanium-Based Catalysts in PEM Water Electrolysis

**DOI:** 10.1021/jacs.6c04230

**Published:** 2026-09-23

**Authors:** Lucinda Blanco-Redondo, Yevheniia Lobko, Marco Bogar, Yurii Yakovlev, Jaroslava Nováková, Simone Pollastri, Alina Madalina Darabut, Miquel Gamón Rodríguez, Giovanna Marussi, Matteo Crosera, Michal Mazur, Milan Dopita, Viacheslav Kalinovych, Roberto Biagi, Heinz Amenitsch, Rodolfo Taccani, Iva Matolínová

**Affiliations:** † Department of Surface and Plasma Science, Faculty of Mathematics and Physics, 563251 Charles University, V Holešovičkách 2, Prague 180 00, Czech Republic; ‡ Department of Engineering and Architecture, 9315 University of Trieste, Via Alfonso Valerio 6/1, Trieste 34127, Italy; § Department of Physics, Computer Science and Mathematics, 9306 University of Modena and Reggio Emilia, Modena 41125, Italy; ∥ 27253 ELETTRA - Sincrotrone Trieste S.C.p.A., SS 14 - km 163,5, Basovizza, Trieste 34149, Italy; ⊥ Department of Chemical and Pharmaceutical Sciences, 9315 University of Trieste, Via Giorgeri 1, Trieste 34127, Italy; # Department of Physical and Macromolecular Chemistry, Faculty of Science, 37740 Charles University, Hlavova 8, Prague 128 00, Czech Republic; ∇ Department of Condensed Matter Physics, Faculty of Mathematics and Physics, 138735 Charles University, Ke Karlovu 5, Prague 121 16, Czech Republic; ○ Istituto Nanoscienze (NANO-S3), Consiglio Nazionale delle Ricerche (CNR), via G. Campi 213/a, Modena 41125, Italy; ◆ Centro H2-MORE, University of Modena and Reggio Emilia, Modena 41125, Italy; ¶ Graz University of Technology, Institute of Inorganic Chemistry, Stremayrgasse 9, Graz 8010, Austria

## Abstract

Understanding the dynamic degradation of oxygen-evolution electrocatalysts under realistic operating conditions is essential for advancing the performance and efficiency of proton exchange membrane (PEM) water electrolyzers. By combining *operando* Small-Angle and Wide-Angle X-ray Scattering (SAXS/WAXS) with electrochemical measurements, the evolution of iridium nanoparticles supported on TiO_2_, TiC, and TiN, loaded on full membrane electrode assemblies, and undergoing accelerated stress testing in these assemblies has been monitored in real time. SAXS analysis shows pronounced Ostwald ripening of Ir on TiO_2_, resulting in particle growth from 1.5 to 2.2 nm and a corresponding loss of electrochemically active surface area. In contrast, Ir supported on TiC remains morphologically stable. However, the TiC support undergoes severe degradation, losing approximately 80% crystallinity and releasing Ti species that migrate across the membrane. TiN exhibits an intermediate behavior, showing partial oxidation, moderate Ir growth, and limited Ti migration. *Postmortem* transmission electron microscopy, energy-dispersive X-ray spectroscopy, X-ray photoelectron spectroscopy, and X-ray absorption spectroscopy corroborate results from *operando* analyses. Results confirm the dissolution and oxidation of Ti in TiC and TiN, as well as the structural stability of TiO_2_, and better describe the boundaries of the trade-off between nanoparticle stability and support robustness. Moreover, results underscore the need to design catalyst–support systems capable of simultaneously suppressing Ir ripening and mitigating support corrosion. Finally, this study demonstrates the capabilities of *operando* SAXS/WAXS analysis from full membrane electrode assemblies to elucidate degradation pathways and inform the development of durable, next-generation anode catalysts for PEM electrolyzers.

## Graphical abstract

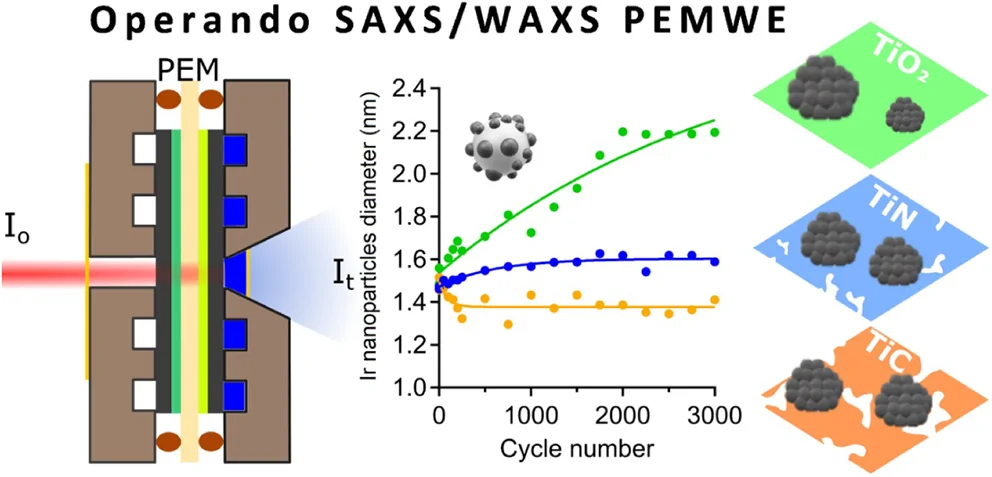

## 1 Introduction

The global transition toward sustainable and carbon-free energy systems positioned hydrogen as a key energy carrier for achieving climate-neutral targets.^1^ Proton Exchange Membrane Water Electrolyzers (PEMWEs) are among the most promising technologies for producing green hydrogen, due to their fast dynamic response and their capability to handle high current densities, which makes them suitable for operating with intermittent renewable power sources.^2–4^ However, the large-scale implementation of PEMWEs remains constrained by the high cost and limited durability of the materials involved in the anodic Oxygen Evolution Reaction (OER). To date, iridium-based materials constitute the benchmark catalysts owing to their superior OER activity and corrosion resistance in acidic environments.^5,6^ Nevertheless, the scarcity and high price of Ir make it essential to both improve its utilization and to extend catalyst lifetime; in these regards, different strategies are currently under study to improve catalyst performances, such as enhancing Ir dispersion,^7,8^ strengthening metal–support interactions,^9–11^ and reducing Ir loadings without sacrificing performance.^12,13^

Titanium-based materials have emerged as promising catalyst supports due to their favorable combination of chemical stability, electronic conductivity, and mechanical robustness.^14–20^ Despite these advantages, both Ir nanoparticles and Ti-based supports are susceptible to degradation under dynamic electrochemical and oxidative conditions typical of PEMWE operation. While Ir dissolution and nanoparticle agglomeration are recognized as major degradation pathways,^21–26^ the role of the support, its structural integrity, and its interaction with Ir remain not well-understood. Degradation of the support can lead to Ti dissolution, membrane contamination, and a loss of catalytic performance. Understanding the phenomena involved in the mutual degradation of Ir and support materials is thus critical for promoting the most rational design of durable and efficient OER catalysts.

In this framework, most studies have been conducted in three-electrode cells by using thin-film electrodes and liquid electrolytes. Nonetheless, results obtained using such experimental setups differ substantially from those in real membrane electrode assemblies (MEAs) and cannot fully represent the complex environment of operating PEMWEs. To overcome the limitations of *ex situ* characterization techniques in capturing catalyst degradation dynamics, several investigation techniques have been adapted to probe structural and chemical changes over time in *operando* or *in situ* conditions: for example, *operando* X-ray absorption spectroscopy (XAS),^7,16,27,28,30^ small-angle X-ray scattering (SAXS),^29^ and total scattering have elucidated the oxidation states of Ir, particle growth, and lattice restructuring during electrochemical cycling, while near-ambient pressure X-ray photoelectron spectroscopy (NAP-XPS) has been applied to track surface oxidation under controlled potentials.^30–32^

More recently, a limited number of studies started addressing complete MEAs for *operando* analysis via two-electrode cells, which would provide a more accurate representation of actual fuel-cell or electrolyzer operation. For instance, combined SAXS/XAS and quick-scanning X-ray absorption fine structure measurements have been used to monitor structural changes in Pt- and/or Pd-based catalysts within working MEAs.^33–39^ *Operando* XPS measurements have also demonstrated the evolution of Ir oxidation states under load.^40^ These studies highlight the importance and challenges of integrating advanced X-ray-based investigation techniques for studying full MEAs to capture the true degradation mechanisms of electrocatalysts under realistic conditions.

In this work, *operando* small- and wide-angle X-ray scattering (SAXS/WAXS) is employed to monitor the real-time structural evolution of Ir nanoparticles supported on TiO_2_, TiC, and TiN in MEAs undergoing accelerated stress testing (AST) in a full PEMWE. Complementary *postmortem* analyses, including transmission electron microscopy (TEM), energy-dispersive X-ray spectroscopy (EDX), XPS, and XAS, are used to correlate nanoscale morphological changes with chemical and structural transformations of the supports. By combining *operando* and *postmortem* characterization, this study elucidates the interplay between Ir nanoparticle evolution, support degradation, and overall OER performance. The results provide new mechanistic insights into the degradation pathways of Ti-supported Ir catalysts, guiding the development of durable, resource-efficient anode materials for next-generation PEM water electrolyzers.

## 2 Experimental Part

### 2.1 MEA Preparation and Electrochemical Cell Setup

Catalyst Coated Membranes (CCMs) with an active area of 1 cm^2^ (1 × 1 cm^2^) were fabricated in-house: for the cathode, a Pt/C catalyst layer (0.05 mg_Pt_·cm^–2^) was employed, while the anode contained Ir/TiO_2_, Ir/TiC, or Ir/TiN catalysts synthesized as previously described.^41^ The cathode layer was deposited on an NR212 membrane (50.8 μm; FuelCell Store) by ultrasonic spraying (ExactaCoat, SonoTek). The ink consisted of Pt/C (40 wt % on Vulcan XC-72R), a 1:1 isopropanol/acetone solvent mixture, and D521 Nafion ionomer (5 wt %), formulated to achieve a Pt loading of 0.05 mg·cm^–2^ with an ionomer/carbon ratio of 0.6.^42^ The anode catalyst ink was applied onto the membrane using a Meyer-rod technique (120 μm wire, Erichsen). The ink was prepared by ultrasonic mixing of 70 mg of catalyst powder with D521 ionomer, followed by five sequential depositions to achieve an Ir loading of 1 mg_Ir_·cm^–2^. Carbon fiber paper (Sigracet 29BC, FuelCellStore) was used as the cathode GDL, while a platinated titanium 0.005” mesh (FuelCell Store) served as the anode porous transport layer.

The MEAs were then secured within an electrochemical cell properly designed to allow X-ray transmission across the MEA without affecting the operative conditions of the electrolyzer (Figure S1):^37^ a slit was properly made in the flow field of Ti bipolar plates (0.8 mm deep flow field and a central 0.8 × 1 mm X-ray window), beveled to guarantee an exit angle for the diffracted patterns up to 45° from the beam axis; X-ray windows were sealed with 13 μm Kapton films (DuPont). To ensure that the SAXS signal originates predominantly from the Ir nanoparticles, the Pt loading on the cathode was kept intentionally low to minimize the SAXS signal of Pt nanoparticles at the cathode, which have a particle size comparable to that of the synthesized Ir nanoparticles (∼2 nm).

### 2.2 Accelerated Stress Test Protocol

The Accelerated Stress Test (AST) consisted of square-wave potential cycling between 0 and 2.0 V, with a 5 s hold at each potential, repeated for a total of 3000 cycles. The upper potential limit reflects limiting working PEMWE anode conditions where Ir- and Ti-based supports undergo accelerated oxidation and dissolution.^21,43^ This potential limit was selected to ensure detectable morphology evolution, as milder conditions are expected to induce only minor morphological changes within the time frame of the experiment. SAXS and WAXS diffraction patterns were collected every 25 cycles, whereas Cyclic Voltammetry (CV, potential range 0–2 V, sweep rate: 20 mV·s^–1^) and Potentiostatic Electrochemical Impedance Spectroscopy (PEIS) measurements (DC bias of 1.5 V, frequency range 100 kHz–1 Hz) were performed at 250, 1000, 1750, 2250, and 3000 cycles. Before initiating the AST, the MEA was conditioned at 70 °C under a flow rate of 100 mL·min^–1^ of deionized Milli-Q water to ensure full hydration. Each MEA was also examined in its pristine state prior to electrochemical operation. For this purpose, *ex situ* SAXS and WAXS measurements were carried out immediately after assembling the CCM in the electrolysis cell, under ambient temperature and pressure, to capture the initial nanoparticle morphology before hydration or potential cycling. PEIS data were analyzed using the Distribution of Relaxation Times (DRT) analysis.^44,45^ Reproducibility of the electrochemical results was verified by the laboratory measurements, and the corresponding results are provided in Sections S9 and S17 of the Supporting Information (SI).

### 2.3 Operando SAXS and WAXS

SAXS spectra were collected in transmission on the Austrian SAXS beamline at the ELETTRA synchrotron in Trieste, Italy.^46^ Measurements were performed at room temperature and pressure, at a wavelength of 0.77 Å (16 keV). For SAXS measurements, the sample-to-detector distance was set to 2028 mm, silver behenate was used as a calibrant, and a 2D pixel detector (Pilatus3 1M, Dectris) was used to record scattering patterns, which were radially integrated via the SAXSDOG software,^47^ providing a 1D pattern ranging from 0.14 nm^–1^ to 7.06 nm^–1^.

WAXS patterns were collected at a sample-to-detector distance of 333 mm. Both para-bromobenzoic acid and lanthanum hexaboride (LaB_6_) served as calibrants. A 2D pixel detector (Pilatus 100K, Dectris) was used to record diffraction patterns within an angular range of 21.5 to 52 degrees.

IGOR Pro software (IGOR Pro 7.0.8.1, WaveMetrics) was used for data reduction and fitting. After background subtraction, SAXS patterns undergo least-squares fitting using a model built following a step-by-step approach.^38^ The detailed procedure for SAXS and WAXS data analysis is described in the Supporting Information (SI), in Sections S3 (SAXS data analysis) and S4 (WAXS data analysis).

### 2.4 Postmortem Characterization

After completion of the AST, the catalyst layers were analyzed by using TEM, SEM/EDX (both planar and cross-sectional), ICP-OES, XPS, and XAS to evaluate structural, chemical, and elemental changes.

TEM analysis was performed using high-resolution transmission electron microscopy (HR-TEM) on a JEOL 2200FS and a JEOL NEOARM-200F microscope, both operating at 200 kV. SEM/EDX analyses were performed at 20 kV by using a Tescan MIRA 3 microscope equipped with a Bruker Quantax XFlash 6-10 EDX detector. Pieces of the MEA were mounted on carbon double-sided tape for planar-view imaging, while cross-sections were prepared by fixing the MEA vertically in a dedicated holder.

Process water, sampled from the anode circuit after the AST, was analyzed by Inductively Coupled Plasma-Optical Emission Spectroscopy (ICP-OES) to quantify the concentrations of Ir, Ti, and S ions. Measurements were performed with an Optima 8000 spectrometer (PerkinElmer, USA) equipped with an S10 Autosampler. Instrument calibration was performed using a set of standard solutions (0.01 to 10 mg·L^–1^), prepared by serial dilution from 10 mg·L^–1^ multielement ICP standards (Periodic Table MIX 1 and MIX 2, TraceCERT, Sigma-Aldrich). Quantification of analytes was based on a linear calibration curve within this concentration range (R^2^ ≥ 0.999). Analytical accuracy was assessed with two independent quality-control solutions (0.1 μg·L^–1^ and 1 mg·L^–1^), prepared from a multielement standard not used for calibration (Multielement Quality Control Standard for ICP, VWR Chemicals). The limits of detection for each analyte were as follows: Ir (215.268 nm) 0.02 mg·L^–1^; Ti (334.940 nm) 0.1 mg·L^–1^; S (181.975 nm) 0.5 mg·L^–1^. Measurement precision, expressed as repeatability (RSD%), was <5%.

XPS measurements were performed using an EnviroESCA system (SPECS GmbH) equipped with a monochromatic Al Kα source (1486.71 eV) and a Phoibos 150 NAP 1D-DLD64 analyzer. MEA specimens were mounted on stainless-steel sample holders using carbon double-sided tape. Measurements were acquired in fixed analyzer transmission mode at 10^–7^ mbar with a 200 μm beam spot. Core-level spectra (Ir 4f, Ti 2p, C 1s, N 1s, O 1s, F 1s) were recorded with a 0.1 eV step size, 0.3 s dwell time, and 20 eV pass energy. Data processing was performed using KolXPD.^48^ All spectra were fitted as recorded using a Shirley background. The Ir 4f signal was deconvoluted into multiple doublets using an asymmetric Doniach–Šunjić function, with the 4f^7/2^–4f^5/2^ branching ratio fixed at 4:3 and a 3 eV splitting. The Ti 2p signal was fitted using Voigt doublets with spin–orbit splits of 5.7 eV (Ti–O) and 6.0 eV (Ti–C/Ti–N/Ti–O_*x*_N_*y*_); the Ti 2p^1/2^ amplitude was set to half that of Ti 2p^3/2^, with a doubled Gaussian width for the 2p^1/2^ component. Cross-section-corrected peak areas (0.107 for Ti 2p and 0.204 for Ir 4f^48^) were used to determine relative elemental ratios.

XAS measurements were performed at the XAFS beamline^49^ of the ELETTRA synchrotron (Trieste, Italy). Ti K-edge and Ir L_3_-edge XANES spectra were collected in transmission mode using ionization chambers as detectors and a fixed-exit double-crystal Si(111) monochromator. For all absorption edges, the energy was calibrated by simultaneously collecting a spectrum from a metal reference placed in a second experimental chamber downstream of both the sample and the I1 ionization chamber. The positions of the first inflection points were taken at 4966 and 11215 eV for Ti and Ir, respectively. All spectra were collected with a variable energy step as a function of the energy: large step (5 eV) in the first 200 eV of the spectrum, smaller step (0.2 eV) in the XANES region, and a k-constant step of 0.03 Å^–1^ in the high-energy region. In all acquired spectra, the commonly used normalization step of setting the absorbance to unity has been performed using Athena software.^50,51^ The normalized XANES spectra were compared with the spectra of model compounds from the beamline database, which were also used to perform linear combination fitting analyses using Athena software.

## 3 Results and Discussion

To investigate the degradation behavior of Ir-based anode catalysts under PEMWE operation under ASTs, *operando* SAXS and WAXS analyses were supported by *postmortem* XPS, XAS, EDX, and detailed electrochemical characterization. The following section presents the electrochemical response of the Ir/TiO_2_, Ir/TiC, and Ir/TiN MEAs during the AST and correlates these trends with catalyst activation and degradation processes.

### 3.1 Electrochemical Analysis

Figure 1 shows the evolution of the electrochemical behavior of the three MEAs during the AST, including cyclic voltammetry (CV) (Figure 1a–f), current density at 0 and 2 V (Figure 1g–i), the cathodic peak from CV at 1.250 V (Figure 1j), and current density at 1.525 V (Figure 1k). Specific outcomes from each technique are discussed in detail in the next sections.

**Figure 1:**
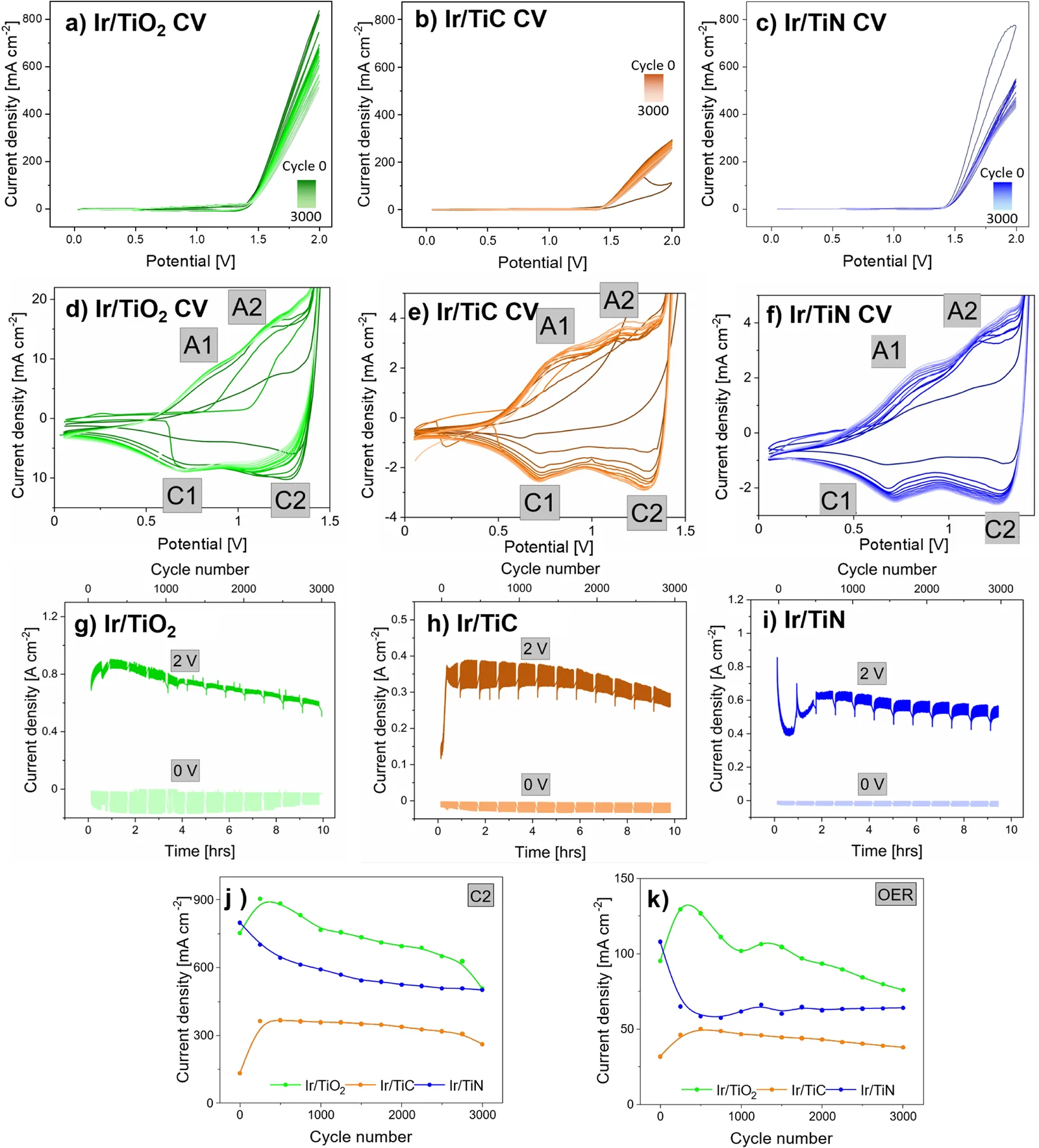
Time-resolved evolution of (a–c) cyclic voltammograms, (d–f) magnified views of the cyclic voltammograms shown in (a–c), (g–i) current density profiles recorded during the 0 and 2 V holds, (j) cathodic peak C2 current density at 1.250 V, and (k) current density at 1.525 V for Ir/TiO_2_, Ir/TiC, and Ir/TiN MEAs during the *operando* measurements. Active area, 1 cm^2^.

#### 3.1.1 Activation Behavior and Ir Redox Features

In the low-potential region (0.06–0.4 V) (Figure 1d–f), the specific hydrogen adsorption/desorption features, which are characteristic of metallic Ir, are absent in all samples, indicating that the Ir surface is fully oxidized after the conditioning step. At higher potentials (≥0.5 V), the CVs (Figure 1d–f) exhibit both the anodic peaks (A1, A2, associated with Ir oxidation) and cathodic ones (C1, C2, associated with Ir species reduction), consistent with typical Ir oxide electrochemistry.^21^

The evolution of these redox features differs significantly across the three catalysts. Within the initial 250 cycles, both features A2 and C2 (Figure 1d–f and j and Figure S2) increase for all MEAs, except for the A2 peak in Ir/TiC, indicating the progressive activation and equilibration of the catalyst layers.^22^ This initial rise in peak intensity reflects the end of the MEA’s conditioning toward a state of steady operational behavior.

#### 3.1.2 Electrochemical Stability during AST Cycling

After the initial 250 cycles (Figure 1g–i and k, Figure S2a), the three catalysts exhibit distinct stability trends under AST: Ir/TiO_2_ (Figure 1d,j, Figure S2a) shows a continuous decline in both A2 and C2 peak charges, with the feature C2 decreasing by nearly 50% over the full test. The loss of Ir redox charge may be associated with Ir dissolution, particle growth, surface restructuring, or interfacial changes that reduce electrochemical activity.^16,52^ These possible degradation pathways are discussed in detail below.

In contrast, Ir/TiC and Ir/TiN maintain or even increase their redox peak charges during the same time. Ir/TiC (Figure 1e,j, Figure S2a) shows a steady increase in both A2 and C2 peaks, whereas Ir/TiN (Figure 1f,j, Figure S2a) shows a pronounced increase in C2 and a more moderate increase in A2. These trends suggest improved redox reversibility and retention of electrochemically active Ir sites, reflecting enhanced structural stability compared to the oxide-supported catalyst. Overall, the TiC and TiN supports sustain Ir stability under aggressive cycling, whereas Ir/TiO_2_ undergoes a significant loss of Ir active surface area yet shows the highest electrochemically accessible active surface area (Figure 1a,d,g, Figure S2b). These variations highlight the strong influence of the support on the durability of the PEMWE anode catalysts.

#### 3.1.3 Evolution of Electrochemical Activity in the OER Region

The evolution of the current density in the OER region during the AST (Figure 1) shows clear differences among the Ir/TiO_2_, Ir/TiC, and Ir/TiN catalysts. Ir/TiO_2_ displays a brief activation during the first 250 cycles, followed by a pronounced and continuous decline in performance in the OER region (Figure 1k), indicating significant degradation under repeated voltage cycling. In contrast, Ir/TiC exhibits slower activation and a more moderate loss of activity (Figure 1k), while Ir/TiN shows an initial drop, followed by stable operation over the remaining cycles (Figure 1k). Overall, the absolute activity in the OER region is higher in Ir/TiO_2_, with respect to Ir/TiN, while Ir/TiC shows the poorest performance. However, Ir/TiC and Ir/TiN retain a larger fraction of their initial activity during the extended cycling. These differences underscore the strong role of the support on Ir stability and point to enhanced electrochemical activity retention for the nitride and carbide systems relative to the Ir/TiO_2_.

### 3.2 Morphological Analysis

To link electrochemical evolution with morphological changes in the MEAs, time-resolved scattering patterns were collected in operando conditions every 25 CV cycles. Results are shown in Figure 2, where the evolution of the scattering patterns within the three formulations is compared. The recorded scattering patterns are composed by three main features: a bump in the high-q region (around 3 nm^–1^), produced by the Ir NPs; a peak present around 1 nm^–1^, which corresponds to the so-called ionomer peak of Nafion; and a faint shoulder which can be spotted in the low-q regime, whose presence can be related to the supporting nanoparticles, as detailed in the Supporting Information in Section S3. By comparing pattern evolution over cycling, the most noticeable changes regard the shift of the ionomer peak toward lower-q values, which is an indicator of ongoing hydration of Nafion also during the ASTs.^53–56^ Regarding the catalyst layers, the morphological evolution of Ir nanoparticles in the MEA loaded with Ir/TiO_2_ is characterized by the progressive reduction of the intensity of the feature generated by Ir NPs (in the high-q region), while for Ir/TiC and Ir/TiN, the evolution of Ir NPs is less pronounced. Scattering pattern evolution is slightly different for the three supports too (low-q region), where no remarkable changes can be noticed for both Ir/TiO_2_ and Ir/TiN catalysts, while a loss of intensity can be observed for Ir/TiC only.

**Figure 2:**
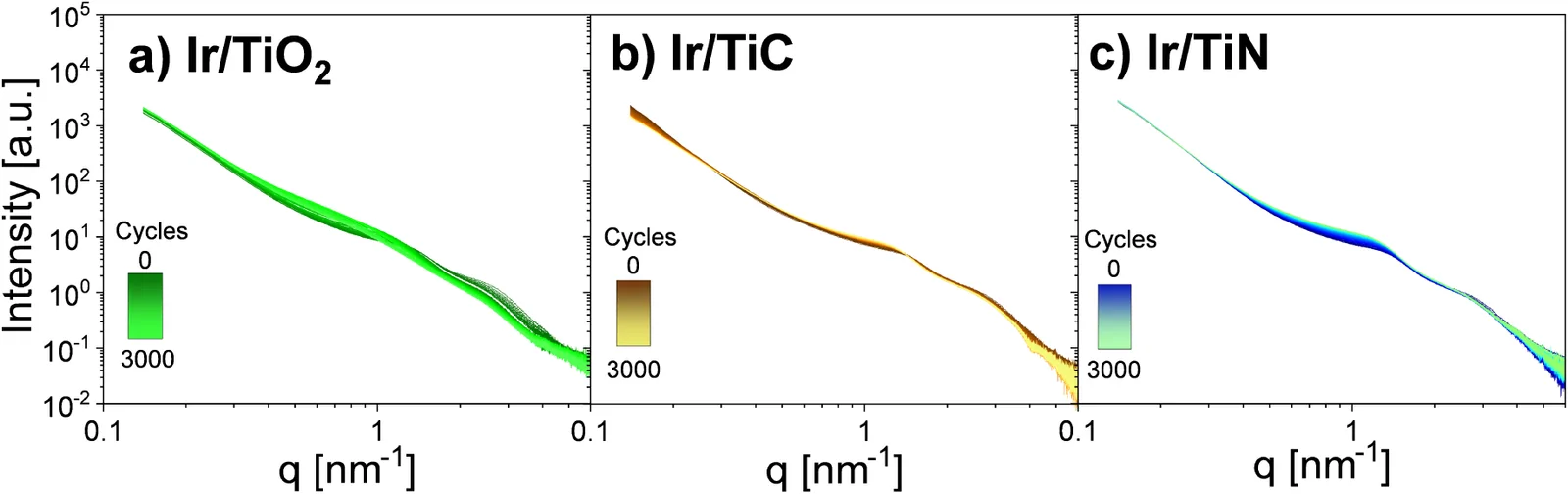
Time-resolved evolution of SAXS patterns recorded from MEAs loaded with (a) Ir/TiO_2_, (b) Ir/TiC, and (c) Ir/TiN catalysts while applying AST in *operando* conditions.

To obtain quantitative information, SAXS patterns were fitted with the analytical model presented in Supporting Information, developed to describe the morphological evolution involving both the support and the catalyst particle populations by using two sets of nanoparticles following the Schulz distribution.^57,58^ Complete fit results for the three samples are shown in Figures S7–S9, while the evolution of the most important parameters for the three formulations is summarized in Figure 3.

**Figure 3:**
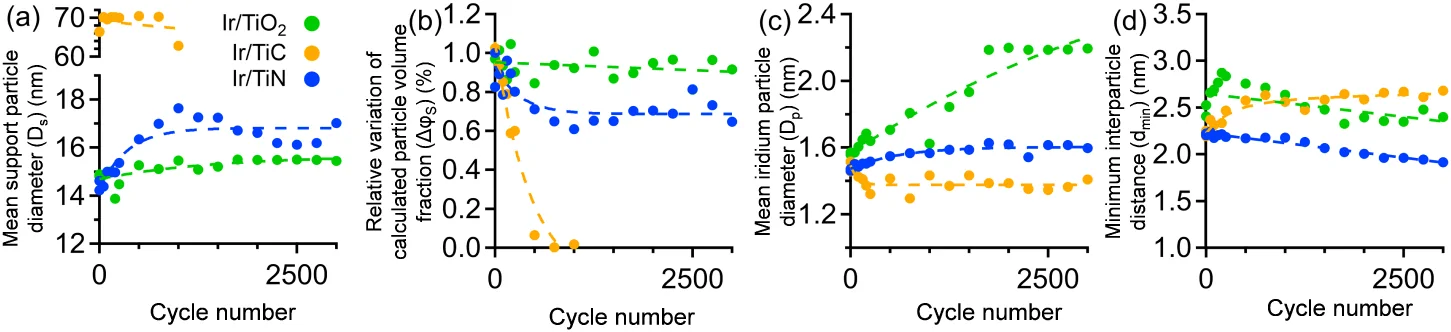
Time-resolved evolution of the main parameters used to fit the scattering patterns of Figure 2 with the analytical model detailed in Supporting Information. Support-related parameters: (a) mean particle diameter (D_S_) and (b) relative variation of the calculated particle volume fraction (Δφ_S_). Catalyst-related parameters: (c) mean particle diameter (D_p_) and (d) minimum interparticle distance (*d*_min_). *Note:* dashed lines are a guide for the eyes only.

For TiO_2_, both mean support particle size (D_S_, Figure 3a) and the distribution width (σ_S_, Figure S7c) remain unchanged under ASTs. Consequently, the calculated support particle volume fraction (which can be related to the amount of matter participating in scattering) shows no significant variation over time (Δφ_S_, Figure 3b, expressed as a relative variation from the initial value). For TiN, a slight increase in particle size (Figure 3a) and distribution width (Figure S9c) is noticeable within the first 1000 cycles; consequently, over the same time frame, a reduction of the particle volume fraction can be revealed. In contrast, TiC exhibits the most pronounced degradation after about 1000 cycles: an initial decline in particle size is followed by an abrupt drop of its particle volume fraction (Figure 3b) to zero. At this point, the contribution from the TiC support is not detectable anymore, becoming indistinguishable from the background due to the presence of the cathodic gas diffusion layer, whose contribution is described by the power law term included in the model. In these regards, the results are consistent with the fact that the WE remains operational and Ir nanoparticles are still detectable, thus, the loss of forward scattering probability from the TiC support is consistent with severe support degradation and pronounced dissolution, which is expected to dissolve the structures detectable within the probed q range.

Regarding catalyst nanoparticles, the mean particle diameter (D_P_, Figure 3c) for the MEA loaded with Ir/TiO_2_ exhibits a progressive growing trend over cycling. Starting from an initial value of 1.49 ± 0.02 nm (cycle 0), the mean particle size increases to 1.72 ± 0.02 nm after 1000 cycles and reaches a maximum value of 2.19 ± 0.05 nm at 2000 cycles. As previously reported by some of the present authors,^59^ and further supported by the theoretical and experimental studies of Kregar and coworkers,^60,61^ the evolution of the particle size distribution and the corresponding skewness values (Figure S10)^62^ suggest Ostwald ripening is the dominant mechanism leading to Ir NP growth and a reduction in the electrochemically active surface area, in agreement with the observed loss of performance and redox charge shown in the present study. For Ir/TiO_2_, after an initial increase, *d*_min_ gradually decreases from 2.46 ± 0.01 nm at cycle 0 to 2.39 ± 0.02 nm after 2000 cycles. This trend is consistent with the other parameters describing particle size evolution, with the slight increase in the apparent volume fraction (φ_SHS_, Figure S7h), and the behavior observed in cyclic voltammetry. However, contributions from other degradation mechanisms, including particle coalescence and dissolution/redeposition, cannot be excluded. The detection of dissolved Ir species in the electrolyte (Table S6) and postmortem HR-TEM observations (Figure 4 and Figure S30) indicate that multiple degradation pathways may occur simultaneously.

**Figure 4:**
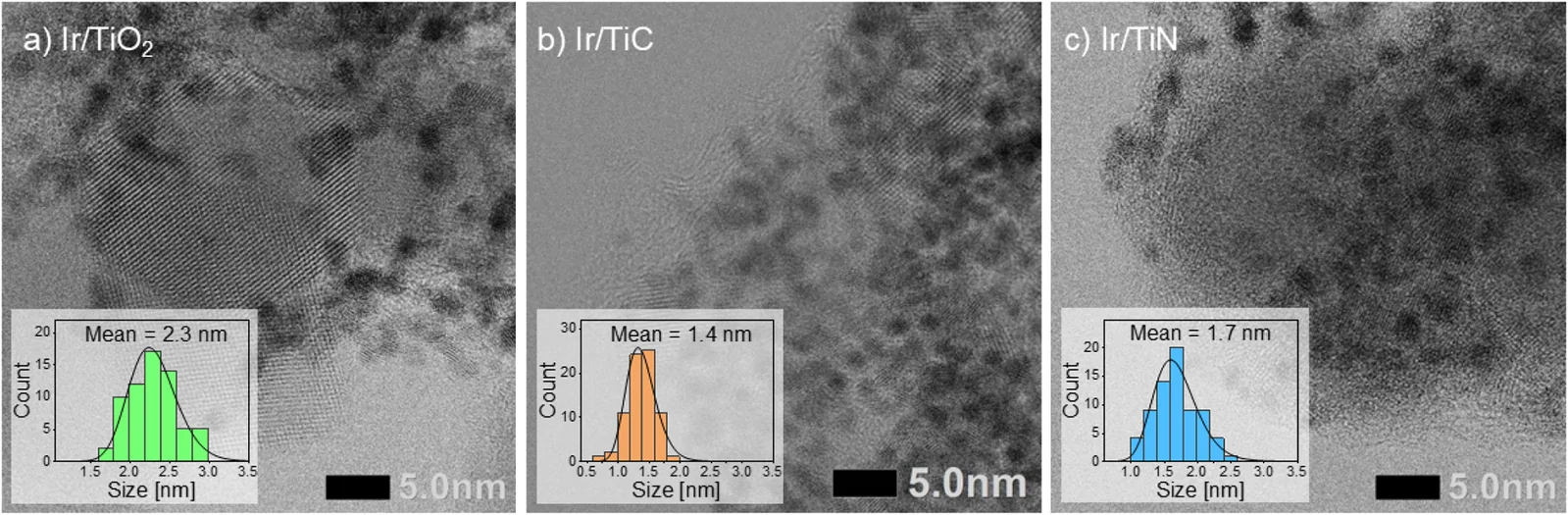
HR-TEM micrographs of nanoparticles from the anode of (a) Ir/TiO_2_, (b) Ir/TiC, and (c) Ir/TiN MEAs after *operando* experiments and Ir particle size distribution (insets).

Conversely, in the MEA loaded with Ir/TiC, the particle size evolution remains constant at 1.45 ± 0.02 nm over the whole stress test, and a possible explanation will be discussed later in the manuscript. The structure factor parameters slightly vary during the first 1000 cycles (*d*_min_ grows from 2.19 ± 0.01 nm to 2.58 ± 0.01 nm, Figure 3d, φ_SHS_ slightly decreases, Figure S8h) and then remain essentially constant until the end of the AST. This behavior suggests that the NP distribution remains stable, consistent with the evolution of the parameters describing the particle form factor.

An intermediate trend is observed for Ir nanoparticles in Ir/TiN. Here, the mean particle size remains nearly constant (1.45–1.50 ± 0.01 nm) during the first 1000 cycles, increasing to approximately 1.60 ± 0.01 nm between 1000 and 2000 cycles and remaining stable thereafter until the end of the stress test. From the structure factor parameters, distinct trends in the evolution of the minimum interparticle distance (*d*_min_, Figure 3d) were observed for each MEA. Finally, Ir/TiN shows a slight decrease in minimum interparticle distance, in agreement with the trend observed for Ir/TiO_2_.

### 3.3 Structural Analysis

Results from *operando* WAXS are presented in Figures S11–S13. In Figure S11, peak indexing is compared for the three catalyst formulations. In Figures S12 and S13, WAXS patterns recorded under *operando* conditions at initial (cycle 0) and final conditions (cycle 3000) from the MEAs loaded with Ir/TiO_2_ (Figure S12a and Figure S13a–e), Ir/TiC (Figure S12b and Figure S13f–j), and Ir/TiN (Figure S12c and Figure S13k–o) are compared. While no features originating from Ir nanoparticles are detectable due to the strong diffraction peaks of the hydrated Nafion (Figure S11), diffraction peaks from TiO_2_, TiC, and TiN supports could be indexed.

Peaks assigned to the rutile TiO_2_(110) and anatase TiO_2_(101) planes show no remarkable variations in peak area (Figure S13b), position (Figure S13c), or width (Figure S13d), evidencing good crystalline stability for this support. In contrast, the intensities of the TiC(200) and TiC(111) peaks decrease markedly in the diffraction patterns (Figure S12b and Figure S13f), with the corresponding peak areas (Figure S13g) decreasing by 90.4% and 69.4%, respectively. While this reduction could be due to phase amorphization, on the basis of the previous results, the disappearance of TiC diffraction peaks is expected to be primarily attributed to the dissolution of TiC nanoparticles. This effect is particularly pronounced for the TiC(111) reflection, where the substantial decrease in both crystallite size (Figure S13j) and peak area (Figure S13g) provides strong evidence for progressive dissolution and loss of TiC material. In contrast to the *postmortem* analysis of the MEA loaded with the Ir/TiC catalyst after *in situ* PEMWE testing, no diffraction peaks characteristic of anatase TiO_2_ are observed here.^41^ For Ir/TiN, the peak evolution of TiN(111) and TiN(200) planes is shown in Figure S12c and in Figure S13k–l. Again, an intermediate behavior is observed between TiO_2_ and TiC, with a 50% decrease in peak area between 2000 and 3000 cycles (Figure S13l), in agreement with the trend observed in the support volume fraction evolution highlighted by SAXS.

### 3.4 Physicochemical Analysis of the MEAs after Operando Testing

To complement the results from time-resolved *operando* measurements, *ex situ postmortem* characterization of the MEAs was performed to provide further insight into the structural and compositional changes that occurred after AST.

Figure 4 presents HR-TEM micrographs of the catalyst layers of the samples that had previously been stressed. Mean Ir particle sizes, determined from the TEM images, are consistent with the SAXS values (Figure 4a), yielding 2.3 nm ± 0.29 nm for Ir/TiO_2_, 1.4 ± 0.22 nm for Ir/TiC, and 1.7 ± 0.28 nm for Ir/TiN. Although TEM provides localized information, clear structural differences are observed among the supports. TiO_2_ nanoparticles preserve their crystalline structure, as evidenced by distinct lattice fringes and sharp particle edges (Figure 4a). In contrast, the Ir/TiC sample exhibits Ir nanoparticles dispersed over poorly defined, amorphous regions, consistent with the WAXS results (Figure S12b), indicating TiC degradation. For Ir/TiN, the absence of a discernible crystalline structure further supports the formation of an amorphous oxide layer, in agreement with previous observations.^41^ These *postmortem* TEM findings corroborate results obtained from *operando* SAXS and WAXS analyses (Figure S12), confirming that the structural instabilities observed for TiC and TiN under AST conditions result in permanent morphological degradation, whereas TiO_2_ retains its crystalline integrity throughout testing.

#### 3.4.1 Chemical State Analysis by XPS and XAS

To elucidate the chemical transformations occurring in the catalyst layer during the AST, *postmortem* XPS and XAS analyses were performed *ex situ* on the anode side. Figure 5 displays the Ti 2p and Ir 4f core-level spectra obtained by XPS, as well as corresponding Ti K-edge and Ir L_3_-edge XANES spectra.

**Figure 5:**
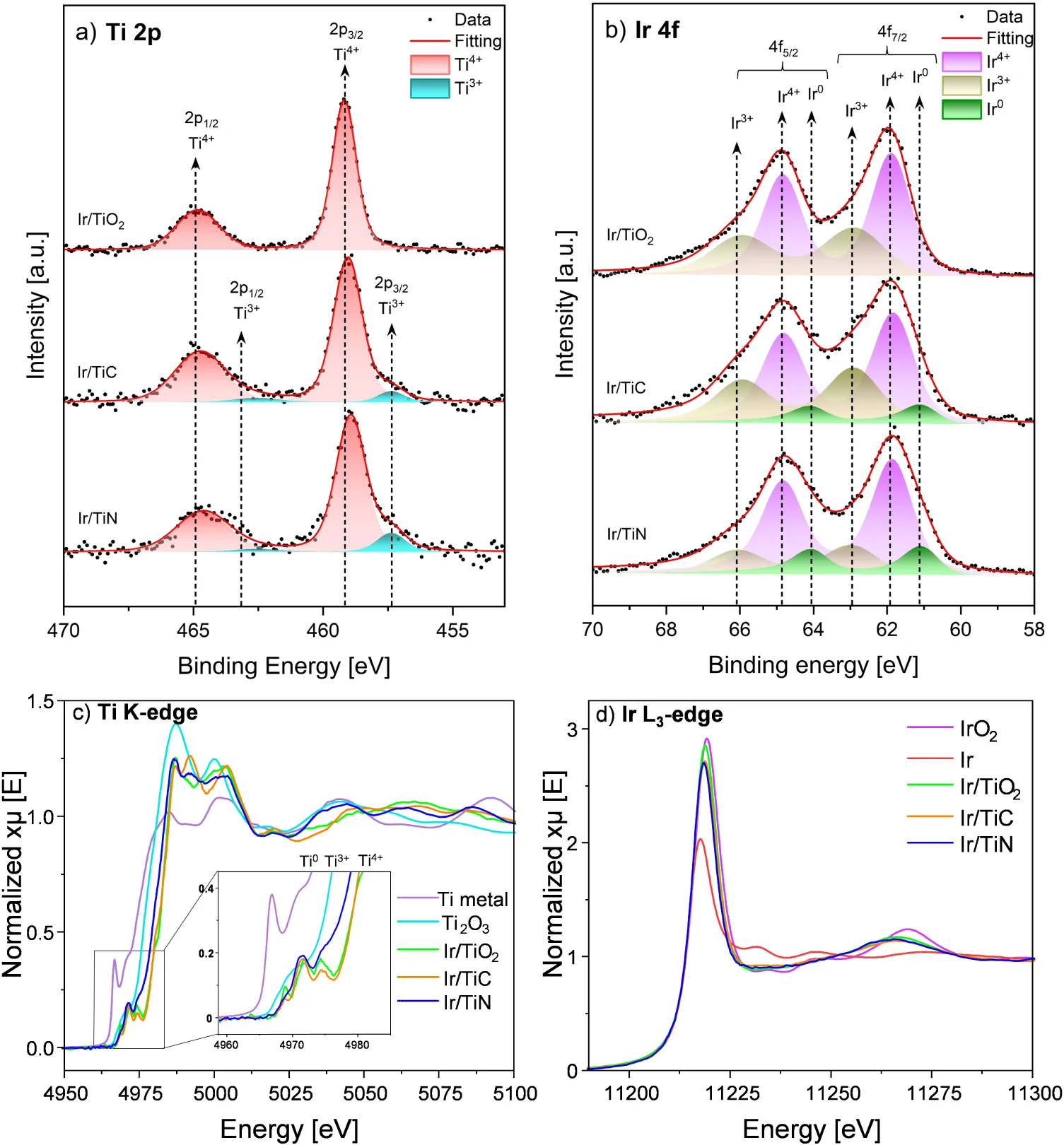
XPS and XAS characterization of the MEA anodes after *operando* tests. (a) Ti 2p core-level XPS spectra for the Ir/TiO_2_, Ir/TiC, and Ir/TiN anodes; (b) Ir 4f core-level XPS spectra of the same samples; (c) normalized Ti K-edge XANES spectra of the investigated catalysts compared with Ti_2_O_3_ and Ti metal foil references. The inset shows an enlarged view of the pre-edge region; (d) normalized Ir L_3_-edge XANES spectra of the catalysts together with IrO_2_ and Ir metal reference compounds.

XPS results reveal complete oxidation of the surface of the titanium-containing supports in all samples (Figure 5a). For Ir/TiO_2_, Ir/TiC, and Ir/TiN, the Ti 2p spectra exhibit a dominant doublet at 459.0 eV (Ti 2p^3/2^) and 464.7 eV (Ti 2p^1/2^), which is characteristic of Ti^4+^ species. In Ir/TiC and Ir/TiN, an additional shoulder at lower binding energies (Ti 2p^3/2^ at 457.5 eV and Ti 2p^1/2^ at 462.5 eV) corresponds to Ti^3+^ states.^63–65^ This feature suggests partial oxidation of TiC and TiN, forming substoichiometric TiO_*x*_ layers containing oxygen vacancies that can enhance electronic conductivity and Ir-support charge transfer. Such partial oxidation is consistent with the limited stability of TiC and TiN under acidic oxidative conditions, as demonstrated by the respective Pourbaix diagrams and corrosion studies.^43,62,66,67^

Complementary insights from the Ti K-edge XANES spectra (Figure 5c) confirm these observations. Among the three materials, Ir/TiN demonstrates the smallest edge shift, indicating partial oxidation to a mixed Ti^3+^/Ti^4+^ state, as corroborated by linear combination fitting analysis showing that 41% of Ti is present in Ti^3+^-containing phases, while the remaining 59% is associated with Ti^4+^-containing phases (Figure S14c, Table S4). In contrast, Ir/TiC displays a stronger shift, consistent with predominant oxidation to Ti^4+^. The Ti^4+^ species in Ir/TiC exhibit mainly rutile-like coordination, as evidenced by the characteristic feature at ∼4991 eV and an estimated 61% (±6) rutile fraction (Figure S14b, Table S4). The Ir/TiO_2_ catalyst retains its Ti^4+^ edge position, with XANES features primarily corresponding to anatase, which represents approximately 57% (±4) of the total Ti (Figure S14a, Table S4). The results are consistent with the XPS ones, confirming the structural stability of the TiO_2_ support during cycling. The low-intensity pre-edge peaks indicate that Ti atoms remain predominantly in the centrosymmetric 6-fold coordination, consistent with an octahedral environment, as reported in some relevant literature works on this topic.^68–70^

The Ir 4f XPS spectra (Figure 5b) reveal both Ir^4+^ (IrO_2_-like) and Ir^3+^ components, with their relative intensities varying with the support. For Ir/TiC and Ir/TiN, a clear doublet attributed to metallic Ir is also detected, whereas Ir/TiO_2_ shows only oxidized states. The presence of metallic Ir in Ir/TiC and Ir/TiN can be attributed to incomplete electrochemical activation resulting from concurrent support oxidation and electronic interactions between Ti^3+^ species and Ir oxides, which may induce partial reduction of Ir, as previously reported.^71,72^ These findings explain the lower electrochemical activity in the OER region of Ir/TiC and Ir/TiN compared with Ir/TiO_2_, as the presence of metallic Ir and partially degraded supports hinders charge transfer and catalyst utilization.

At the Ir L_3_-edge (Figure 5d), the XANES spectra of all samples closely resemble the IrO_2_ reference, despite a slightly lower intensity of the white line in samples Ir/TiC and Ir/TiN, which can be observed and is compatible with a small amount of reduced Ir in those samples. Overall, Ir remains predominantly in the Ir^4+^ oxidation state throughout the bulk, consistent with surface-sensitive XPS results.

Thus, combined XPS and XAS analyses demonstrate a clear correlation between support oxidation and electrochemical performance. TiC and TiN supports undergo partial oxidation to Ti^3+^/Ti^4+^ states, accompanied by Ti dissolution and partial Ir reduction, while TiO_2_ maintains its fully oxidized Ti^4+^ structure and stabilizes the IrO_2_ phase.

#### 3.4.2 Elemental Redistribution and Electrochemical Correlation (EDX and DRT Analyses)

To further elucidate the degradation mechanisms inferred from the SAXS/WAXS and XPS/XAS analyses, EDX mapping and DRT analyses were conducted to examine elemental migration and changes in charge-transport behavior after *operando* testing.

EDX analyses of both the anode and cathode (Figures S15–S17) reveal significant differences in Ti distribution among the MEAs. The Ir/TiO_2_ anode displays a uniform Ti signal confined to the anode side, with no detectable Ti on the cathode, confirming the structural stability of TiO_2_ during the AST. In contrast, the Ir/TiC and Ir/TiN samples exhibit distinct evidence of Ti migration. For Ir/TiC, Ti appears as a discontinuous, low-intensity layer on the anode, while a strong Ti signal is detected on the cathode side, forming a clear intensity gradient across the membrane (Figure 6f). A similar but less pronounced Ti ion migration is observed for Ir/TiN, where Ti ions are detected on the cathode, though with a higher residual concentration at the anode compared to Ir/TiC. These observations are corroborated by the elemental ratios extracted from EDX spectra (Figure S15): Ir/Ti ratios of 0.15 (Ir/TiO_2_), 0.32 (Ir/TiC), and 0.22 (Ir/TiN) on the anode and Pt/Ti ratios of 18.6, 0.07, and 6.6 on the cathode, respectively. The increased Ti content on the cathode side for Ir/TiC and Ir/TiN indicates dissolution and migration of Ti cations through the membrane during operation. Although the 0–2.0 V AST represents a highly accelerated degradation protocol, qualitatively identical support-dependent degradation trends were also observed under milder conditions, including a 0–1.7 V AST and 0.4 A cm^–2^ constant-current operation (Section S9), indicating that the observed behavior reflects intrinsic catalyst–support stability rather than an artifact of the applied stress window.

**Figure 6:**
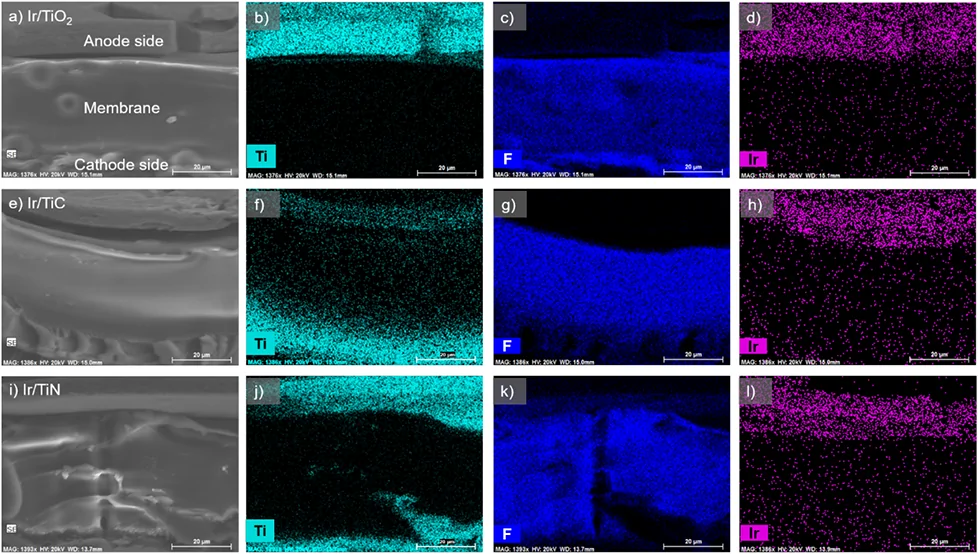
SEM images (a, e, and i) and EDX mapping (b–d, f–h, and j–l) of the MEA cross-section after *operando* experiments for Ir/TiO_2_ (a–d), Ir/TiC (e–h), and Ir/TiN (i–l). Color code: cyan (titanium), dark blue (fluorine), pink (iridium).

EDX line-scan measurements across the MEA cross-section demonstrate a concentration gradient of Ti-containing species from the anode toward the cathode side of the MEA (Figure S26). Importantly, the same behavior was reproduced in independent laboratory experiments and under different degradation protocols (0–2 V, 0–1.7 V, and 0.4 A cm^–2^), supporting the conclusion that degradation and redistribution of the TiC support represent the dominant degradation process in this system.

Despite the observed degradation and migration of Ti-containing species, the Ir nanoparticles appear to remain predominantly in the anode catalyst layer and continue to contribute to the electrochemical response (Figure 1e,j). Operando SAXS demonstrates that the average Ir particle size remains nearly constant through the AST, while postmortem TEM does not reveal significant Ir agglomeration. These observations suggest that TiC degradation does not proceed through instantaneous loss of the entire support particle. Instead, TiC progressively oxidizes and partially dissolves, leaving residual Ti-containing phases within the catalyst layer even after substantial loss of long-range TiC crystallinity. Consequently, the decrease in TiC diffraction intensity observed by WAXS reflects primarily the loss of crystalline TiC through a combination of oxidation, loss of crystalline order, and dissolution, rather than complete removal of all Ti-containing support material from the catalyst layer. Although the formation of a minor fraction of larger Ir agglomerates beyond the sensitivity of SAXS cannot be entirely excluded, neither operando SAXS nor postmortem TEM provides evidence for significant Ir coarsening during the AST. Despite the observed support degradation, the retention of measurable electrochemical activity throughout the AST (Figure 1e,j,k) further supports the conclusion that the majority of Ir nanoparticles remain electrochemically connected within the anode catalyst layer. Additional support for this interpretation is provided by the temperature-dependent HFR analysis (Section S19), which reveals substantially higher apparent activation energies for the TiC-based MEA (∼12 kJ mol^–1^) than for the TiO_2_- and TiN-based systems (∼5–7 kJ mol^–1^). While the latter values are close to those typically reported for Nafion-based proton transport, the significantly higher value observed for the TiC-based MEA suggests a stronger perturbation of the ion-conducting phase. However, the present measurements do not allow us to distinguish whether this perturbation originates predominantly from the membrane or from the ionomer within the catalyst layers, nor to establish Ti contamination as its direct cause.

ICP-OES analyses of the anode circuit water (Table S5) further support these findings. The cross-sectional EDX mappings (see Figure 6) provide a visualization of these effects. For Ir/TiO_2_, distinct layers corresponding to Ti, Ir, and fluorine (from Nafion) are clearly resolved, indicating good phase integrity and negligible interlayer diffusion. In contrast, Ir/TiC exhibits severe Ti redistribution across the membrane, while Ir/TiN shows moderate diffusion, consistent with its relative chemical stability inferred from XPS/XAS.

These structural and compositional findings correlate well with the electrochemical behavior observed in the DRT analysis of impedance spectra (Figures S18, S35–S38). The DRT spectra reveal two primary relaxation processes: a fast relaxation (10^–4^–10^–5^ s) attributed to ion transport within the catalyst layer and a slower relaxation (10^–1^–10^–2^ s) associated with charge transfer at the anode interface.^73–75^ Upon cycling, the Ir/TiC-based MEA exhibits a pronounced increase in the intensity of the ion transport-related relaxation process, indicating a rise in ion transport resistance, consistent with the pronounced Ti dissolution and redistribution observed for this MEA. The charge-transfer peak exhibits only minor variations, suggesting that structural degradation primarily affects ionic conductivity rather than catalytic kinetics.

For Ir/TiO_2_, the fast relaxation peak remains stable, confirming the preservation of ion-transport pathways. The gradual increase in charge-transfer resistance with cycling reflects a minor loss of electrochemical activity, possibly due to surface reorganization rather than support degradation.^74,75^ Conversely, the Ir/TiN MEA displays a distinctive evolution: the initially high ion-transport resistance decreases progressively with cycling up to 500 cycles, indicating improved ion transport within the catalyst layer. This trend is accompanied by a reduction in charge-transfer resistance, suggesting progressive catalyst activation. The improved transport properties are consistent with the observed decrease in contact angle from 82.0 ± 0.7° to 71.1 ± 3.3° after degradation (Figure S33), indicating enhanced wettability. Together with the XPS evidence of progressive TiN oxidation and the formation of Ti–O–N/TiO_*x*_ species (Figure 5a, Figure S34), these results suggest that partial surface oxidation may improve the interfacial properties of the catalyst layer.

Additional laboratory measurements, including repeated 0–2 V AST experiments and milder 0–1.7 V protocols, confirmed the reproducibility of the observed DRT evolution. Moreover, a weak additional relaxation process at more than 500 cycles became distinguishable for Ir/TiN and Ir/TiC, indicating the emergence of an additional transport-related processes within the catalyst layers (see Section S17 for detailed analysis).

Overall, the EDX and DRT results provide strong evidence that the MEAs’ degradation behavior is governed by the stability of the support material. The combined findings indicate that TiC suffers from severe oxidation, Ti dissolution, and ion-transport loss. TiN undergoes partial oxidation, resulting in moderate migration and surface activation. TiO_2_ remains structurally and chemically stable, maintaining efficient charge and ion transport. These complementary analyses establish a clear structure–property–performance relationship, linking support degradation to electrochemical impedance behavior and, overall, MEA durability.

## 4 Conclusions

This study employed *operando* SAXS and WAXS, along with complementary electrochemical and postmortem spectroscopic analyses, in an MEA-based PEM water electrolysis cell, to systematically investigate the degradation mechanisms of iridium-decorated titanium-based supports (TiO_2_, TiC, TiN) under accelerated stress-testing conditions.

The results show markedly different behaviors and interactions between the catalyst and the various supports. The Ir/TiO_2_ catalyst exhibits the highest electrochemical activity in the OER region throughout the degradation test. However, significant particle growth driven by Ostwald ripening results in a substantial reduction (∼40%) in the electrochemically active surface area and overall activity over the test duration, whereas the TiO_2_ support retains its structural integrity. In contrast, the Ir/TiC catalyst exhibits minimal Ir nanoparticle growth and a comparatively smaller loss of activity after the initial activation period, although its absolute activity in the OER region remains substantially lower than that of Ir/TiO_2_ throughout the AST. Nevertheless, the TiC support undergoes severe degradation, with an approximately 80% average reduction in the crystalline TiC diffraction peak area and pronounced Ti migration through the membrane, which could lead to long-term contamination. The Ir/TiN catalyst exhibits intermediate behavior, with moderate Ir nanoparticle growth and partial support degradation (∼50% crystallinity loss). While initial performance declines during early cycling, subsequent cycles show relatively stable electrochemical activity in the OER region, indicating partial resilience of the TiN support under harsh operational conditions. Both TiC and TiN supports underwent oxidation under the same conditions: TiC was mainly converted to rutile-like TiO_2_, while TiN formed a mixed Ti^3+^/Ti^4+^ oxide layer. These support transformations are accompanied by changes in the Ir oxidation state, such that while Ir remains predominantly oxidized on TiO_2_, oxidized Ir species coexist with a metallic Ir^0^ fraction on TiC- and TiN-supported catalysts, which could adversely affect charge transfer, catalyst utilization, and ultimately OER activity.

Overall, these findings highlight the complex relationship between Ir nanoparticle stability and support degradation in PEM water electrolyzers. The combined operando SAXS/WAXS measurements reveal that Ir nanoparticle stability and support stability evolve independently, emphasizing that preserving nanoparticle size alone is insufficient to ensure long-term catalyst durability. Operando characterization reveals that catalyst degradation pathways strongly depend on the nature of the support and cannot be inferred solely from electrochemical performance measurements. The combination of operando SAXS/WAXS with postmortem spectroscopy and microscopy provides direct insight into the evolution of both catalyst nanoparticles and support materials under realistic operating conditions.

Most importantly, the present results demonstrate that catalyst durability in PEMWEs cannot be evaluated solely on the basis of Ir nanoparticle stability. Although Ir/TiC exhibits the highest resistance toward nanoparticle growth, severe degradation of the TiC support leads to oxidation, loss of crystallinity, and Ti migration across the MEA. In contrast, TiO_2_ provides excellent structural robustness but does not sufficiently suppress Ir nanoparticle growth. These findings reveal that maximizing nanoparticle stability alone does not guarantee long-term catalyst durability. Future catalyst–support development should therefore simultaneously target suppression of Ir ripening, resistance of the support toward oxidation and dissolution, minimization of cation migration through the membrane, and preservation of electronic conductivity under anodic PEMWE conditions. The results further suggest that protected conductive supports and mixed oxide–nitride architectures represent promising directions for the development of durable low-Ir PEMWE anodes.

## Supplementary Material



## Data Availability

Data will be available in the repository on Zenodo doi.org/10.5281/zenodo.18338229.

## Notes

Additional experimental and data analysis details; operando electrochemical, SAXS/WAXS, and PEIS/DRT results; SAXS modeling and particle size distribution analysis; postmortem XANES, SEM/EDX, HR-TEM, WAXS, and ICP analyses; laboratory electrochemical durability and reproducibility measurements; XPS analysis after RDE testing; fluoride and contact-angle measurements; lateral resistance measurements; and temperature-dependent HFR and apparent activation-energy analysis (PDF).
