# Supplementary material for: Operando and Postmortem Study of Iridium-Decorated Titanium-Based Catalysts in PEM Water Electrolysis
Source: J Am Chem Soc. 2026 Sep 23;148(39):41838–50. doi: 10.1021/jacs.6c04230 (PMC13651515; doi:10.1021/jacs.6c04230)
Supplement: Supplementary file 1 [file ja6c04230_si_001.pdf]

Operando and post-mortem study of iridium-decorated titanium-based catalysts in PEM water electrolysis

Lucinda Blanco-Redondo[a], Yevheniia Lobko*[a], Marco Bogar[b], Yurii Yakovlev[a], Jaroslava Nováková[a], Simone Pollastri[c,d], Alina Madalina Darabut[a], Miquel Gamón Rodríguez[a], Giovanna Marussi[e], Matteo Crosera[e], Michal Mazur[f], Milan Dopita[g], Viacheslav Kalinovych[a], Roberto Biagi[c,h,i], Heinz Amenitsch[d,j], Rodolfo Taccani[b], Iva Matolínová[a]

[a]Department of Surface and Plasma Science, Faculty of Mathematics and Physics, Charles University, V Holešovičkách 2, 180 00, Prague 8, Czech Republic
e-mail: yevheniia.lobko@matfyz.cuni.cz
[b]Department of Engineering and Architecture, University of Trieste, Via Alfonso Valerio 6/1, 34127, Trieste, Italy
[c]Department of Physics, Computer Science and Mathematics, University of Modena and Reggio Emilia, 41125, Modena, Italy
[d]ELETTRA - Sincrotrone Trieste S.C.p.A., SS 14 - km 163,5, 34149, Basovizza, Trieste, Italy
[e]Department of Chemical and Pharmaceutical Sciences, University of Trieste, Via Giorgeri 1, Trieste 34127, Italy
[f]Department of Physical and Macromolecular Chemistry, Faculty of Science, Charles University, Hlavova 8, 128 00, Prague 2, Czech Republic
[g]Department of Condensed Matter Physics, Faculty of Mathematics and Physics, Charles University, Ke Karlovu 5, 121 16, Prague 2, Czech Republic
[h]Istituto Nanoscienze (NANO-S3), Consiglio Nazionale delle Ricerche (CNR), via G. Campi 213/a, Modena, 41125, Italy
[i]Centro H2-MORE, University of Modena and Reggio Emilia, 41125, Modena, Italy
[j]Graz University of Technology, Institute of Inorganic Chemistry, Stremayrgasse 9, 8010, Graz, Austria

---

S1. *In-situ* water electrolyzer cell for *operando* measurements

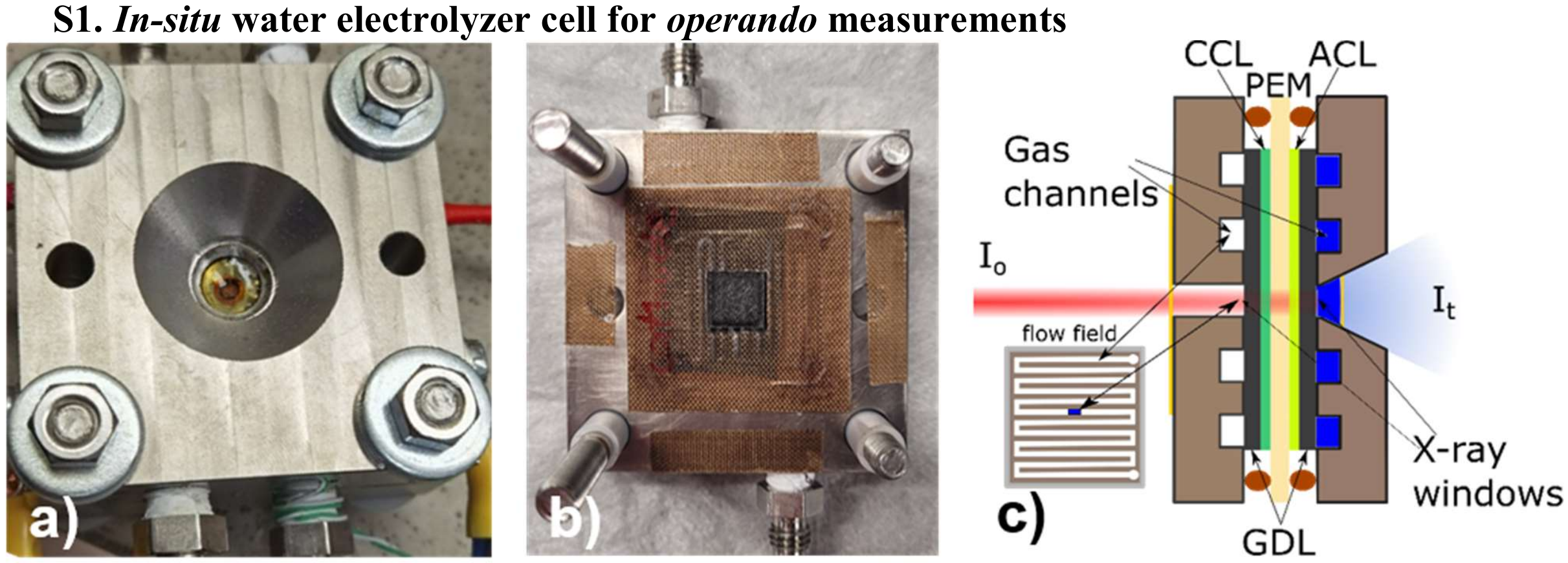

Fig. S1. The electrochemical cell. (a,b) Image of the electrochemical cell used for the experiment, and (c) scheme of the electrochemical cell highlighting the X-ray: incident beam intensity (I_0), transmitted intensity (I_t), Cathode and Anode Catalyst Layers (CCL and ACL), and Gas Diffusion Layer (GDL).

More information about the setup can be found in [1,2].

OPERANDO MEASUREMENTS

S2. Evolution of OER activity (detail for Section 3.1.3)

The evolution of the electrochemical activity in the OER region for each MEA during the AST is shown in **Fig. 1a–c**, while the corresponding chronoamperometric responses are presented in **Fig. S2b**.

For Ir/TiO_2, the current density at 1.525 V initially increases during the first 250 cycles (**Fig. 1a, Fig. 1k**), consistent with the activation of Ir nanoparticles. After this point, the current density in OER region declines steadily from 129.2 to 75.7 mA·cm^{-2} (Δ = −41.3%), mirroring the loss of Ir redox charge (**Section 3.1.2**). The degradation is also reflected in the chronoamperometry profiles (**Fig. 1g, Fig. S2b**), indicating substantial structural or chemical deterioration under AST cycling.

* Corresponding author.
E-mail address: yevheniia.lobko@matfyz.cuni.cz

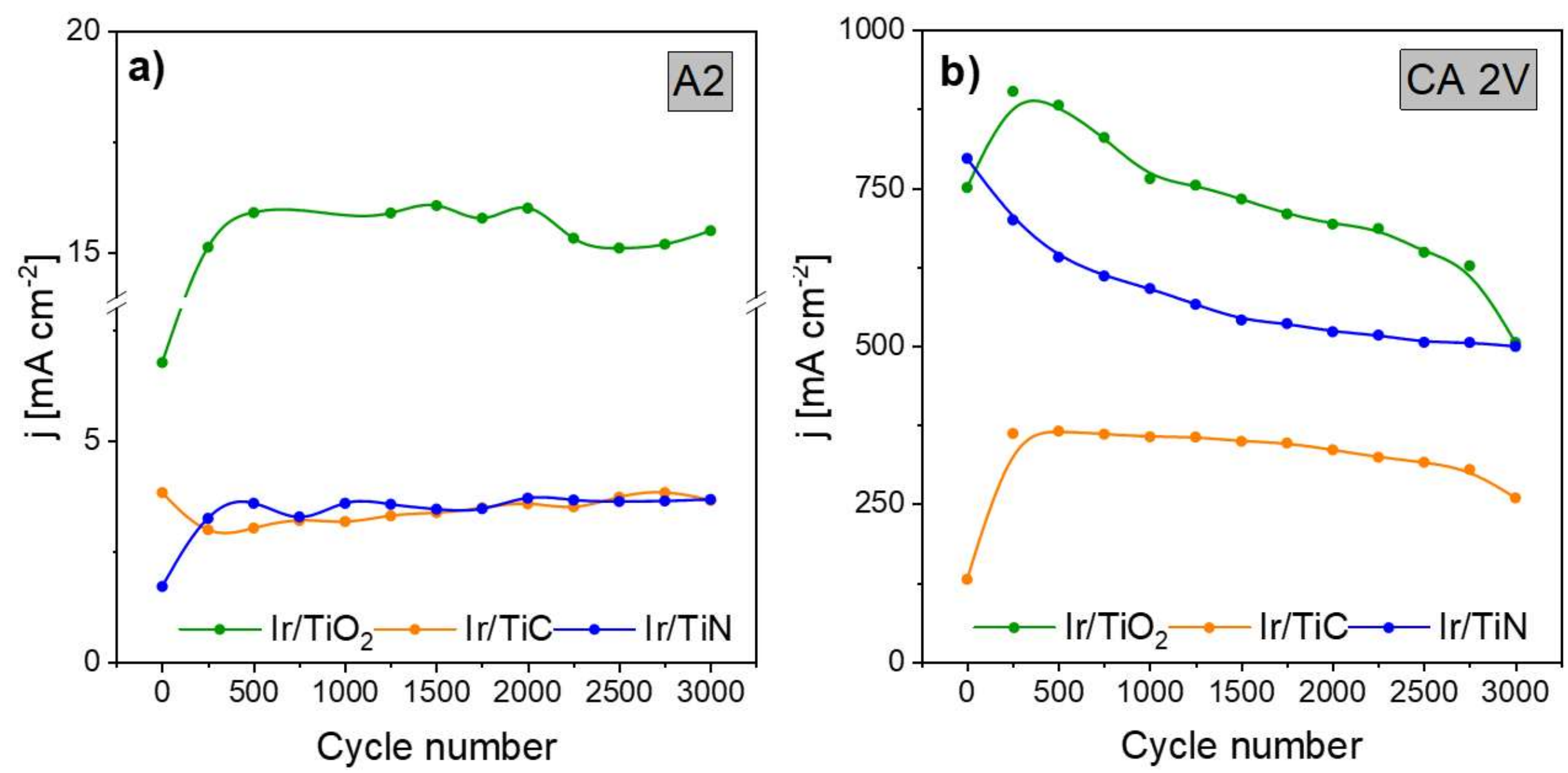

Fig. S2. Evolution of electrochemical parameters during the *operando* AST for Ir/TiO_2, Ir/TiC, and Ir/TiN MEAs: (a) anodic peak A2 at 1.17 V, and (b) current profile from chronoamperometry recorded at 2.0 V.

For Ir/TiC, the current density in OER region increases up to ~ 500 cycles (**Fig. 1b, Fig. 1k**), reaching ~ 50 mA·cm^{-2}. This prolonged activation correlates with increasing A2 and C2 peak charges, suggesting improved accessibility of Ir sites. After reaching its maximum, the activity gradually decreases to 37 mA·cm^{-2} (Δ = −26%), as seen in both OER and chronoamperometry curves (**Fig. 1h, Fig. S2b**). The degradation is moderate relative to Ir/TiO_2.

The Ir/TiN system exhibits a sharp initial drop during the first 250 cycles, from 107.1 to 64.7 mA·cm^{-2} (**Fig. 1c, Fig. 1k**), likely due to the rapid surface restructuring of the nitride support. Afterward, both the OER current and chronoamperometry response (**Fig. 1i, Fig. S2b**) remain nearly constant throughout the remainder of the AST, indicating the formation of a more stable catalyst state.

Across the entire AST, absolute activity follows the order: Ir/TiO_2 > Ir/TiN > Ir/TiC. However, normalized stability favors Ir/TiC and Ir/TiN. These trends correlate with the evolution of redox features (**Section 3.1.2**) and highlight the decisive role of the support in determining catalyst activation and durability under aggressive cycling.

S3. SAXS data analysis: operando study

MEA modelling

SAXS patterns were fitted using an analytical model built according to the approach previously reported [1]. The model was built by summing all of the contributions produced from each of the layers of the MEA:

$$I(q) = I_N(q) + I_{SUP}(q) + I_{CP}(q) + I_{TL}(q)$$

The contribution from the cathode electrode was assumed to be negligible, as the forward scattering intensity of the electrode is at least one order of magnitude lower than the contribution from the support only, as shown in **Fig. S3**. Moreover, by following the approach already used in previous works [1,2], to avoid the superimposition of the scattering contributions originated by the two different particle populations deposited at the two electrodes, a strongly asymmetrical catalyst loading was used: being the catalyst loading at the anode 20 times higher than the catalyst loading at the cathode, the contribution of Pt nanoparticles can be considered negligible with respect the contribution from both the Ir nanoparticles (similar in size but bigger in number) and the support nanoparticles (bigger in size, as the forwarded scattering intensity scales with the particle volume).

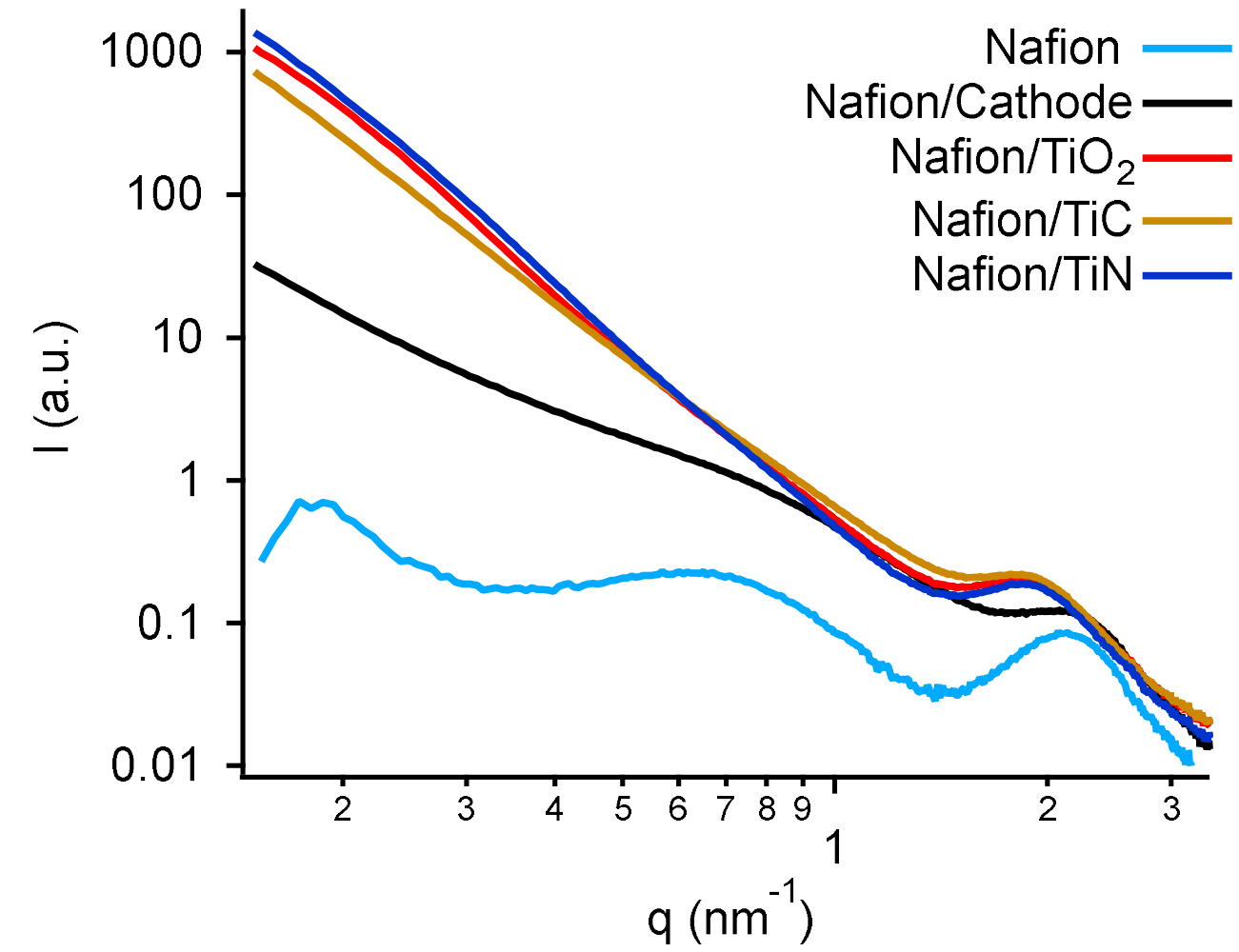

Fig. S3. Comparison of the scattering patterns of the layers composing the MEA. Scattering patterns of the bare Nafion membrane are compared with the bilayers composed of Nafion and the cathodic electrode (composed of Pt nanoparticles deposited onto a Vulcan substrate) and the bilayers composed of Nafion and each of the three studied supports: TiO_2, TiC, and TiN.

Bare Nafion scattering pattern can be fit by using two Voigt peaks [3–7]:

$$I_N(q) = B + V\left(I_{MK}, \sigma_{S_{MK}}, q_{MK}, s_{MK}, q\right) + V\left(I_{IP}, \sigma_{S_{IP}}, q_{IP}, s_{IP}, q\right),$$

where the subscripts *MK* and *IP* respectively represent the matrix knee and the ionomer peak [8,9], while I_X, σ_X, q_X, and s_X, respectively, represent peak intensity, width, position, and shape.

To model the supported-catalyst nanoparticles, the development of the analytical model was firstly referred to the works of Gommes, Asset, and co-workers [10–12], who discussed the importance of considering the cross-correlation term for modelling systems composed of a smaller nanoparticle population dispersed on the surface of larger support nanoparticles or into nanoporous supports. In a previous work of us addressing degradation in MEA for fuel cells, we discussed some conditions in which the cross-correlation term can be neglected [1], in agreement with some of the approximations theoretically presented by Gommes *et al.* [1,12]. In particular, to consider the cross-correlation term negligible, two conditions need to be satisfied: the size of the supporting particle needs to be bigger than the size of the decorating particle, and the scattering contrast between the two materials needs to be large enough. While these conditions can be satisfied by the electrodes used in PEM fuel cells, this is not the case for the anode electrodes studied in this work. In fact, for Pt/Vulcan electrodes, the difference from the electron density of carbon and the electron density of water (which can be considered as a background for measurements in *operando* for electrolysers) is equal to about 0.5 $F{\cdot}cm^{-3}$ [11], which is quite less than the difference of electron density from Pt nanoparticles and water, which is equal to about 8.1 $F{\cdot}cm^{-3}$ [11]. While Ir and Pt nanoparticles are characterized by similar electron density values (8.5 $F{\cdot}cm^{-3}$ for Ir), support materials are characterized by larger electron density differences with respect to water: 1.3, 1.8, and 1.9 $F{\cdot}cm^{-3}$ for TiO_2, TiC, and TiN, respectively. Analysis of the TEM images (Fig. S4) indicates that the support nanoparticles exhibit sizes up to approximately 20 nm, whereas the decorating particles possess diameters not exceeding 2 nm.

Assuming average diameters of the support and decorating nanoparticles of 30 and 2 nm, respectively, a polydispersity index of 0.15 and 0.1 for support and decorating nanoparticles respectively (both within the lognormal distribution), and the average presence of 100 decorating particles for each support one, the deconvoluted components of the form factor representing a single system composed by support and decorating nanoparticles is compared in **Fig. S5**.

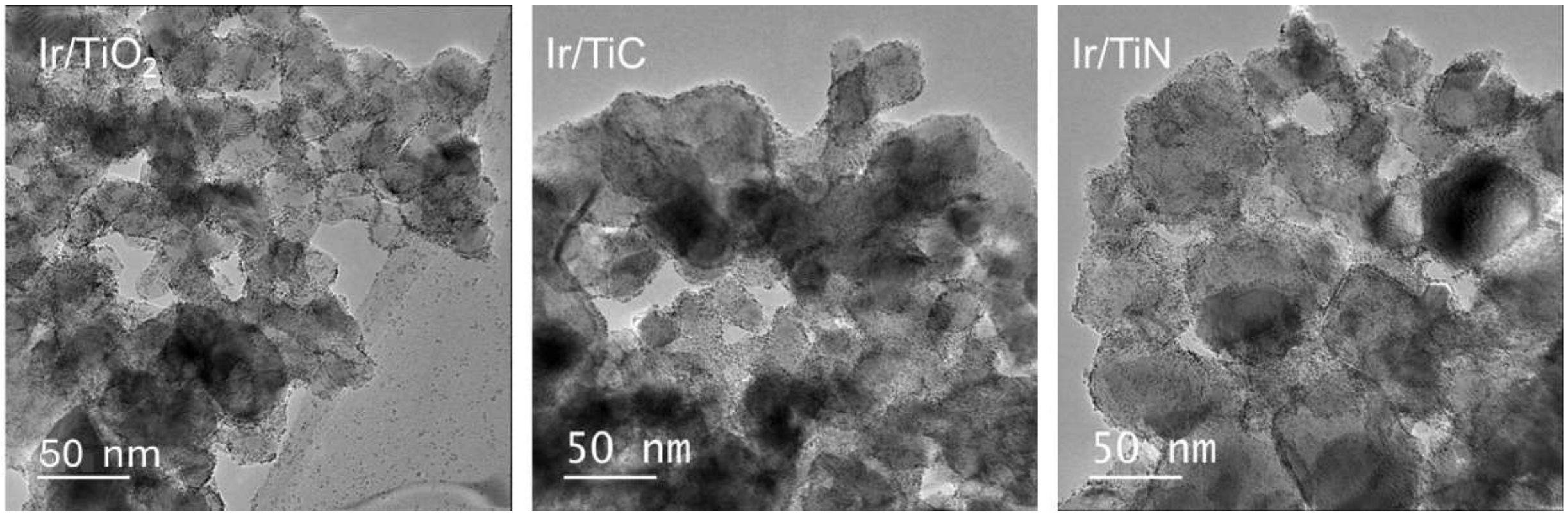

Fig. S4. TEM images, used to estimate the average support size for evaluating the impact of the cross-correlation term in the analytical model used for fitting.

As observed, both the support and the cross-correlation term occupy the same range of q-values; moreover, the cross-correlation term is about one order of magnitude lower in intensity than the support contribution. In these regards, a set of particles following the Schultz distribution [6,7] was used to model together both the support and the cross-correlation term (which is considered to influence in a minor manner), exploiting the advantages of a lighter computation and the possibility to sum the contribution from both support and catalyst nanoparticles independently. Moreover, it is worth noting that the detectability of any component feature at low q-values is limited by the presence of the GDL at the cathode and by the Porous Transport Layer (PTL) at the anode [1]. Thus, support was modelled with a set of particles following the Schultz distribution:

$$I_{SUP} = I_S \cdot P(D_S, \sigma_S, q)$$

where I_S represents the forward scattering probability, and D_S and σ_S represent the mean and the standard deviation of the distribution, respectively. Due to the proximity of the form factor to the lower detection value and the approximation introduced (where I_{SUP} accounts for both support and cross-correlation terms), this term is supposed to mainly provide qualitative information about changes involving support nanoparticles.

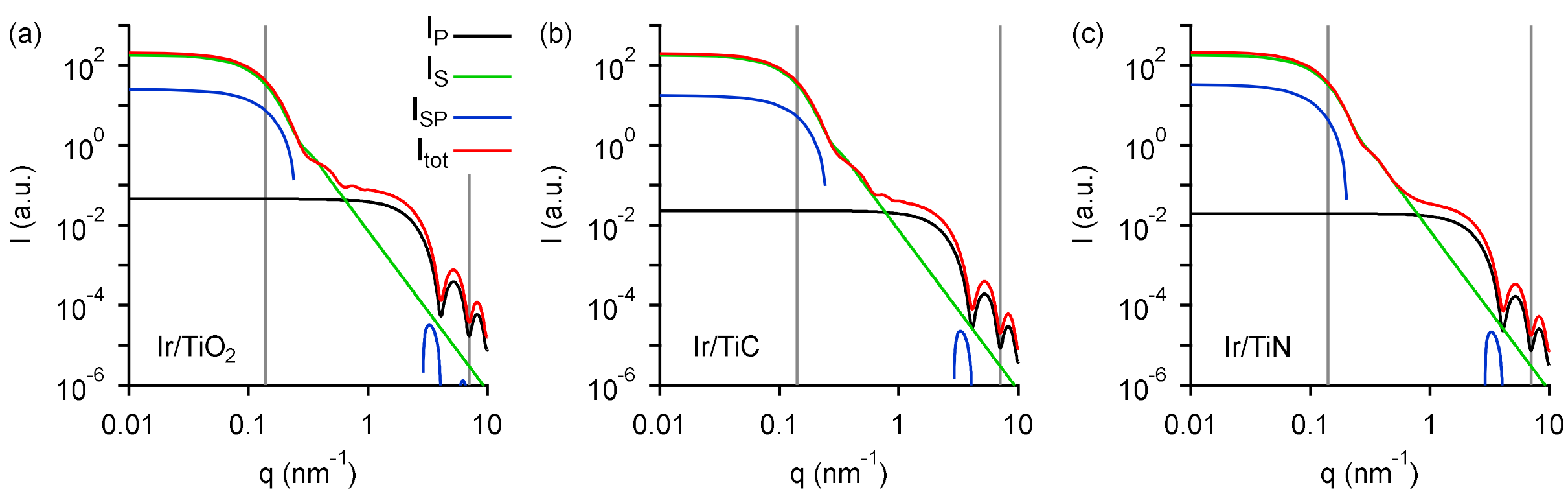

Fig. S5. Evaluation of the cross-correlation term for construction of the SAXS analytical model. The total forward scattering probability of the system, composed of support and decorating nanoparticles (I_{tot}), is deconvoluted into the three terms composing: a form factor used to represent the support nanoparticles (I_S), a term representing the set of decorating nanoparticles (I_P), and the cross-correlation term (I_{SP}). Vertical grey lines highlight the q-range probed experimentally.

As anticipated, catalyst nanoparticles were modelled by means of the product of a form factor by a structure factor

$$I_{CP} = I_P \cdot P(D_P, \sigma_P, q) \cdot S(R_{SHS}, \varphi_{SHS}, \lambda, \varepsilon, q)$$

As a form factor a set of spheres following the Schultz distribution was chosen (analogously: I_P, D_P, and σ_P respectively represent the forwarded scattering probability, the mean particle diameter and the its standard deviation); as a structure factor, the Sticky-Hard Sphere model was used to check the influence of a near-range order on the scattering pattern: here R_{SHS} represents the Sticky-Hard-Sphere radius, φ_{SHS} represents the apparent volume fraction, while λ and ε represent the width and the depth

of the potential well, respectively [13,14]. From the Sticky-Hard-Sphere radius, the minimum interparticle distance was calculated ($d_{min} = 2 \cdot R_{SHS}$). Both λ and ε were kept fixed to the values found by fitting the pattern in pristine conditions.

Finally, due to the bigger size of the structures composing the transport layers of the MEA (GDL and PTL), which are well outside of the detection range, a power law was used to model the contribution from both of them:

$$I_{TL}(q) = \frac{C}{q^p}$$

To retrieve the starting values for support particle populations, the bilayers composed of the Nafion membrane and each of the three supports tested here (shown in **Fig. S3**) were initially fitted with an analytical model composed of one particle population only and one Voigt peak, as the matrix knee could not be reliably detected. Fit results (**Fig. S6**) allowed to quantify mean particle size equal to 17.3 ± 0.01 nm, 54.3 ± 0.01 nm, and 14.1 ± 0.01 nm for TiO_2, TiC, and TiN, respectively, and distribution width equal to 8.0 ± 0.01 nm, 15.3 ± 0.01 nm, and 6.4 ± 0.01 nm for TiO_2, TiC, and TiN, respectively. These values were used as starting values to model the support particles in modelling the full MEA.

Particle size distribution analyses

To determine the prevailing degradation factor affecting Ir nanoparticles, a criterion previously developed for modelling degradation in catalyst model systems for fuel cells [15] was used. There, by following the evolution of mean particle size, distribution width, and interparticle distance, it was possible to distinguish among particle coalescence, Ostwald ripening, and dealloying; moreover, when particle coalescence was detected, the shape of the particle size distribution was observed to be skewed towards higher values, and calculated skewness resulted in positive values increasing over time. On the contrary, when Ostwald ripening was found to prevail, the shape of particle size distribution was observed to be more symmetrical, with skewness not increasing or slightly decreasing. By considering the chemical and morphological differences characterizing the morphology of the samples used to develop the model from the catalyst layer studied in this work, and the different degradation protocol used, it is clear that from the temporal evolution of the morphological parameters of the analytical model, no reliable analogies can be made. Nonetheless, the observed tendency of particle size distribution shape, to evolve towards more symmetrical shapes during the stress test (**Fig. S10a-c**), together with the decreasing trend of the calculated skewness over time (**Fig. S10d-f**), in analogy with what previously reported [15], and supported by the experimental and theoretical results obtained by Kregar and co-workers [16,17], it was considered reliable to address at Ostwald ripening as main degradation phenomena taking place over time.

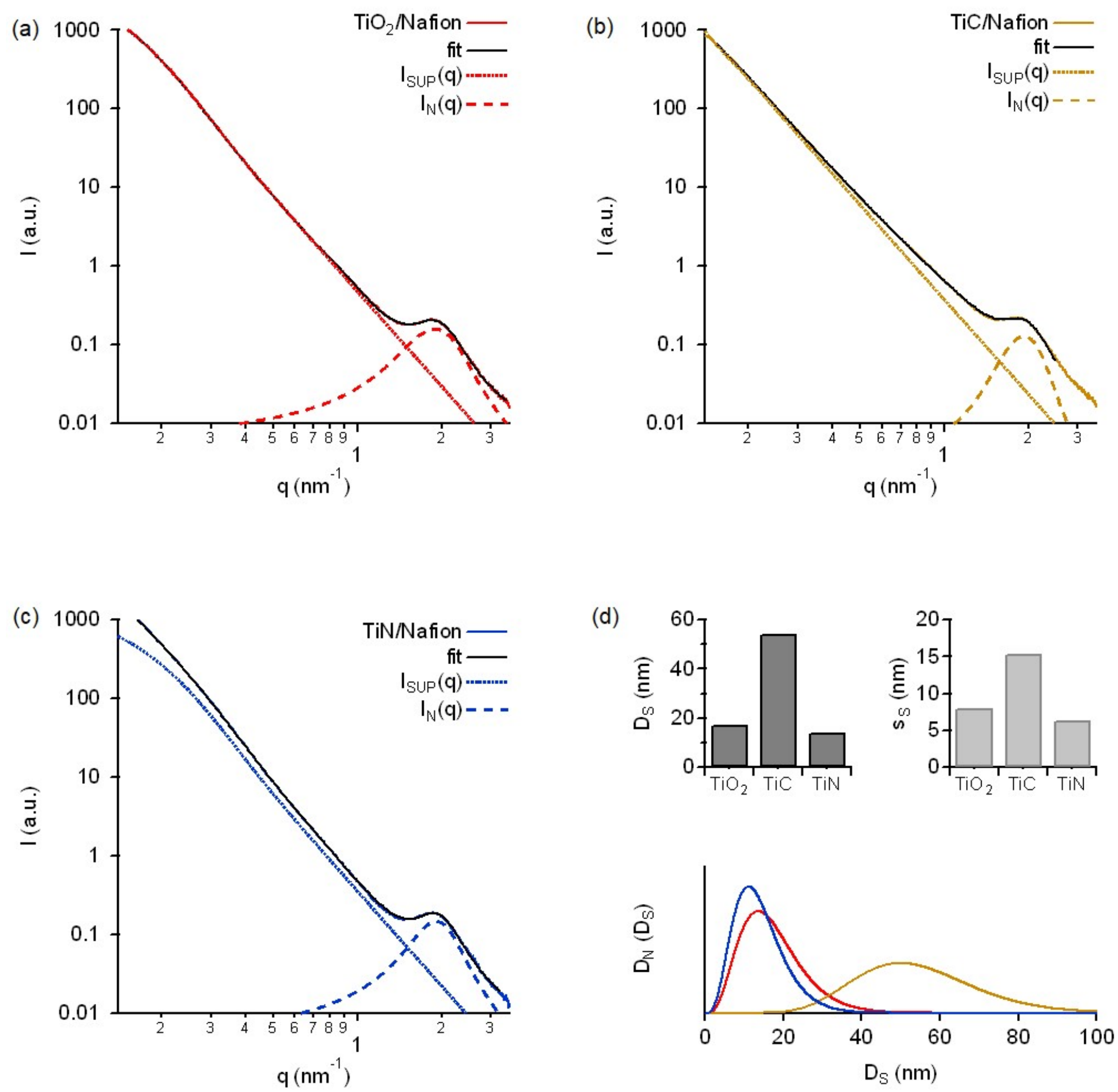

Fig. S6. **Support SAXS characterization**. SAXS patterns recorded *ex situ* from the bilayers composed of Nafion and each of the three support materials here studied: (a) TiO_2, (b) TiC, and (c) TiN. Recorded patterns are plotted together with the fit and the deconvoluted components of the model used for data fitting ($I(q) = I_N(q) + I_{SUP}(q)$). At room temperature and relative humidity, only the ionomer peak of Nafion was detected. (d) Summary of the main fit results, expressed in terms of mean particle size (D_S) and width (σ_S) within the Schultz distribution; the number weighted size distribution is shown below.

SAXS data fitting

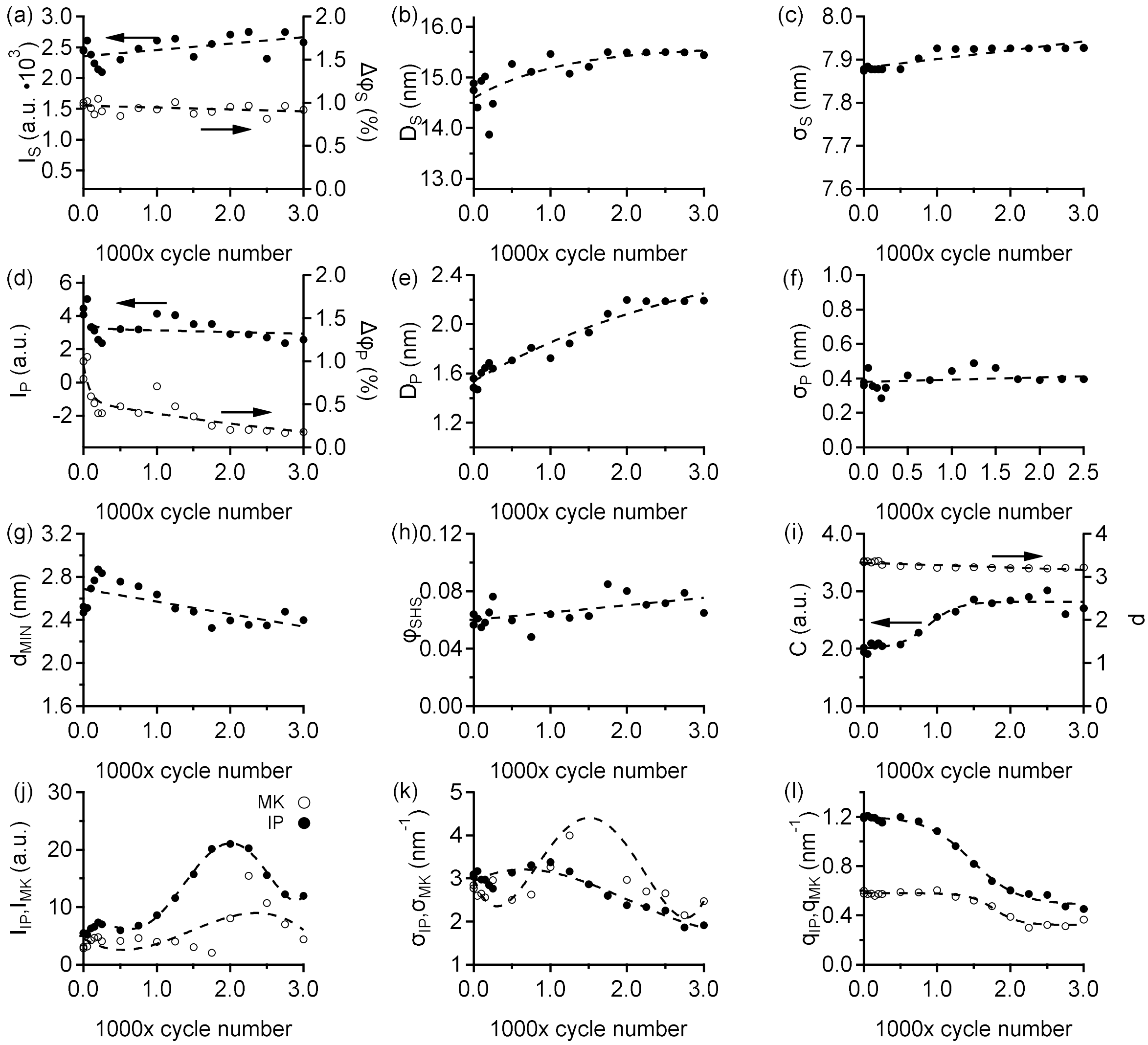

Fig. S7. Complete results from fitting time-resolved SAXS patterns measured from the MEA made with TiO_2 support (**Fig. 2a** of the manuscript). Support related parameters: (a) forwarded scattering probability (I_S) and relative variation of calculated particle volume fraction ($\Delta\varphi_S$), (b) mean particle diameter (D_S), and (c) width (σ_S) within the Schultz distribution. Catalyst-related parameters: (d) forwarded scattering probability (I_P) and relative variation of calculated particle volume fraction ($\Delta\varphi_P$), (e) mean particle diameter (D_P) and (f) width (σ_P) within the Schultz distribution, (g) minimum interparticle distance and (h) apparent volume fraction (φ_{SHS}). Potential well width (λ) and depth (ε) were retrieved from fitting the first pattern and then kept fixed at 0.15 and 3.80, respectively. Mass transport layers related parameters: (i) forward scattering probability (C) and power law exponent (p). Nafion-related parameters: (j) forwarded scattering probability of the ionomer peak (I_{IP}), (k) peak width (σ_{IP}), and (l) position (q_{IP}); the shape parameters s_{MK} and s_{IP} were kept fixed to the initial values of 0 and 0.44. *Note:* the dashed lines are a guide for the eyes only.

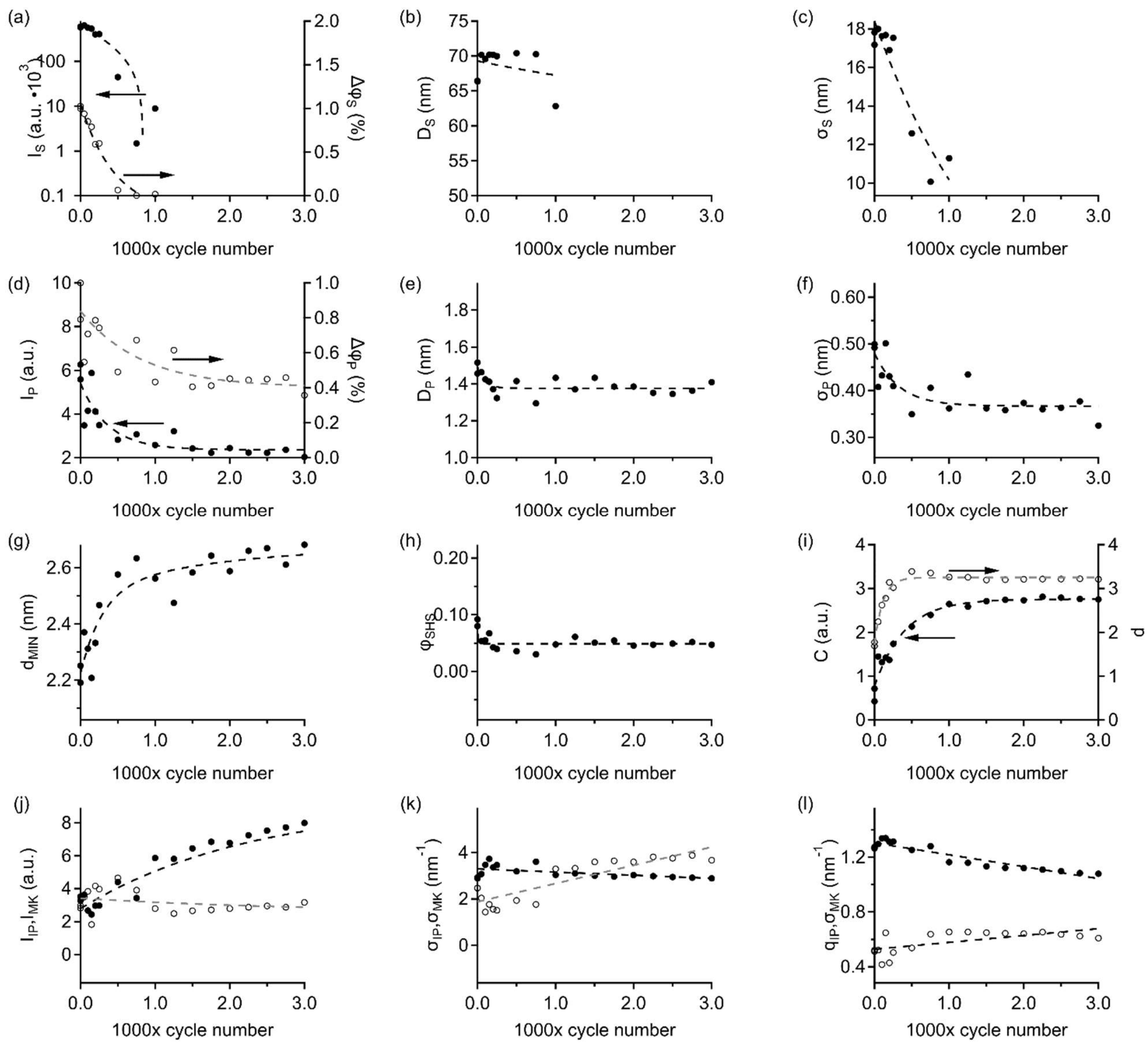

Fig. S8. Complete results from fitting time-resolved SAXS patterns measured from the MEA made by using TiC support (**Fig. 2b** of the manuscript). Support related parameters: (a) forwarded scattering probability (I_S) and relative variation of calculated particle volume fraction ($\Delta\varphi_S$), (b) mean particle diameter (D_S), and (c) width (σ_S) within the Schultz distribution. Catalyst-related parameters: (d) forwarded scattering probability (I_P) and relative variation of calculated particle volume fraction ($\Delta\varphi_P$), (e) mean particle diameter (D_P) and (f) width (σ_P) within the Schultz distribution, (g) minimum interparticle distance and (h) apparent volume fraction (φ_{SHS}). Potential well width (λ) and depth (ε) were retrieved from fitting the first pattern and then kept fixed at 0.16 and 3.67, respectively. Mass transport layers related parameters: (i) forward scattering probability (C) and power law exponent (p). Nafion-related parameters: (j) forwarded scattering probability of the ionomer peak (I_{IP}), (k) peak width (σ_{IP}), and (l) position (q_{IP}). Nafion-related parameters: (j) forwarded scattering probability of the ionomer peak (I_{IP}), (k) peak width (σ_{IP}), and (l) position (q_{IP}); the shape parameters s_{MK} and s_{IP} were kept fixed to the initial values of 0 and 0.44. Note: the dashed lines are a guide for the eyes only.

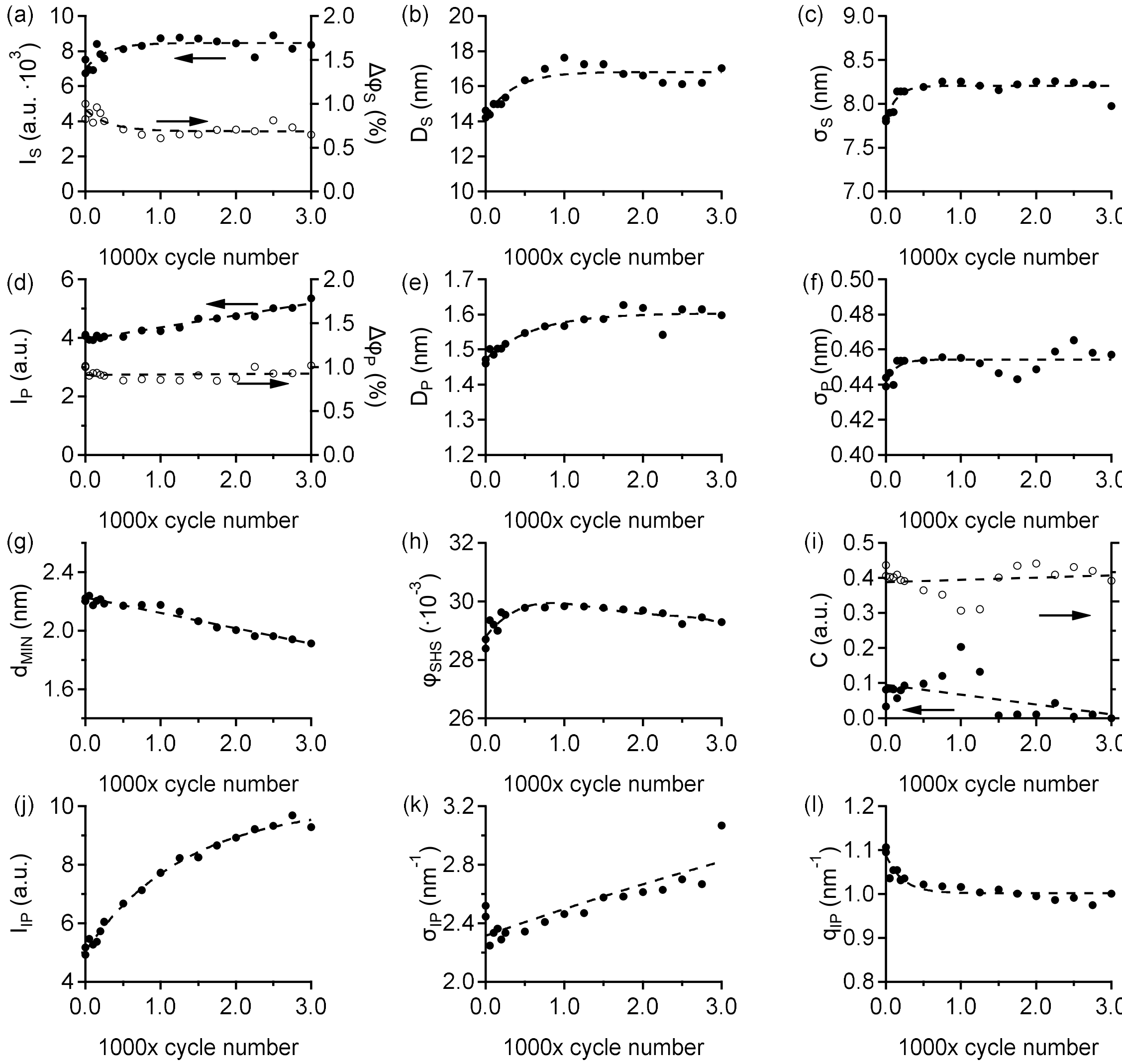

Fig. S9. Complete results from fitting time-resolved SAXS patterns measured from the MEA made by using TiN support (**Fig. 2c** of the manuscript). Support related parameters: (a) forwarded scattering probability (I_S) and relative variation of calculated particle volume fraction ($\Delta\varphi_S$), (b) mean particle diameter (D_S), and (c) width (σ_S) within the Schultz distribution. Catalyst-related parameters: (d) forwarded scattering probability (I_P) and relative variation of calculated particle volume fraction ($\Delta\varphi_P$), (e) mean particle diameter (D_P) and (f) width (σ_P) within the Schultz distribution, (g) minimum interparticle distance and (h) apparent volume fraction (φ_{SHS}). Potential well width (λ) and depth (ε) were retrieved from fitting the first pattern and then kept fixed at 0.21 and 6.85, respectively. Mass transport layers related parameters: (i) forward scattering probability (C) and power law exponent (p). Nafion-related parameters: (j) forwarded scattering probability of the ionomer peak (I_{IP}), (k) peak width (σ_{IP}), and (l) position (q_{IP}); the shape parameter (s_{IP}) was kept fixed to the initial value of 0.46. The matrix knee was not detected. *Note:* the dashed lines are a guide for the eyes only.

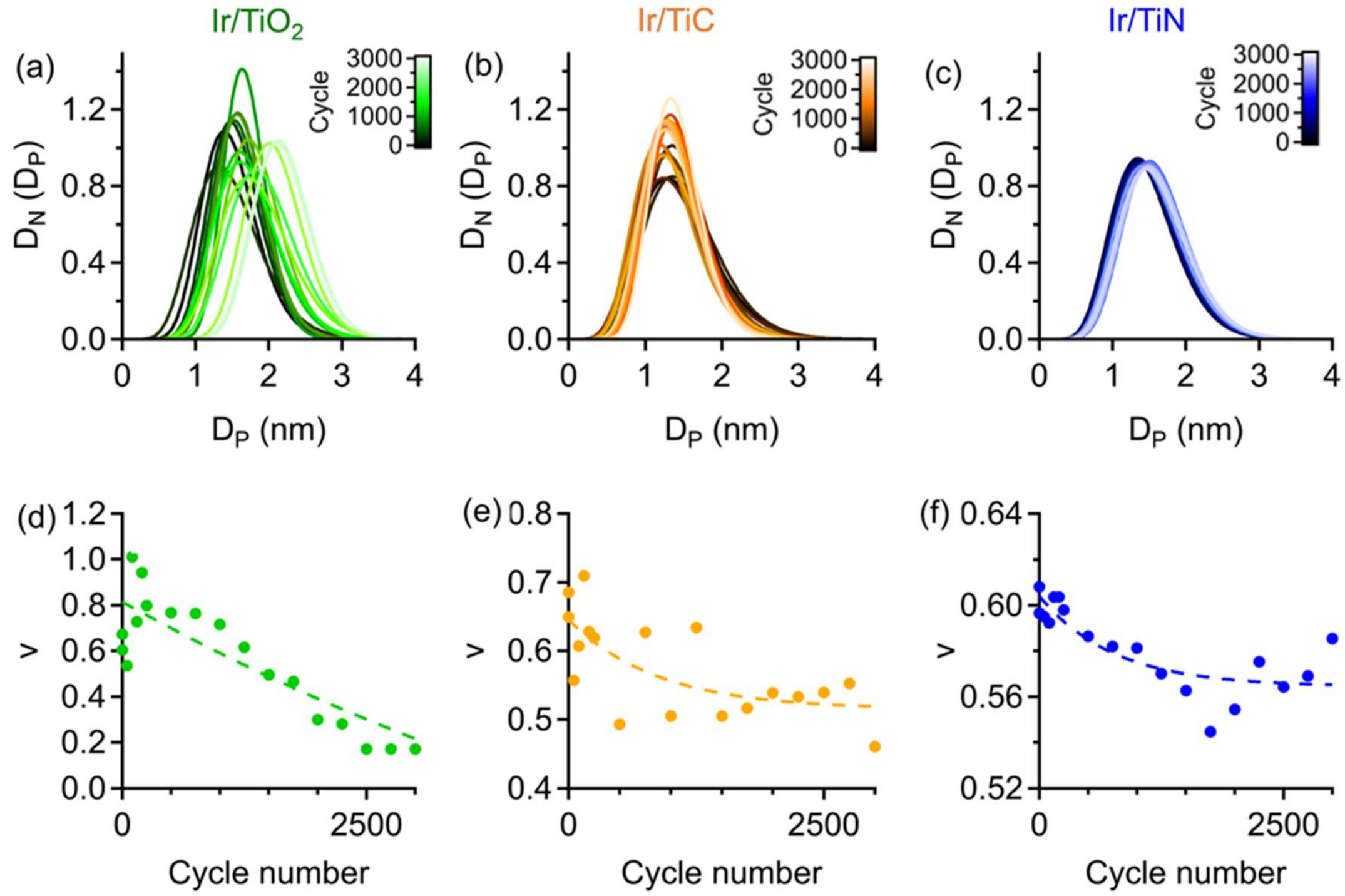

Fig. S10. Time-resolved calculated size distributions of the catalyst particle population for (a) Ir/TiO_2, (b) Ir/TiC, and (c) Ir/TiN. From the particle size distributions, the distribution skewness was calculated for (d) Ir/TiO_2, (e) Ir/TiC, and (f) Ir/TiN. *Note:* the dashed lines are a guide for the eyes only.

S4. WAXS data analysis: operando study

Fig. S11 illustrates the peak deconvolution carried out for each MEA, together with phase identification (**Table S1:** Ir/TiO_2, **Table S2**: Ir/TiC, **Table S3:** Ir/TiN), recorded at starting conditions, *in operando*.

In addition to the diffraction peaks originating from the catalyst support, reflections from the Ti mesh of the porous transport layer (PTL), the carbon gas diffusion layer (GDL), and water/Nafion were also observed.

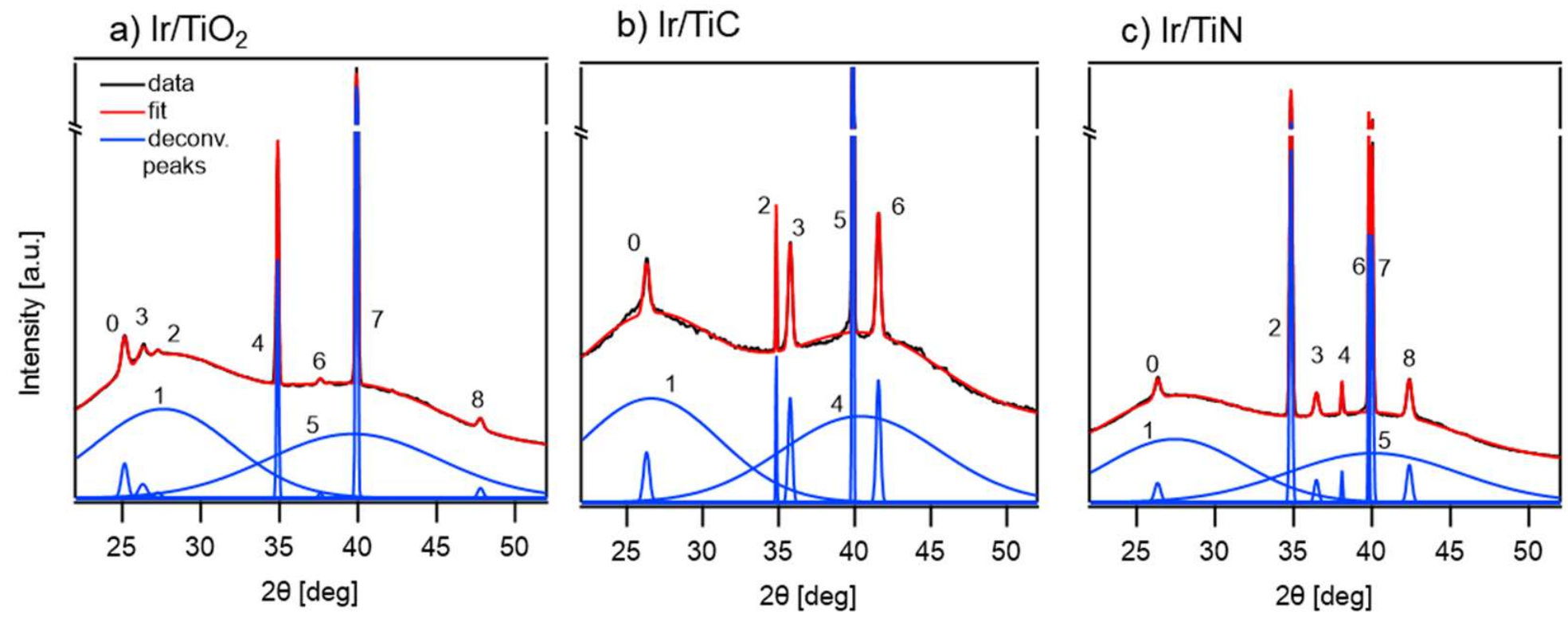

Fig. S11. Peak fitting and deconvolution of the X-ray diffraction reflections of MEAs containing (a) Ir/TiO_2, (b) Ir/TiC, and (c) Ir/TiN catalysts.

Table S1. XRD peaks identification for Ir/TiO_2. (R: rutile, A: anatase).

**Peak**	**Layer**	**(hkl)**	**2*θ* [º]**	**Ref.**	**Peak**	Layer	(hkl)	2*θ* [º]	Ref.
**p0**	TiO_2 A.	(101)	25.2	[18]	**p5**	Nafion	-	39.6	[19]
**p1**	Water	-	27.6	[19]	**p6**	Ti mesh	(002)	37.6	[20]
**p2**	TiO_2 R.	(110)	27.3	[18]	**p7**	Ti mesh	(111)	39.9	[20]
**p3**	GDL	-	26.3	[21]	**p8**	TiO_2 A.	(200)	47.8	[18]
**p4**	Ti mesh	(100)	34.9	[20]					

Table S2. XRD peaks identification for Ir/TiC.

**Peak**	**Layer**	**(hkl)**	**2*θ* [º]**	**Ref.**	**Peak**	Layer	(hkl)	2*θ* [º]	Ref.
**p0**	GDL	-	26.3	[21]	**p4**	Nafion	-	40.4	[19]
**p1**	Water	-	26.6	[19]	**p5**	Ti mesh	(111)	39.9	[20]
**p2**	Ti mesh	(100)	34.8	[20]	**p6**	TiC	(200)	41.6	[22]
**p3**	TiC	(111)	35.8	[22]					

Table S3. XRD peaks identification for Ir/TiN.

**Peak**	**Layer**	**(hkl)**	**2*θ* [º]**	**Ref.**	**Peak**	**Layer**	**(hkl)**	**2*θ* [º]**	**Ref.**
**p0**	GDL	-	26.3	[21]	**p5**	Nafion	-	40.1	[19]
**p1**	Water	-	27.4	[19]	**p6**	Ti mesh	(111)	40.0	[20]
**p2**	Ti mesh	(100)	34.8	[20]	**p7**	Ti mesh	(111)	39.8	[20]
**p3**	TiN	(111)	36.5	[23]	**p8**	TiN	(200)	42.4	[23]
**p4**	Ti mesh	(002)	38.1	[20]					

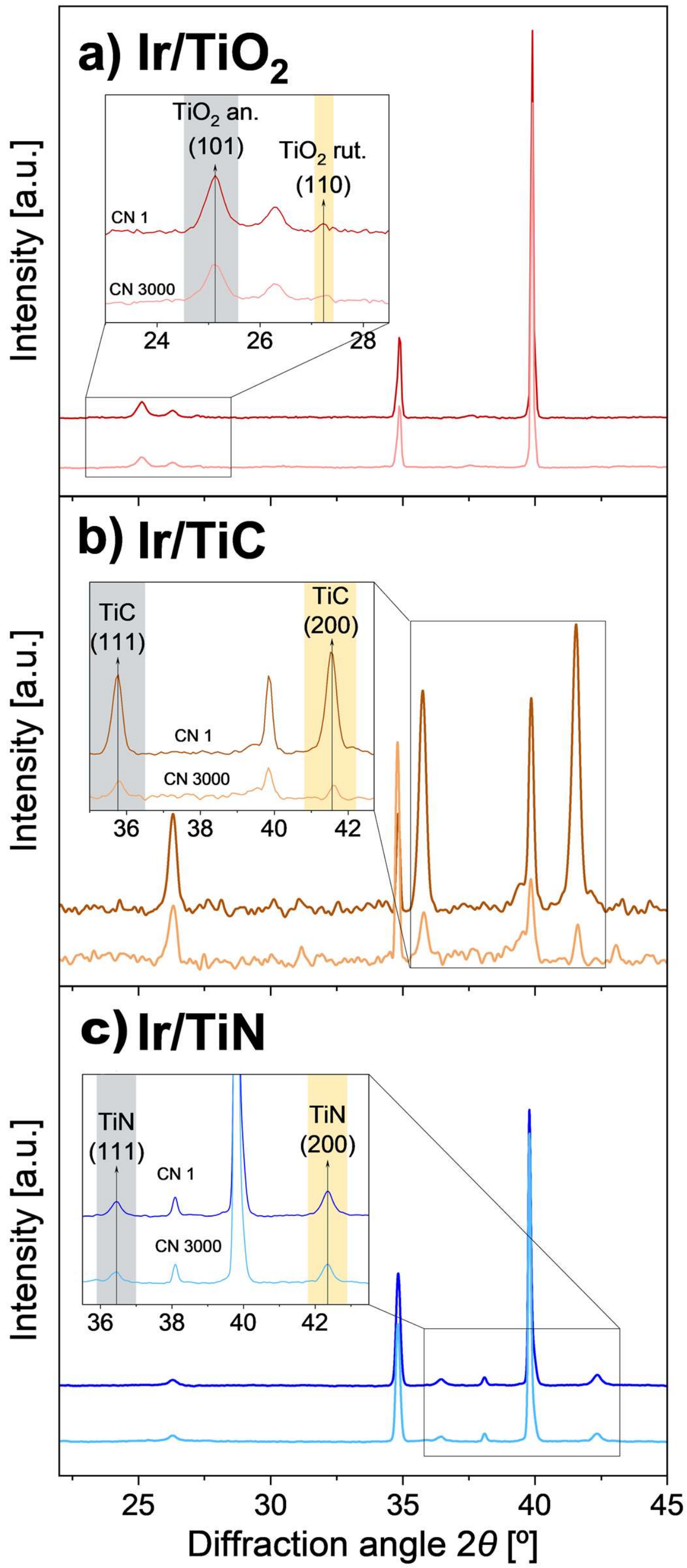

Fig. S12. WAXS patterns recorded in *operando* condition at the beginning of the AST (darker color) and after 3000 cycles (lighter color) of ASTs for MEAs loaded with (a) Ir/TiO_2, (b) Ir/TiC, and (c) Ir/TiN catalysts. Note: the time-resolved evolution of the patterns is shown in **Fig. S13**.

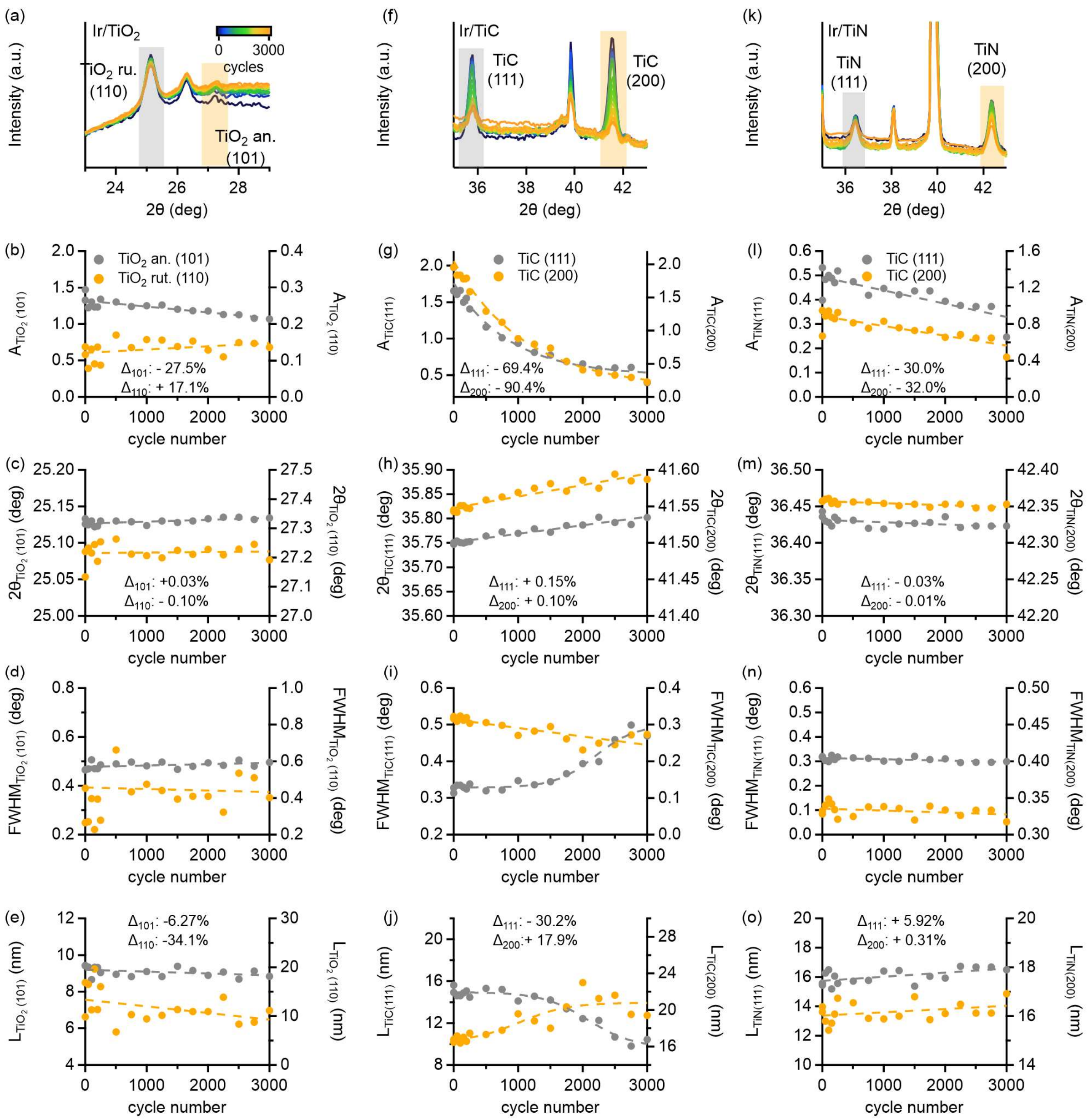

Fig. S13. Time-resolved WAXS pattern recorded in *operando* conditions and limited to the 2θ-range defined in **Fig. S12**, and corresponding time-resolved evolution of peak area (A), position (2θ), full-width-at-half-maximum (FWHM), and average crystallite size (L), for (a-e) Ir/TiO_2, (f-j) Ir/TiC, and (k-o) Ir/TiN. Note: dashed lines are a guide for the eye only.

S5. XAS analysis: post-mortem after operando

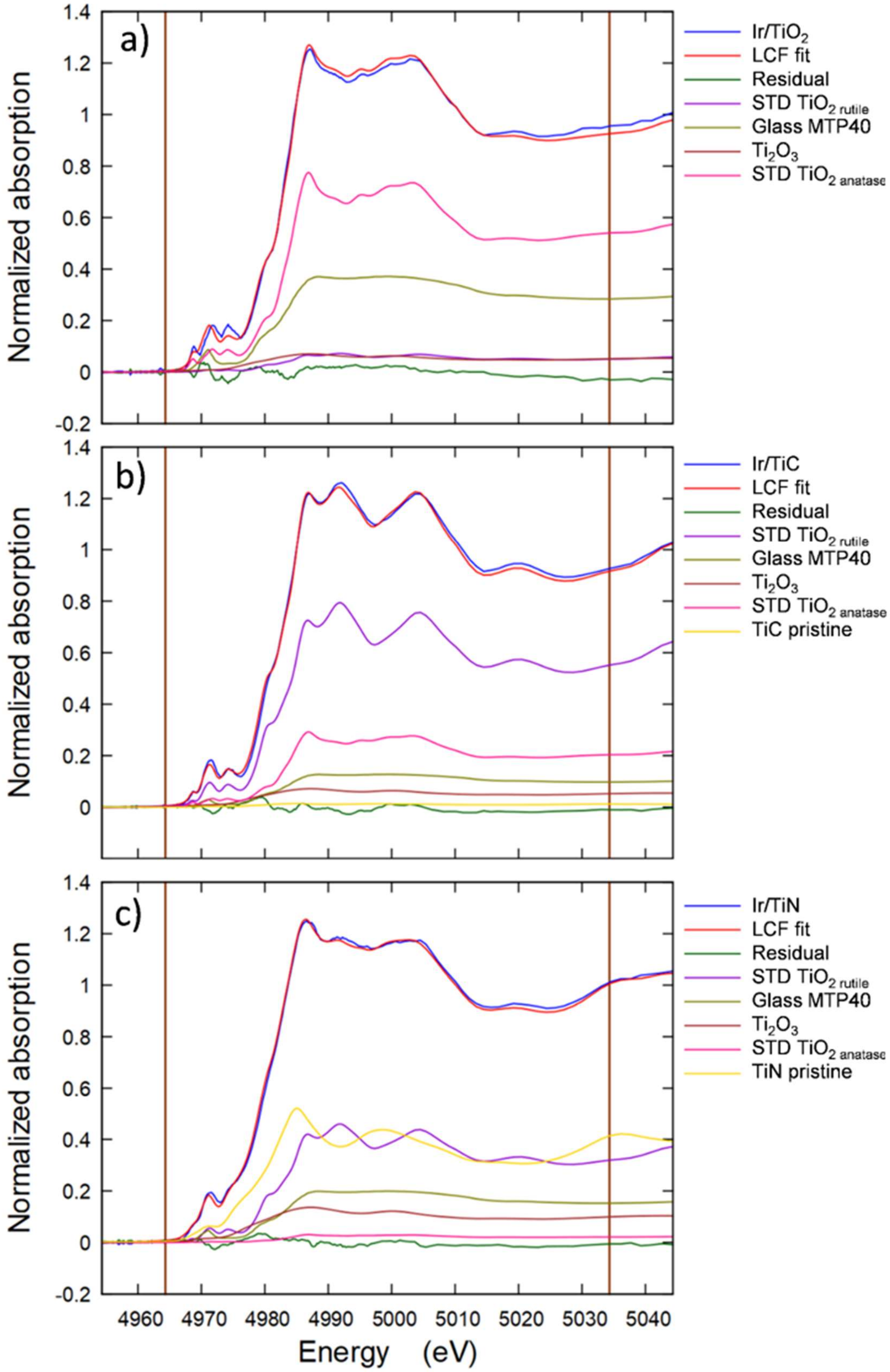

Fig. S14. Fits (red line) obtained through linear combination fitting analyses on the normalized XANES spectra of the *post-mortem samples* (blue lines), plotted with the weighted components and the residual curve (green line).
Note: STD TiO_2 anatase and rutile [24]. Glass MTP40 (MgO–TiO_2–P_2O_5) is a titanium phosphate-based Ti^{4+} reference material [25]. The estimated uncertainty of the LCF phase contributions is approximately ±5%, while smaller contributions should be interpreted qualitatively.

Table S4 Results of the linear combination fitting analyses conducted on the normalized Ti K-edge spectra.

Sample	Ti metal %	Ti_2O_3 %	TiN %	TiC %	Ti glass* %	TiO_2 (anatase) %	TiO_2 (rutile) %	Sum
**Pristine samples:**								
**TiO_2**	-	-	-	-	-	100	-	100
**Post-mortem samples:**								
**Ir-TiO_2**	-	5 (1)	-	-	32 (3)	57 (4)	6 (3)	100
**Ir-TiC**	-	5 (1)	-	1 (1)	11 (2)	22 (2)	61 (6)	100
**Ir-TiN**	-	11 (2)	30 (2)	-	12 (2)	11 (2)	35 (5)	100

*Glass MTP40 ($MgO–TiO_2–P_2O_5$) is a titanium phosphate-based Ti^{4+} reference material [25].

S6. EDX analysis: post-mortem after operando measurements

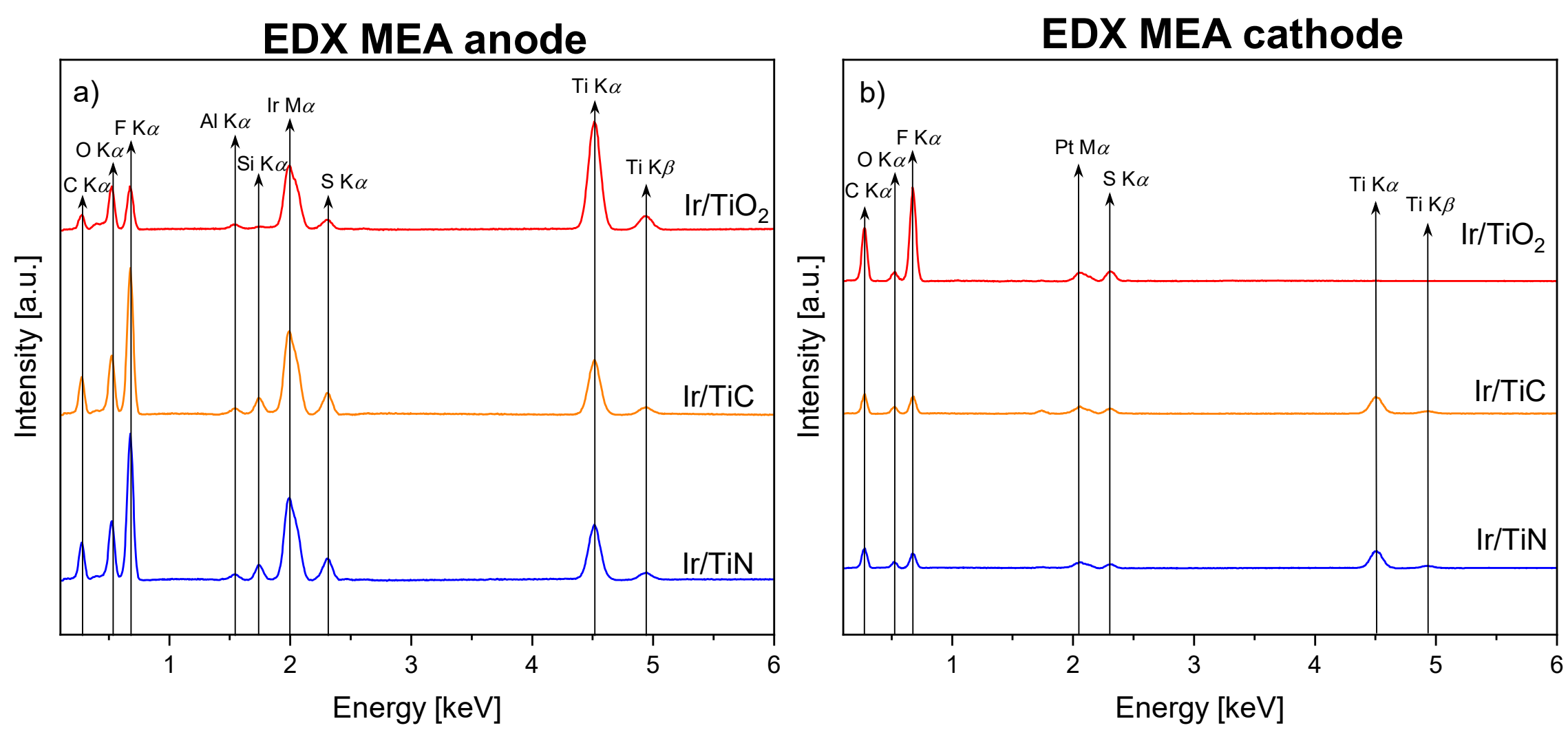

Fig. S15. EDX spectra of (a) the anode and (b) the cathode of the MEAs after *operando* experiments.

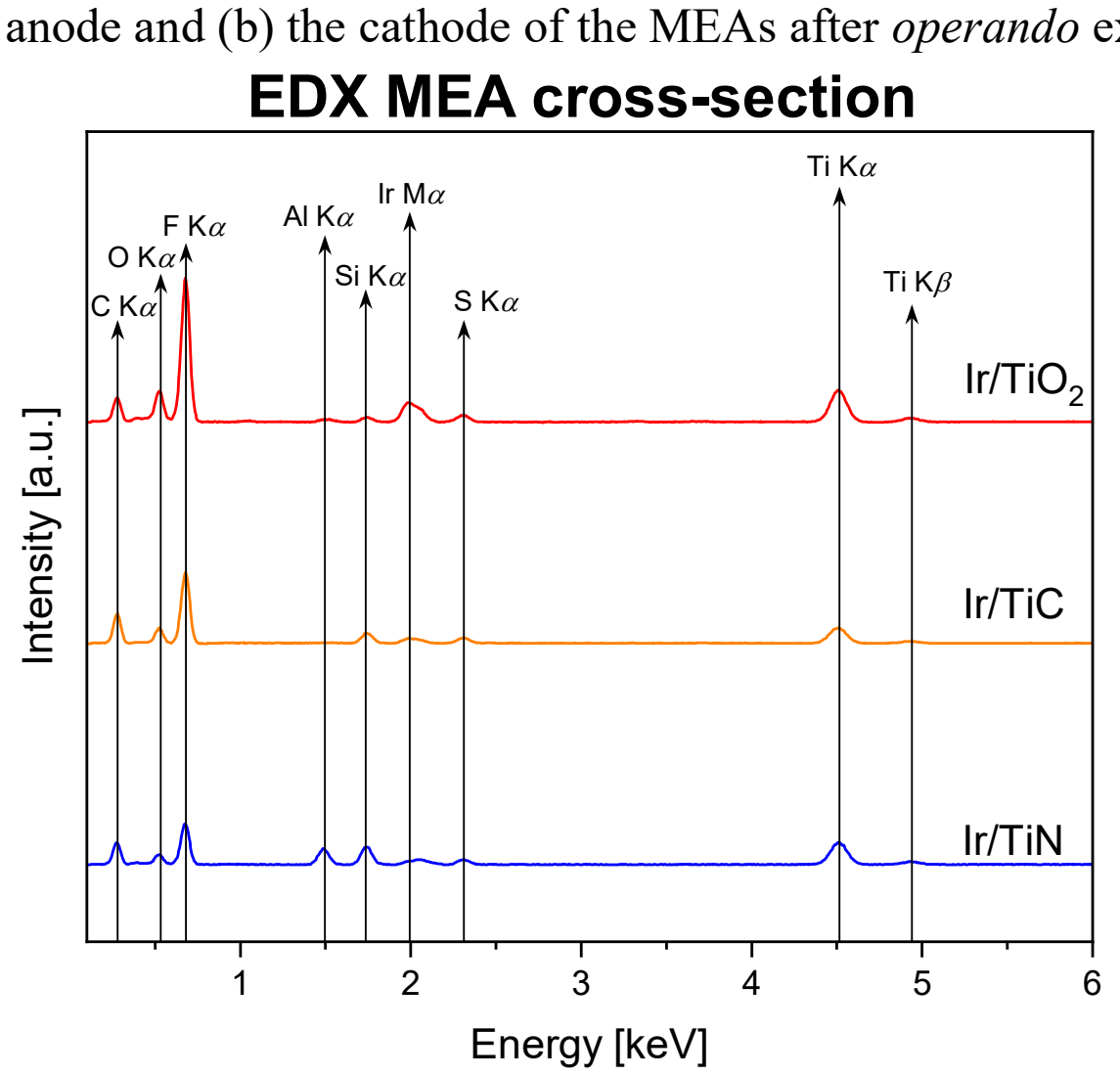

Fig. S16. EDX spectra of the MEA cross-section after *operando* experiments.

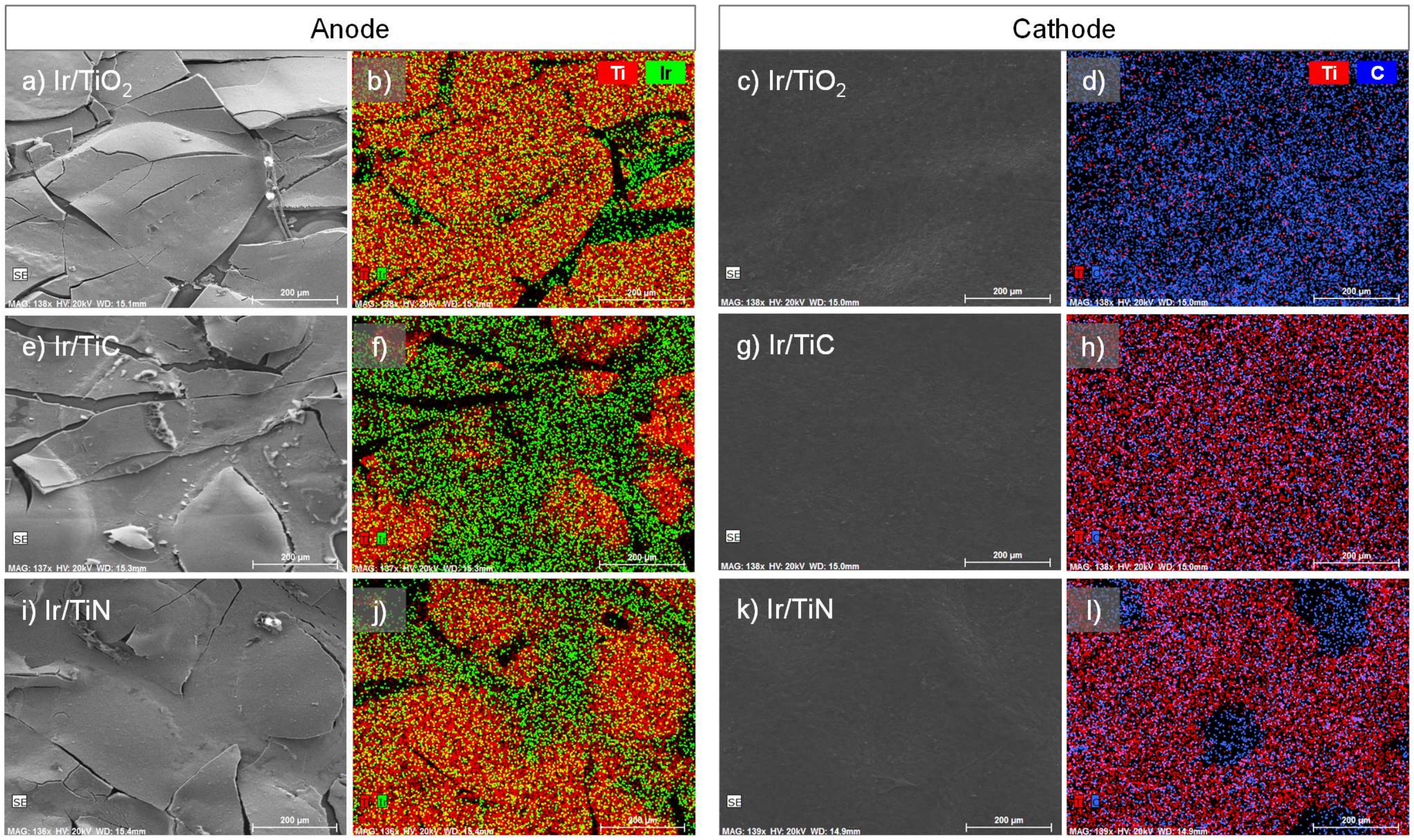

Fig. S17. SEM micrographs and their corresponding EDX mapping for the anode (left) and cathode (right) of the MEA for (a-d) Ir/TiO_2, (e-h) Ir/TiC, and (i-l) Ir/TiN after *operando* experiments. Color code: red (titanium), green (iridium), and blue (carbon).

S7. ICP-OES analysis: operando study

In the operando experiments (**Table S5**), Ti and Ir concentrations were below detection limits, whereas sulfur species were detected in all cases, with concentrations approximately twice as high for Ir/TiC and Ir/TiN-supported MEAs compared to Ir/TiO_2. This trend may indicate more pronounced ionomer degradation or transport of sulfur-containing degradation products in systems employing unstable supports, consistent with the hypothesis of support–ionomer interactions contributing to performance decay.

Table S5. Concentrations of Ti, Ir, and S ions measured in the anode-side effluent water after the accelerated stress tests in the water electrolyzer cell.

	**Samples**	**Ti (mg/L)**	**Ir (mg/L)**	**S (mg/L)**
1	Ir/TiO_2-$AST_{0\text{-}2V}$	<0.1	<0.02	0.516
2	Ir/TiO_2-$AST_{0\text{-}2V}$	<0.1	<0.02	0.534
3	Ir/TiC -1	<0.1	<0.02	0.833
4	Ir/TiC -2	<0.1	<0.02	0.908
5	Ir/TiN -1	<0.1	<0.02	0.979
6	Ir/TiN -2	<0.1	<0.02	0.989

Note: for each sample, two independent measurements of the anode effluent water were performed. Total volume of circulated H_2O is 1 L.

S8. DRT analysis of PEIS data: operando synchrotron measurements (0–2 V AST protocol)

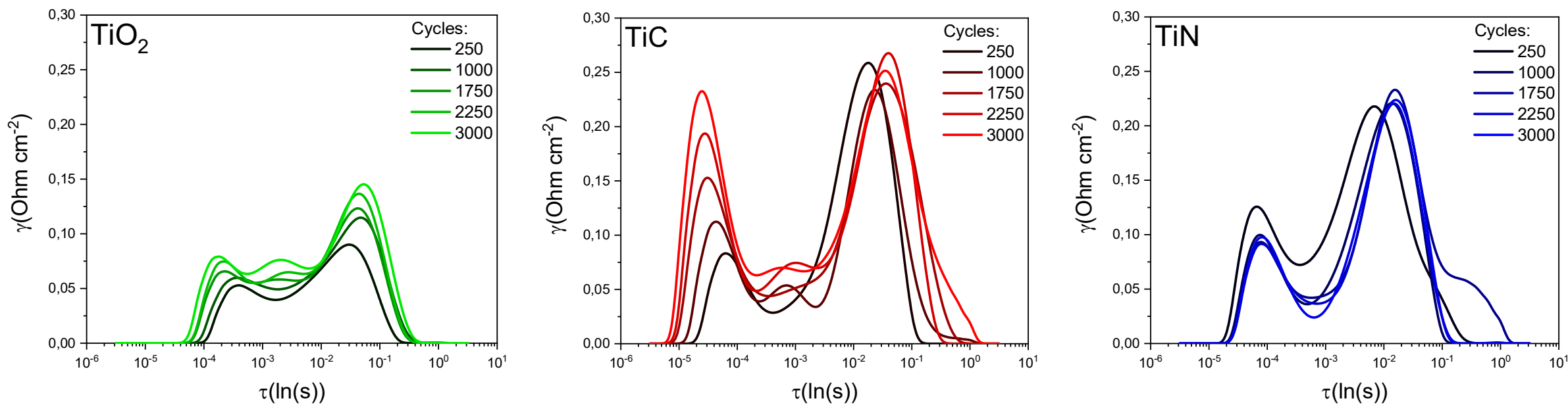

Fig. S18. DRT spectra of water electrolyzers with different anode catalysts, measured *operando* at 1.5 V. Regularization parameter λ=0.1.

LABORATORY MEASUREMENTS

S9. Electrochemical analysis: laboratory measurements

a) $AST_{0\text{-}2V}$ (1st) b) $AST_{0\text{-}2V}$ (2nd) c) $AST_{0\text{-}1.7V}$

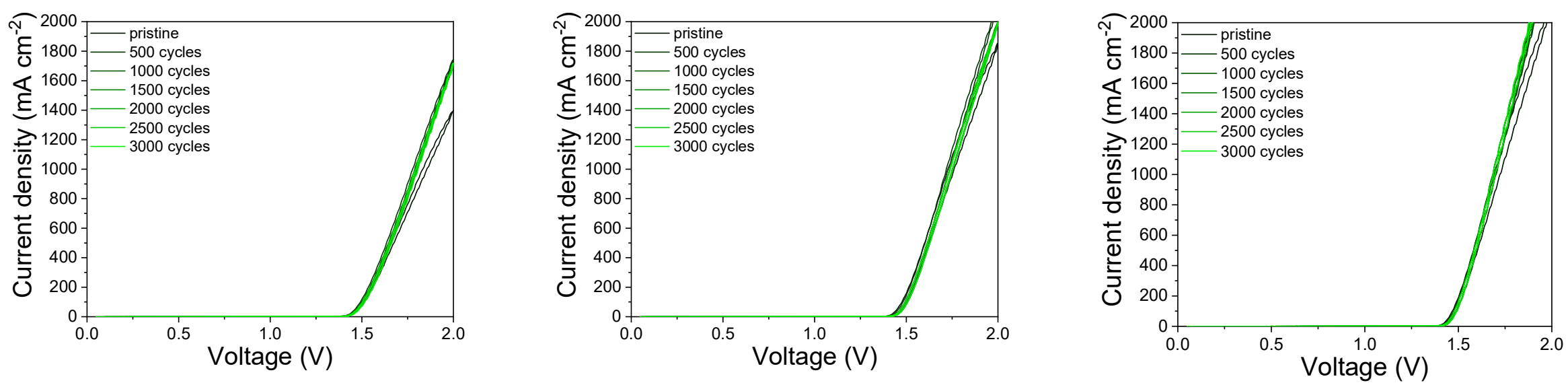

Fig. S19. Cyclic voltammograms of the Ir/TiO_2 MEA obtained during laboratory durability tests: (a) the first $AST_{0–2\ V}$ experiment, (b) the second $AST_{0–2\ V}$ experiment, and (c) the $AST_{0–1.7\ V}$ experiment.

a) $AST_{0\text{-}2V}$ (1st) b) $AST_{0\text{-}2V}$ (2nd) c) $AST_{0\text{-}1.7V}$

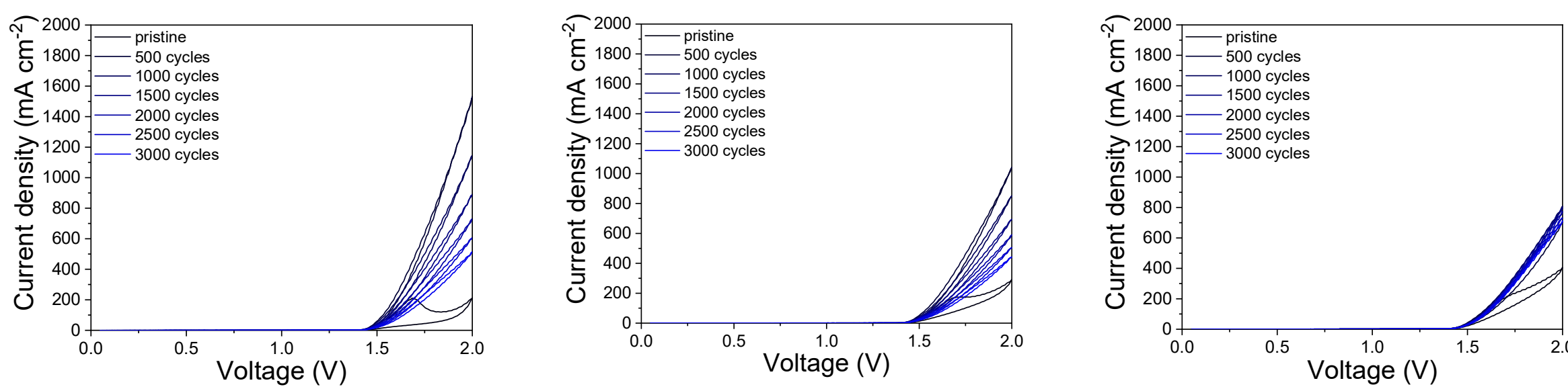

Fig. S20. Cyclic voltammograms of the Ir/TiN MEA obtained during laboratory durability tests: (a) the first $AST_{0–2\ V}$ experiment, (b) the second $AST_{0–2\ V}$ experiment, and (c) the $AST_{0–1.7\ V}$ experiment.

a) $AST_{0\text{-}2V}$ (1st) b) $AST_{0\text{-}2V}$ (2nd) c) $AST_{0\text{-}1.7V}$

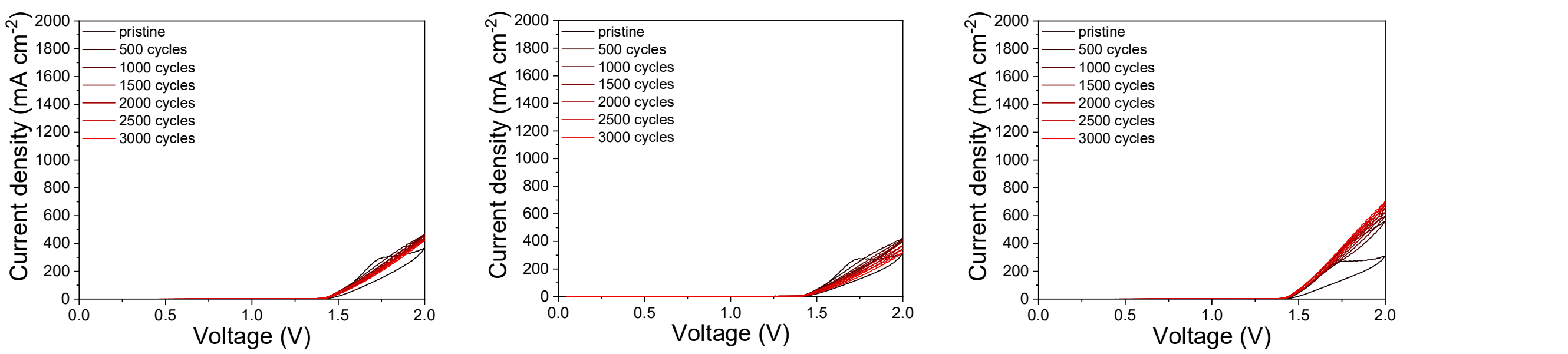

d) $AST_{0.4A/cm2}$

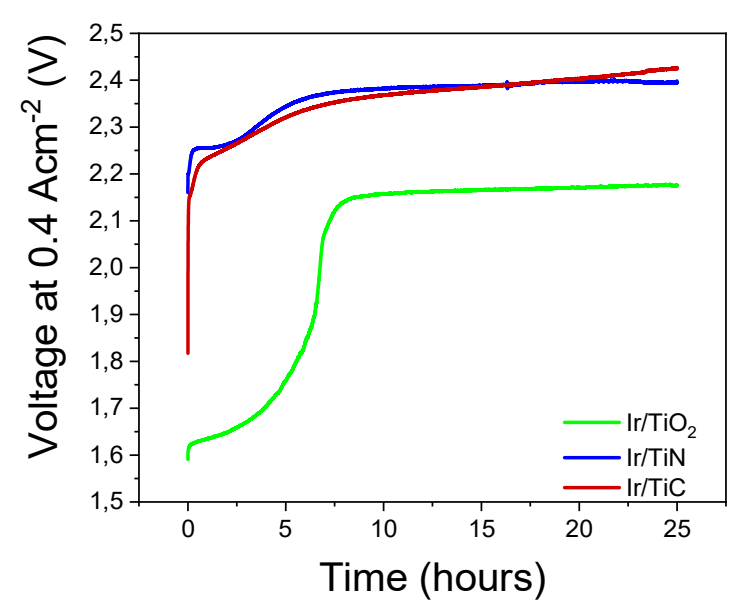

Fig. S21. Cyclic voltammograms of the Ir/TiC MEA obtained during laboratory durability tests: (a) the first $AST_{0–2\ V}$ experiment, (b) the second $AST_{0–2\ V}$ experiment, (c) the $AST_{0–1.7\ V}$ experiment, and (d) the 0.4 A cm^{-2} constant-current experiment.

Reproducibility $AST_{0\text{-}2V}$

Ir/TiO2 Ir/TiN Ir/TiC

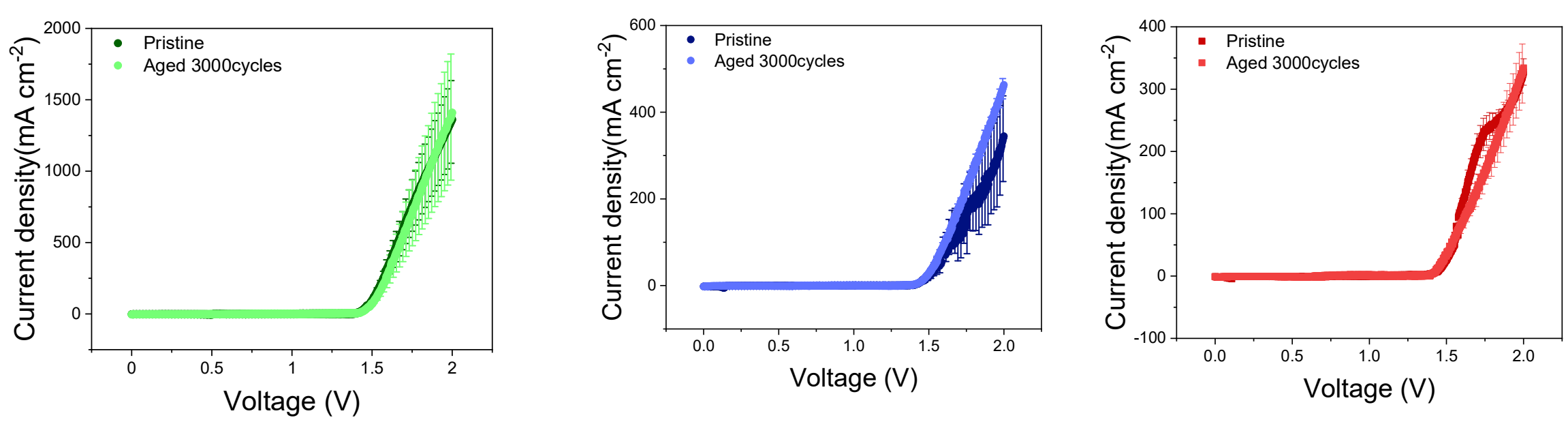

a) b) c)

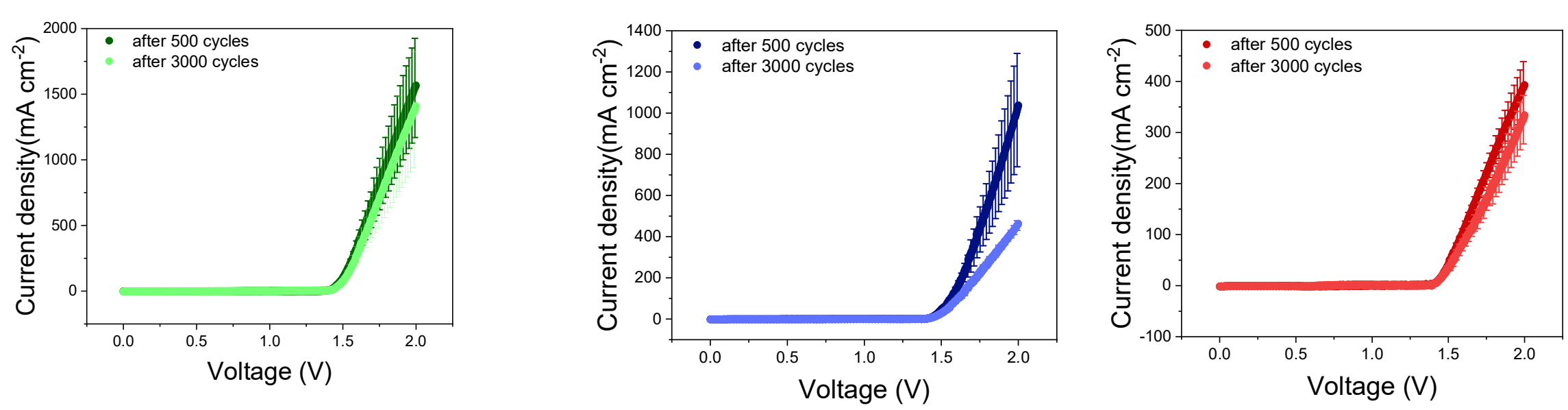

d) e) f)

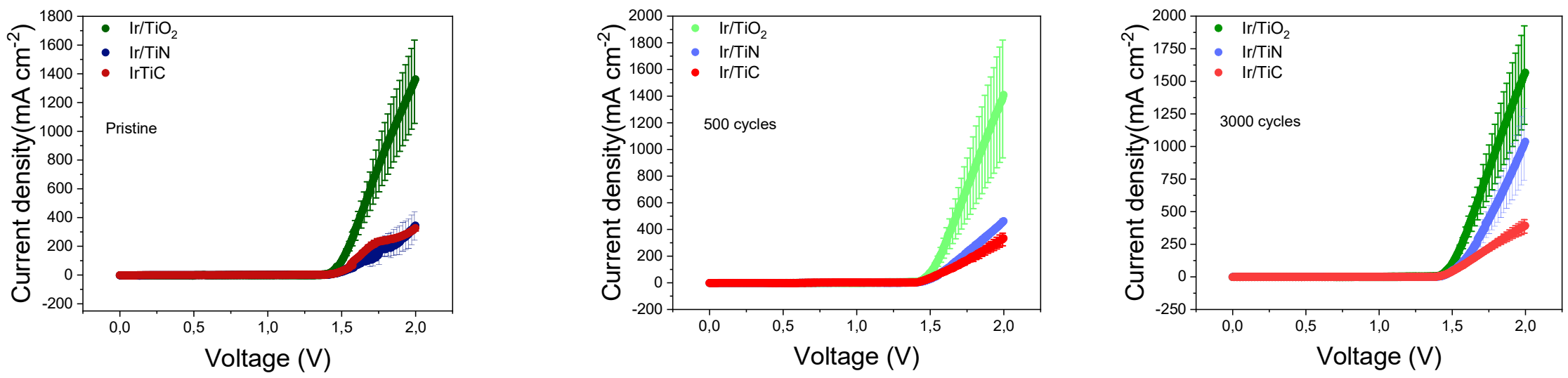

g) h) i)

Fig. S22. Reproducibility of the cyclic voltammograms recorded for (a) Ir/TiO_2, (b) Ir/TiN, and (c) Ir/TiC MEAs before and after 3000 cycles of the $AST_{0–2\ V}$ protocol (a-c), after 500 and 3000 cycles (d-f), and pristine combined (g), after 500 cycles (h), after 3000 cycles (i). The statistical evaluation was based on three independently prepared MEAs, including one MEA tested during the operando synchrotron experiment (**Fig. 1**) and two MEAs tested under laboratory conditions (**panels a and b of Figs. S19-22a-b**). The laboratory measurements were performed using an independently synthesized catalyst batch.

S10. EDX analysis: post-mortem after laboratory measurements

The laboratory durability tests demonstrate trends consistent with those observed in the operando experiments (**Fig. 6**). Similar behavior was observed for all investigated catalysts (Ir/TiO_2, Ir/TiN, and Ir/TiC) after the AST0–2V protocol (**Figs. S23–S25, e–h**), confirming the reproducibility of the degradation phenomena. It should be noted that the signal detected on the cathode side and assigned to Ir is also present in pristine MEAs. This contribution originates from the Pt catalyst layer, as Pt and Ir cannot be reliably distinguished by EDX due to the overlap of their characteristic X-ray lines.

For Ir/TiO_2 (**Fig. S23**), the elemental maps indicate good structural stability after all durability protocols, with no significant redistribution of Ti observed. In contrast, the Ir/TiN catalyst (**Fig. S24**) exhibits Ti migration after $AST_{0–2V}$ (**Fig. S24f**) and $AST_{0.4A}$ (**Fig. S24n**). The $AST_{0–1.7V}$ sample (**Fig. S24j**) does not allow a clear assessment because the analyzed region appears to be shifted toward the cathode side of the MEA. For Ir/TiC (**Fig. S25**), Ti migration is observed after all durability protocols, namely $AST_{0–2V}$ (**Fig. S25f**), $AST_{0–1.7V}$ (**Fig. S25j**), and $AST_{0.4A}$ (**Fig. S25n**), indicating a lower stability of the TiC support under the investigated operating conditions.

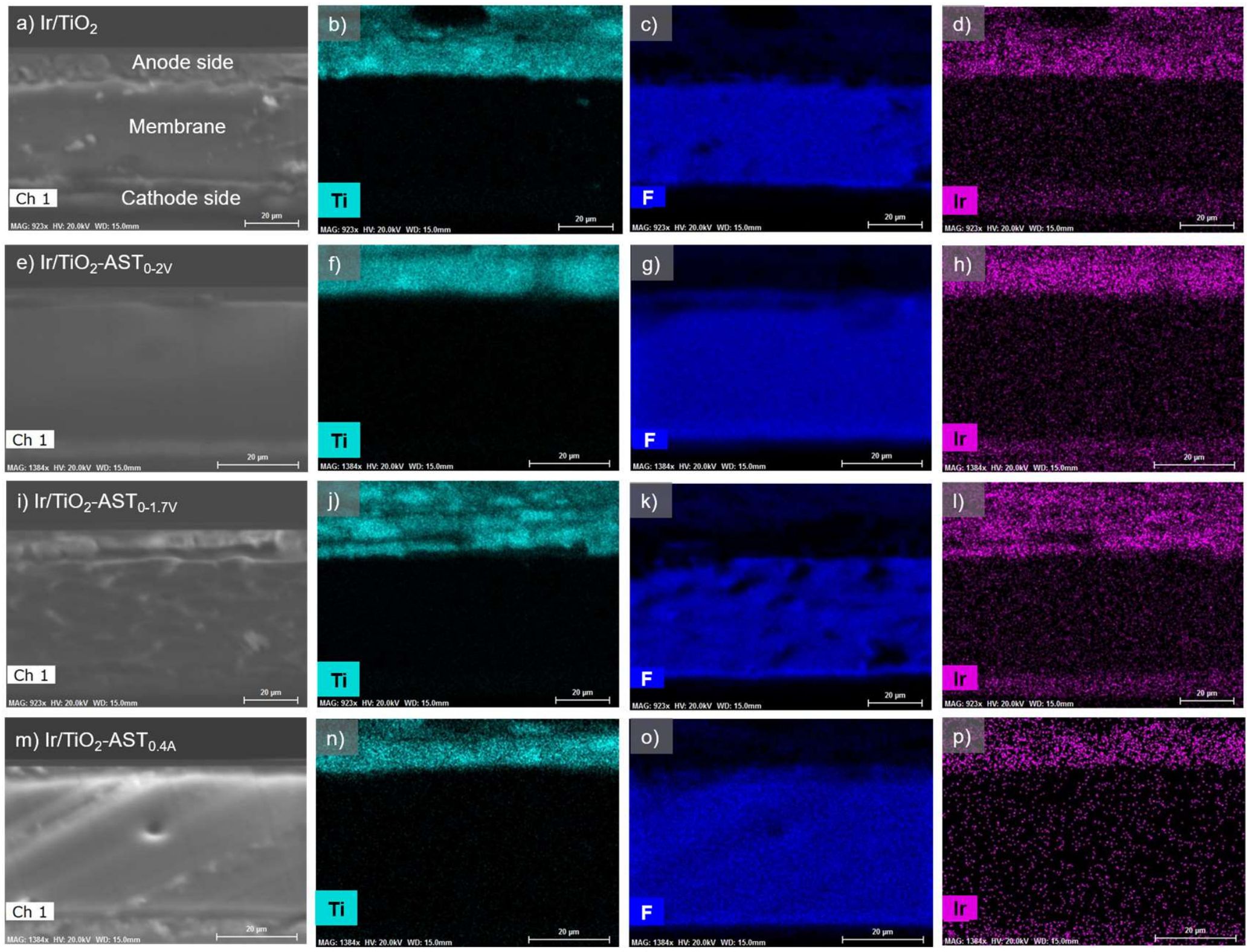

Fig. S23. Cross-sectional SEM images (a, e, i, m) and corresponding EDX elemental maps (b–d, f–h, j–l, n–p) of MEAs containing the Ir/TiO_2 catalyst before testing (a–d) and after laboratory durability tests performed under $AST_{0\text{–}2V}$ (e–h), $AST_{0\text{–}1.7V}$ (i–l), and $AST_{0.4A}$ (m–p) protocols. Color code: cyan (Ti), dark blue (F), and pink (Ir).

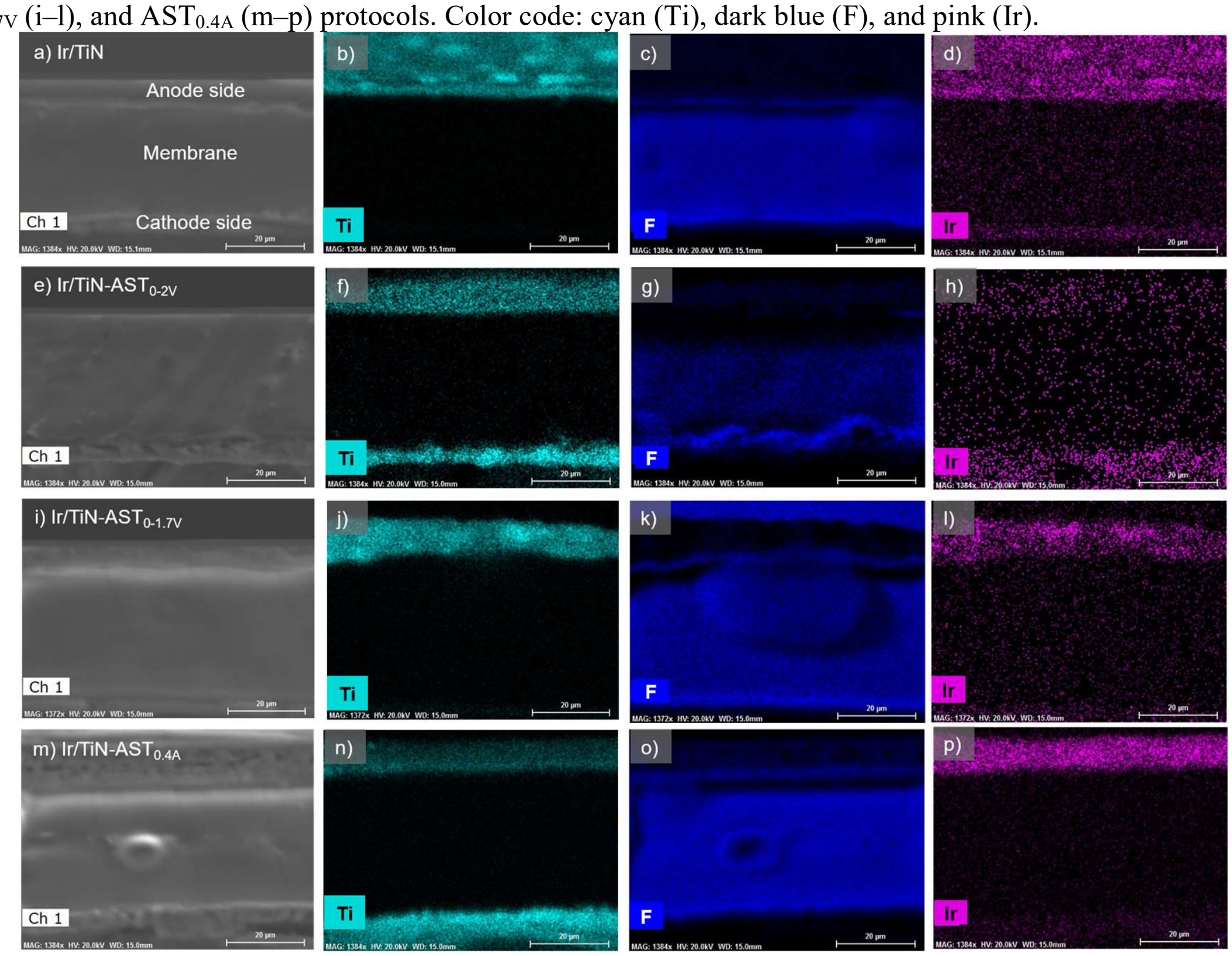

Fig. S24. Cross-sectional SEM images (a, e, i, m) and corresponding EDX elemental maps (b–d, f–h, j–l, n–p) of MEAs containing the Ir/TiN catalyst before testing (a–d) and after laboratory durability tests performed under $AST_{0\text{–}2V}$ (e–h), $AST_{0\text{–}1.7V}$ (i–l), and $AST_{0.4A}$ (m–p) protocols. Color code: cyan (Ti), dark blue (F), and pink (Ir).

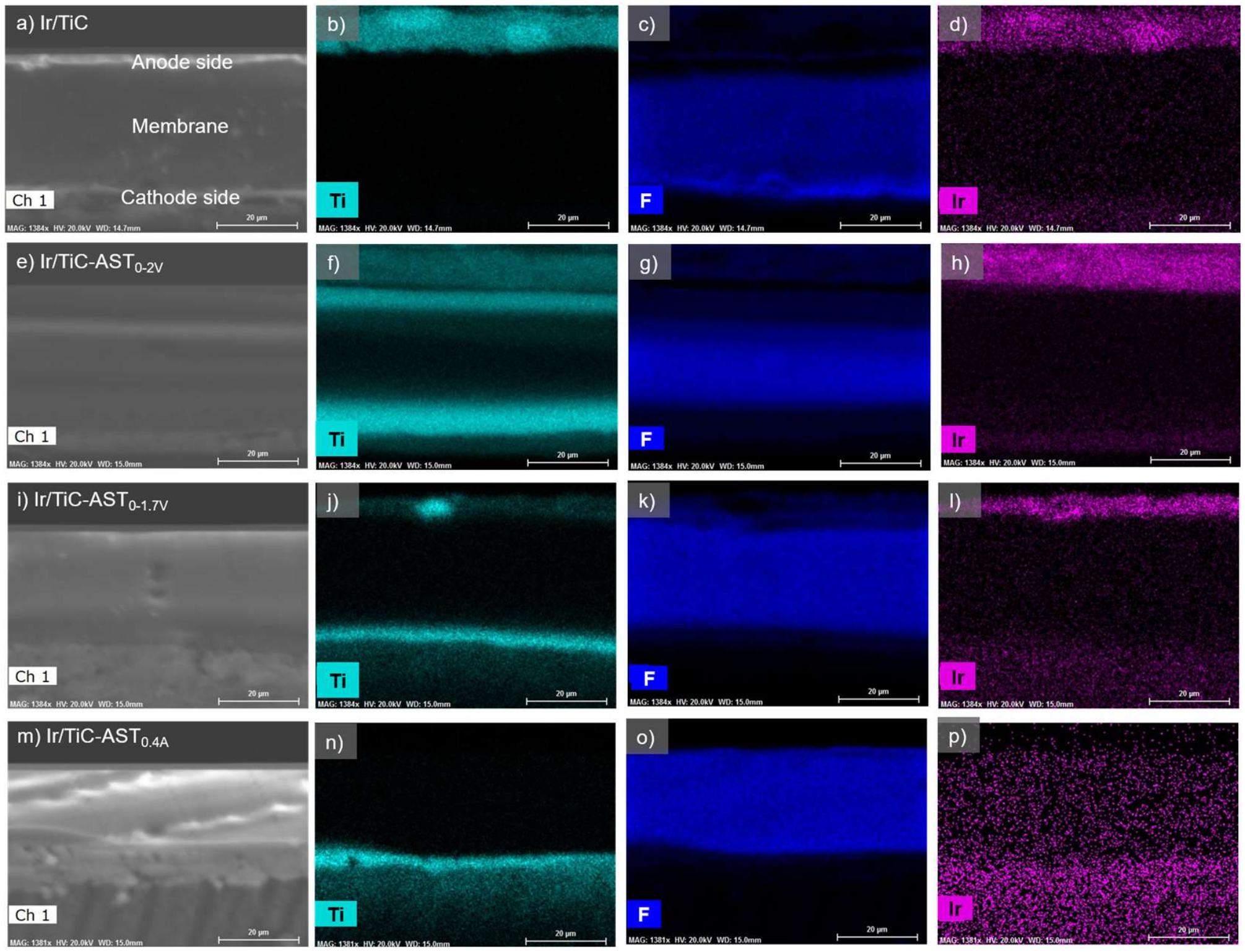

Fig. S25. Cross-sectional SEM images (a, e, i, m) and corresponding EDX elemental maps (b–d, f–h, j–l, n–p) of MEAs containing the Ir/TiC catalyst before testing (a–d) and after laboratory durability tests performed under $AST_{0–2V}$ (e–h), $AST_{0–1.7V}$ (i–l), and $AST_{0.4A}$ (m–p) protocols. Color code: cyan (Ti), dark blue (F), and pink (Ir).

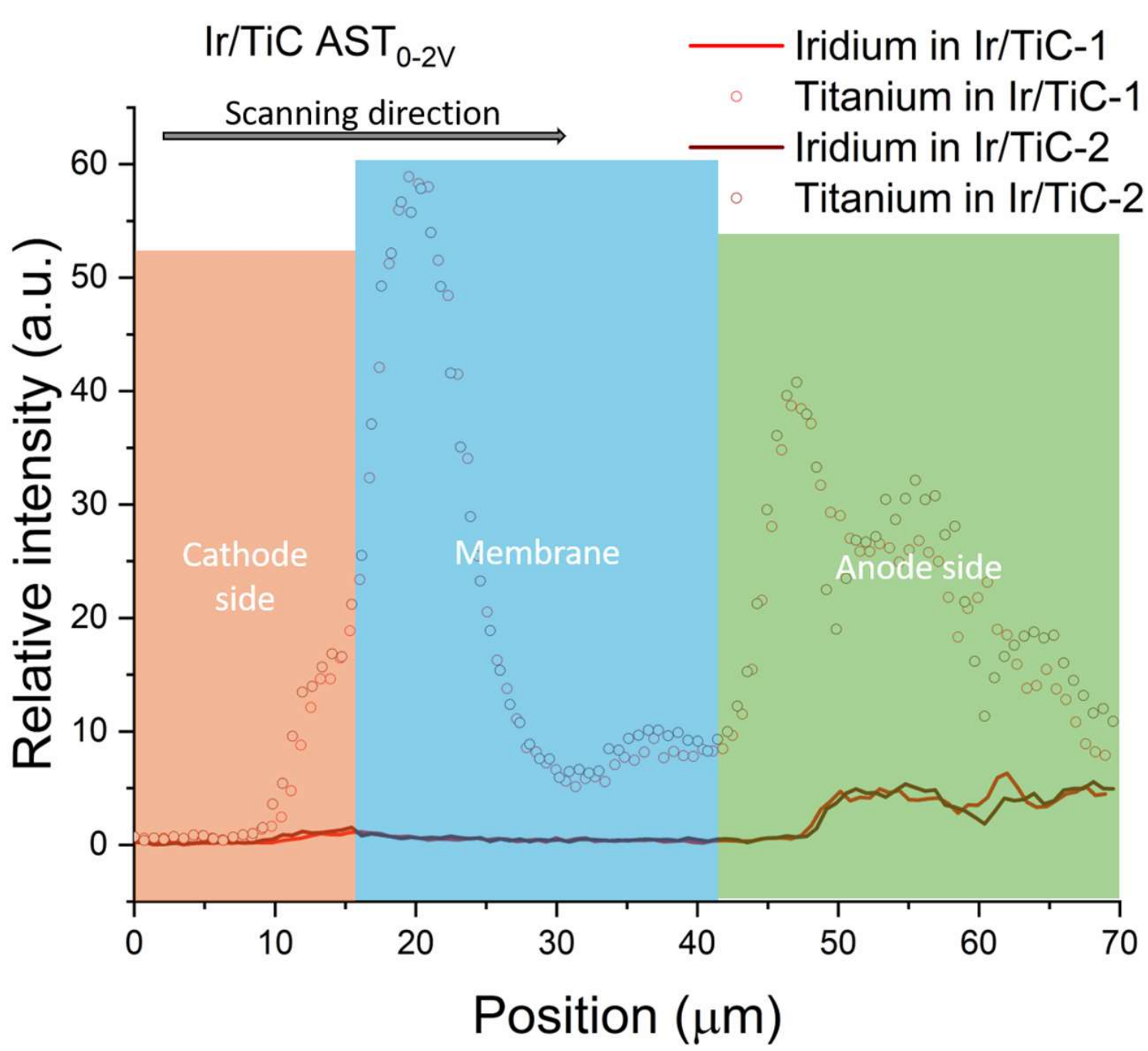

Fig. S26. Cross-sectional EDX line-scan profiles of Ir and Ti across two independently prepared Ir/TiC MEAs after the AST_{0-2V} protocol. The shaded regions indicate the approximate positions of the cathode, membrane, and anode.

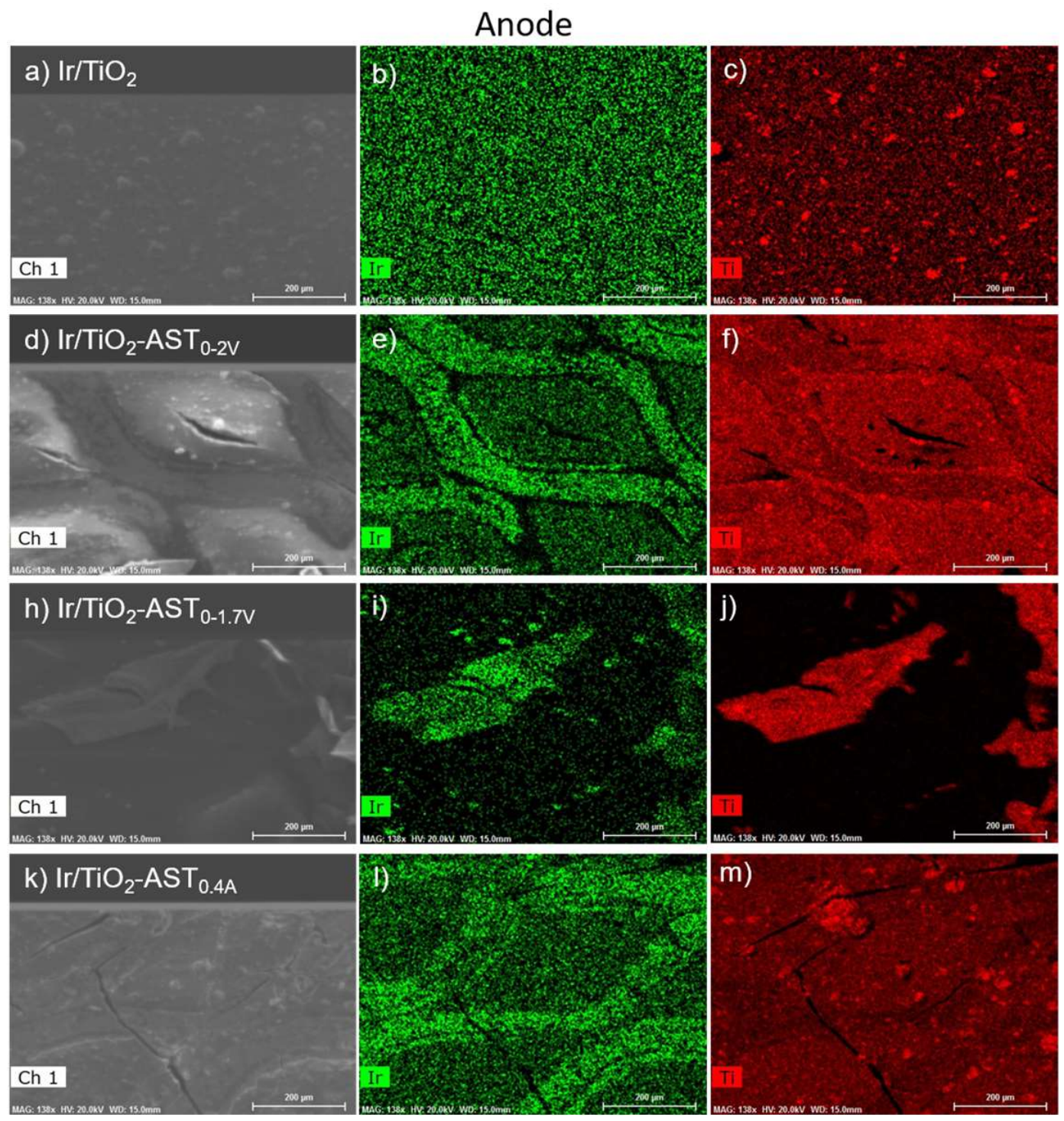

Fig. S27. Planar SEM images (a, d, h, k) and their corresponding elemental maps (b–c, e–f, i–j, l–m) of the anode catalyst layer in the Ir/TiO_2 MEA before testing (a–c) and after laboratory durability tests performed under $AST_{0–2V}$ (d–f), $AST_{0–1.7V}$ (h–j), and $AST_{0.4A}$ (k–m) protocols. Color code: green (iridium) and red (titanium).

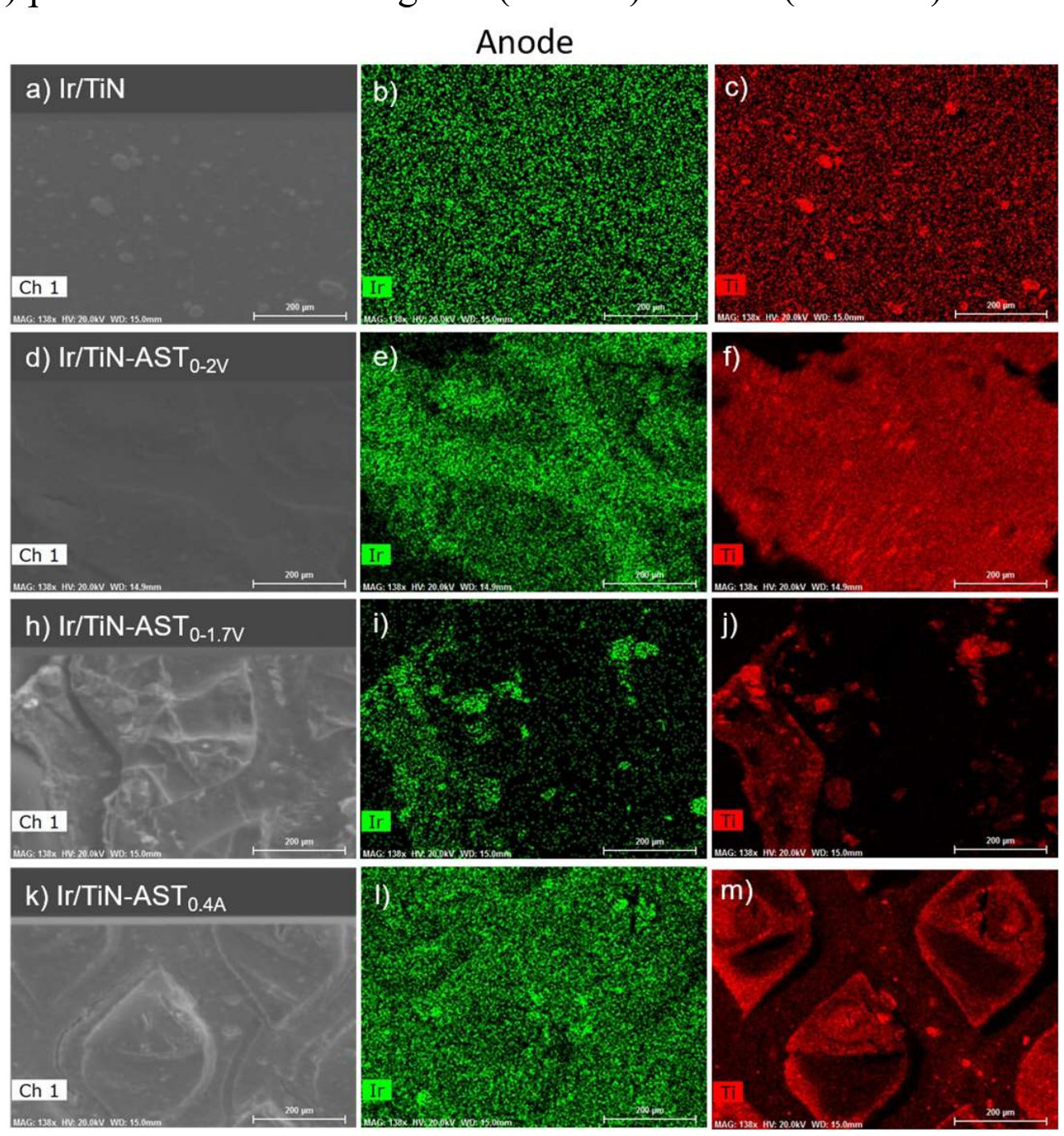

Fig. S28. Planar SEM images (a, d, h, k) and their corresponding elemental maps (b–c, e–f, i–j, l–m) of the anode catalyst layer in the Ir/TiN MEA before testing (a–c) and after laboratory durability tests performed under $AST_{0–2V}$ (d–f), $AST_{0–1.7V}$ (h–j), and $AST_{0.4A}$ (k–m) protocols. Color code: green (iridium) and red (titanium).

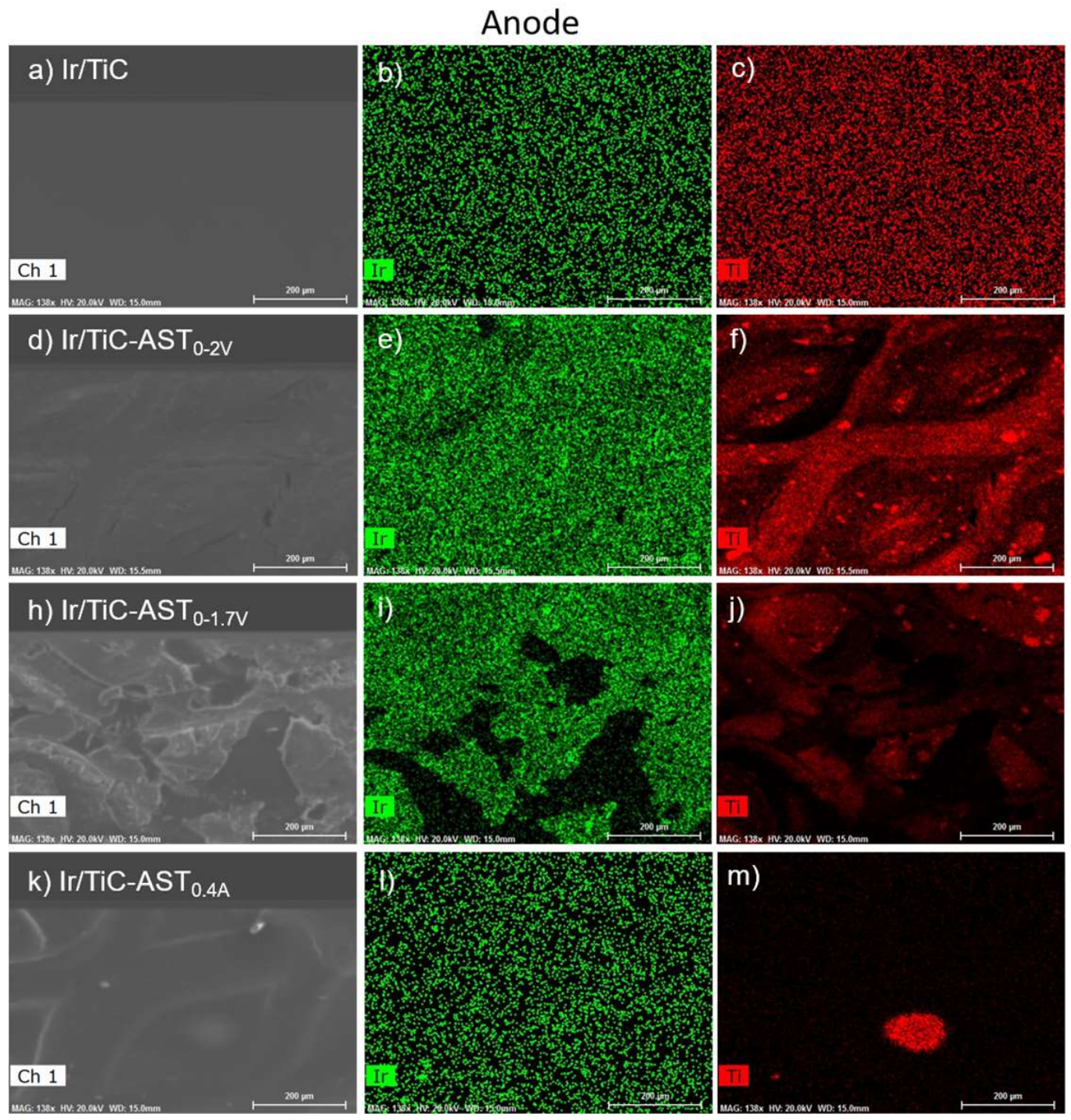

Fig. S29. Planar SEM images (a, d, h, k) and their corresponding elemental maps (b–c, e–f, i–j, l–m) of the anode catalyst layer in the Ir/TiC MEA before testing (a–c) and after laboratory durability tests performed under AST_{0-2V} (d–f), $AST_{0-1.7V}$ (h–j), and $AST_{0.4A}$ (k–m) protocols. Color code: green (iridium) and red (titanium).

S11. HR-TEM analysis: post-mortem after laboratory measurements

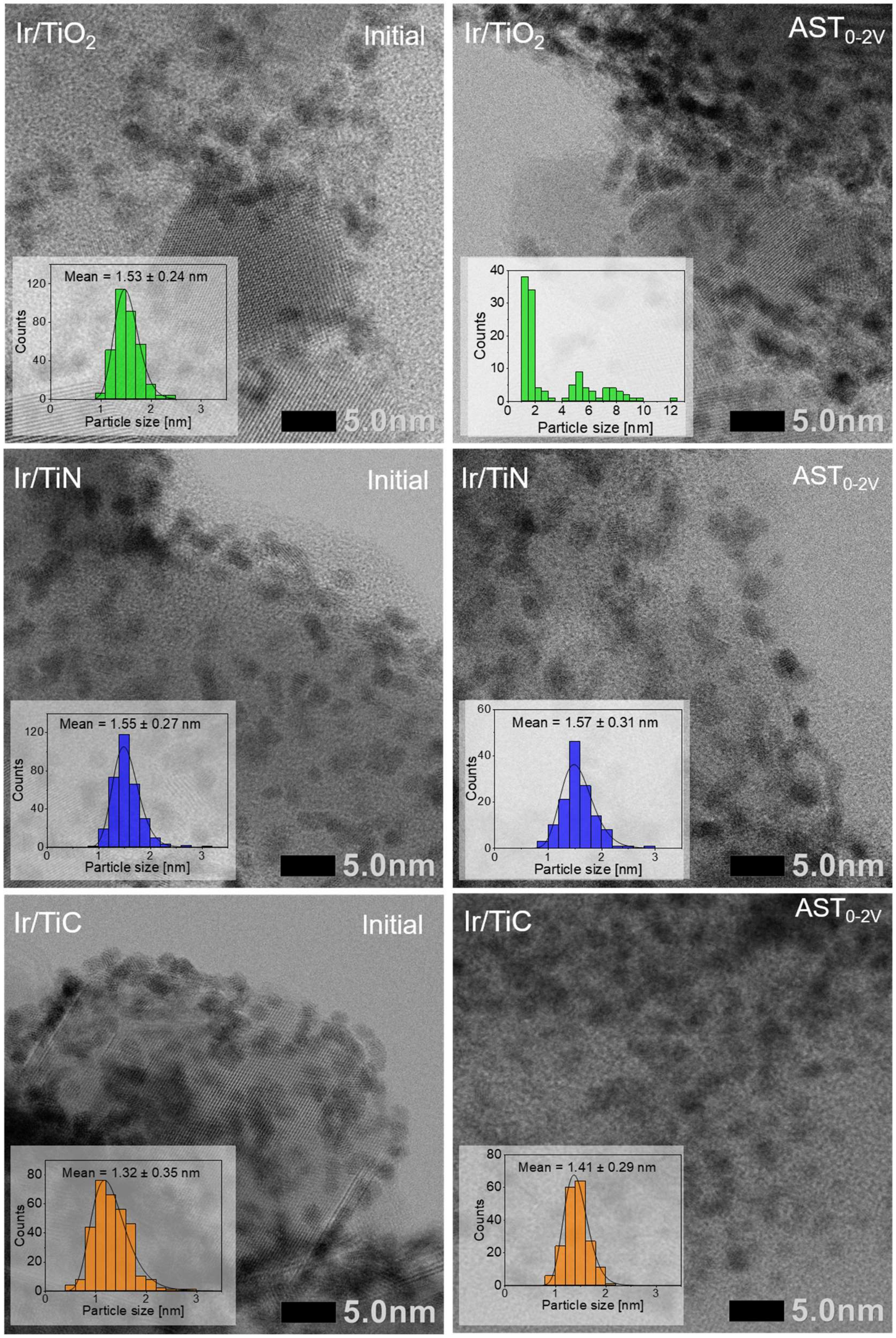

Fig. S30. HR-TEM micrographs of initial newly synthesized nanoparticles and nanoparticles from the anode of Ir/TiO_2, Ir/TiN, and Ir/TiC MEAs after laboratory durability tests at 0-2V, and Ir particle size distribution (insets).

S12. WAXS analysis: post-mortem after laboratory measurements

Results from WAXS (**Fig. S31**) are characterized by a similar outlook: TiO_2 rutile (110) and TiO_2 anatase (101) peaks can be clearly detected in pristine CCM (25° and 27° respectively, **Fig. 31a**), but barely in the aged CCM. This lack might be reasonably attributed to the occurrence of delamination. TiC (111) and (200) diffraction peaks (35.8° and 41.5°, respectively; **Fig. S31b**) are visible in both powder and pristine CCM, but they are almost vanished from aged CCM, in agreement with what was observed by SAXS, supporting the picture of strong dissolution. Finally, TiN (111) and (200) diffraction peaks (36.5° and 42.3° respectively, **Fig. S31c**), can be observed to have almost vanished from aged CCM, too, but their reduced intensity in pristine CCM makes it reasonable to support the picture of partial dissolution.

Last but not least, being the region around 40° influenced by the diffraction peaks of multiple elements, such as: Nafion (40°), Pt (111) (39.76°), Ir (111) (40.66°), and IrO_X (200) (40.06°), obtained results from peak deconvolution (highlighting an average crystallite size of: 2.70, 1.93, and 4.65 nm for Ir/TiO_2, Ir/TiC, and Ir/TiN in pristine conditions, and an average crystallite size of: 3.63, 1.78, and 4.06 nm for Ir/TiO_2, Ir/TiC, and Ir/TiN in aged CCMs), are hardly connectable with results obtained by SAXS.

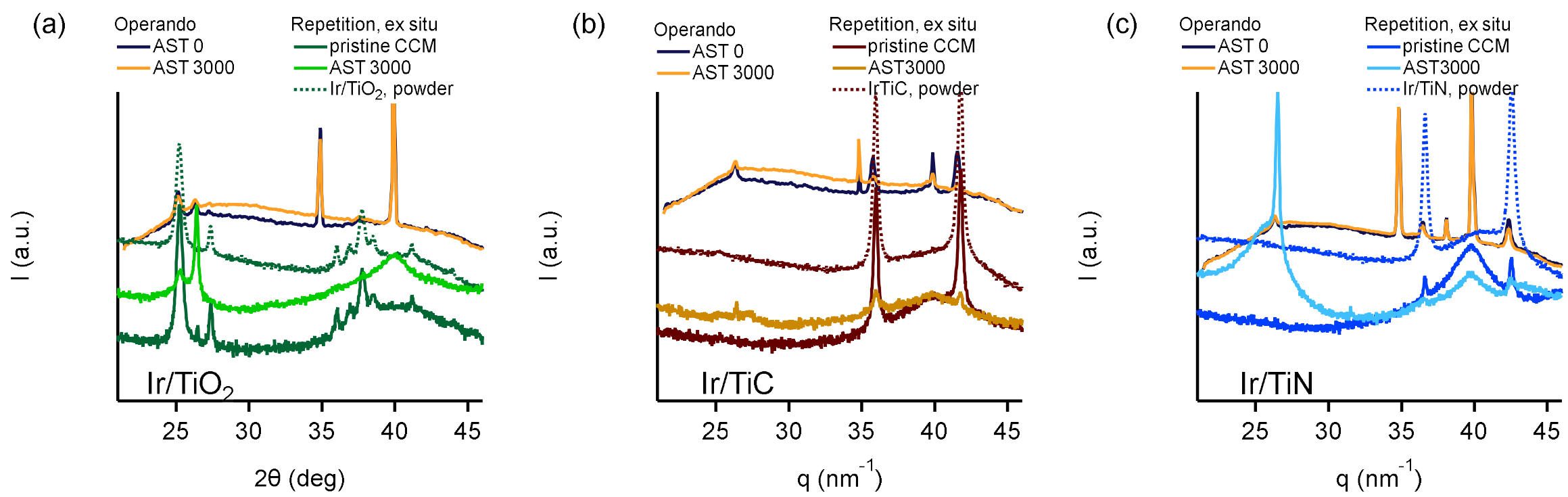

Fig. S31. Comparison of WAXS patterns from operando and ex situ analysis for the three formulations: (a) TiO_2, (b) TiC, and (c) TiN.

Overall, the *ex situ* WAXS measurements reproduce the main degradation trends observed *operando* despite unavoidable differences arising from CCM disassembly and partial catalyst layer delamination. Ir/TiN showed good agreement between the *operando* and *ex situ* measurements for both the support and Ir nanoparticles. For Ir/TiC, good agreement was observed for the support-related changes, whereas the Ir nanoparticle evolution was less reproducible. Conversely, for Ir/TiO_2, the Ir nanoparticle behavior showed good agreement, while the support-related changes were less consistently reproduced.

S13. ICP-MS analysis: post-mortem after laboratory measurements

To improve the sensitivity of the ICP-MS analysis, the total volume of circulated anode water used in the in-house experiments was reduced from 1 L (operando experiments) to 0.4 L. This modification increased the concentration of dissolved species that were previously below the detection limit in the operando measurements.

The in-house ICP-MS measurements (**Table S6**) allowed detection of low concentrations of Ir species in both circulated anode water and cathode-collected water. Although the overall concentrations were very low, the Ir/TiO_2 system consistently exhibited higher Ir concentrations under all degradation protocols than Ir/TiN and Ir/TiC. Although this trend may be consistent with the SAXS observations, which suggested more pronounced Ir nanoparticle restructuring in the Ir/TiO_2 system

than in Ir/TiN and Ir/TiC, the very low measured concentrations only allow this interpretation to be considered tentative.

Ti concentrations in the circulated anode water remained below the detection limit for all systems. Nevertheless, small amounts of Ti were detected in the cathode-collected water after AST, indicating migration of Ti species through the MEA during operation. Although higher Ti concentrations were expected for the Ir/TiN and Ir/TiC systems, the observed trend is still consistent with the planar and cross-sectional EDX analyses, which revealed gradual transport of Ti and Ir species from the anode side through the membrane toward the cathode. In particular, the cross-sectional EDX maps show accumulation of migrated species within the membrane region, suggesting partial retention of dissolved species inside the MEA rather than complete transport into the collected water.

Sulfur-containing species were detected in both anode and cathode water for all investigated systems. In general, the cathode-collected water exhibited higher sulfur concentrations than the circulated anode water, suggesting transport of sulfur-containing degradation products through the membrane during operation. The Ir/TiC and Ir/TiN systems showed trends consistent with increased ionomer degradation and transport-related changes observed in the DRT and EDX analyses.

The milder 0–1.7 V AST protocol generally resulted in slightly lower concentrations of dissolved species compared to the harsh 0–2 V protocol, indicating reduced degradation under less aggressive operating conditions.

Table S6. Concentrations of Ti, Ir, and S ions measured in the anode-side circulated water and cathode-gathered water after the accelerated stress tests in the water electrolyzer cell.

№	Samples	*Circulated Anode H_2O			№	**Produced Cathode H_2O		
		Ir (mg/L)	Ti (mg/L)	S (mg/L)		Ir (mg/L)	Ti (mg/L)	S (mg/L)
0	H_2O blank	<0.002	<0.001	0.217±0.003				
1***	$IrTiO_2$-$AST_{0\text{-}2V}$	0.021±0.005	<0.001	0.215±0.003	10****	0.046±0.015	0.006±0.003	0.428±0.003
2***	IrTiN-$AST_{0\text{-}2V}$	<0.002	<0.001	0.197±0.004	11****	0.003±0.001	0.005±0.001	0.332±0.029
3***	IrTiC-$AST_{0\text{-}2V}$	0.004±0.000	<0.001	0.200±0.004	12****	0.006±0.002	0.004±0.002	0.356±0.105
4	$IrTiO_2$-$AST_{0\text{-}1.7V}$	0.014±0.000	<0.001	0.207±0.003	13	0.032	0.004	0.422
5	IrTiN-$AST_{0\text{-}1.7V}$	<0.002	<0.001	0.210±0.003	14	0.004	0.004	0.303
6	IrTiC-$AST_{0\text{-}1.7V}$	0.003±0.000	<0.001	0.198±0.004	15	<0.002	0.002	0.251
7	$IrTiO_2$-$AST_{0.4A}$	0.011±0.000	<0.001	0.211±0.003	16	0.022	0.001	0.379
8	IrTiN-$AST_{0.4A}$	<0.002	<0.001	0.208±0.005	17	0.003	<0.001	0.406
9	IrTiC-$AST_{0.4A}$	0.003±0.000	<0.001	0.207±0.006	18	0.014	0.002	0.454

*Total volume of circulated anode water was 0.4 L.

** The analyzed volume of cathode-collected water was 10 mL. The reported ion concentrations were not normalized to the total volume of produced water.

*** Mean values were calculated from two independently tested samples, with three aliquots analyzed for each sample.

**** Mean values were calculated from two independently tested samples, with one aliquot analyzed for each sample.

S14. Fluoride ions: laboratory measurements (0–2 V AST protocol)

Fluoride concentration was determined potentiometrically using a fluoride ion-selective electrode HI 4010 (Hanna Instrument) according to EPA Method 9214 [26]. **Figure S32** shows the calibration curve together with the measurements of the water samples collected after the electrolysis test.

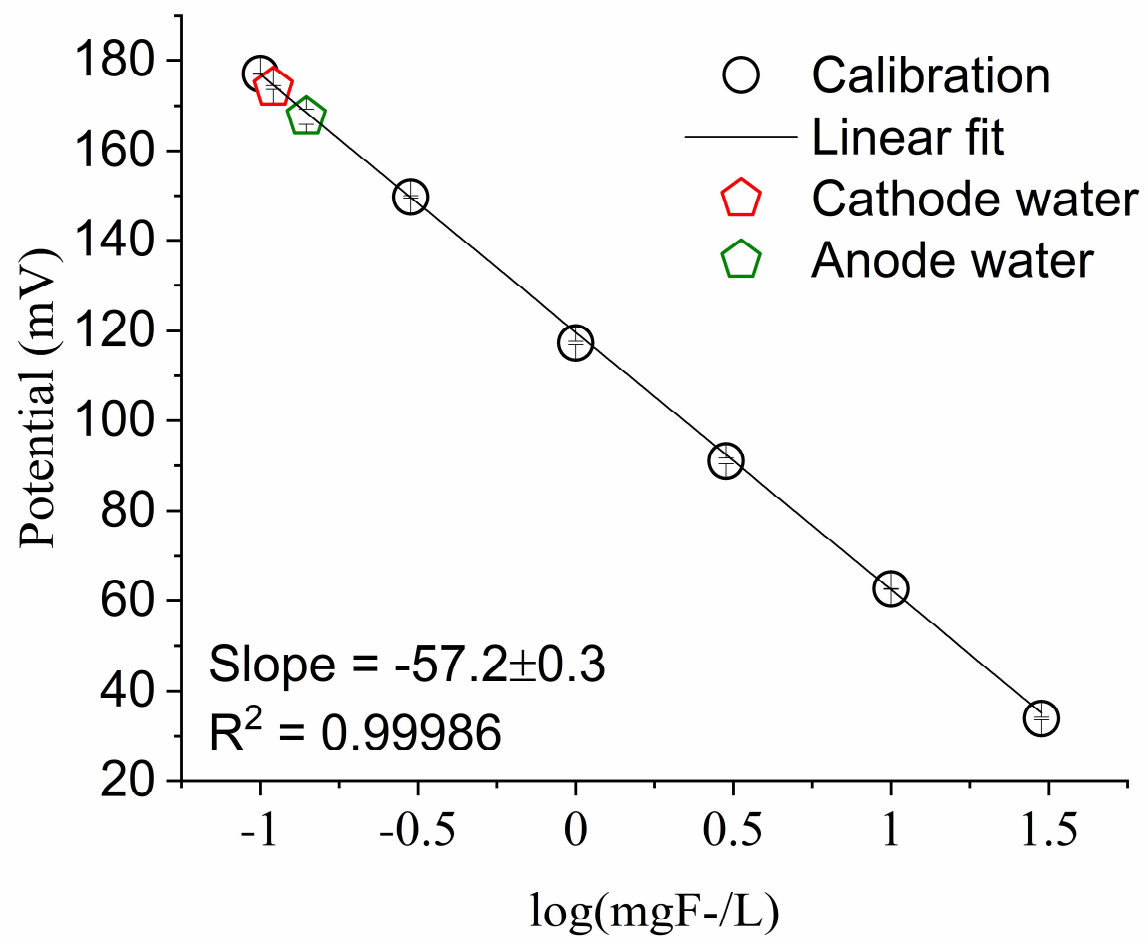

Fig. S32. Fluoride ion-selective electrode calibration curve and measured fluoride concentrations in anode and cathode water samples after electrolysis.

The circulating anode reservoir initially contained 0.2 L of water. After the test, 34 mL of water remained in the anode reservoir, while 130 mL was collected from the cathode outlet. Fluoride concentrations determined from 10 mL aliquots were 0.14 ± 0.01 mg F^- L^{-1} and 0.11 ± 0.01 mg F^- L^{-1} for the anode and cathode water samples, respectively. Recalculation of the total volume of collected water yielded total fluoride concentrations of 4.8 ± 0.3 µg F^- on the anode side and 14.3 ± 1.3 µg F^- on the cathode side. The presence of small amounts of fluoride in both the anode and cathode water samples indicates the release of fluorine-containing degradation products during operation.

S15. Contact angle measurements

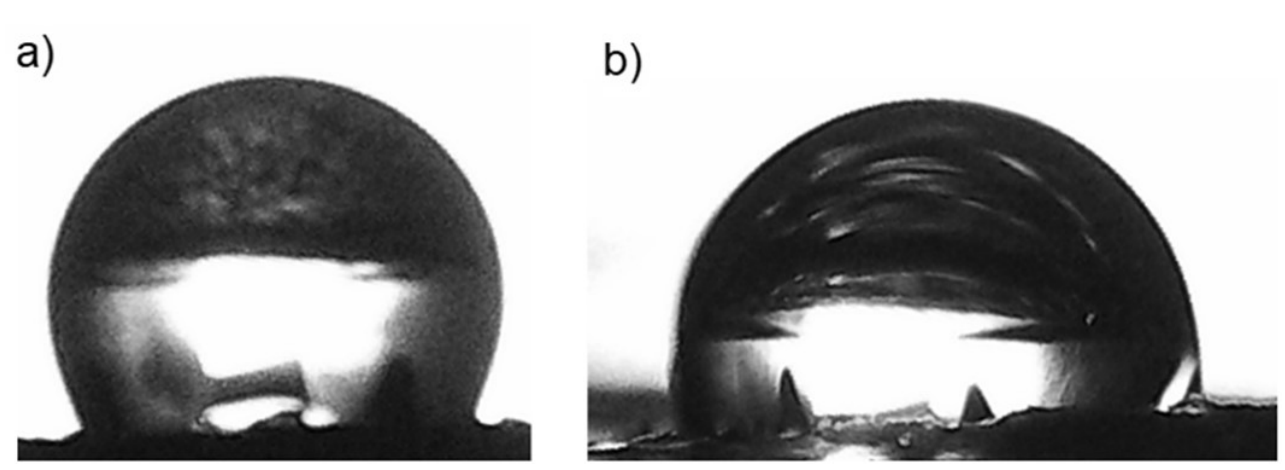

Fig. S33. Contact angle of IrTiN before (a) and after AST 0-2V (b).

S16. XPS after RDE

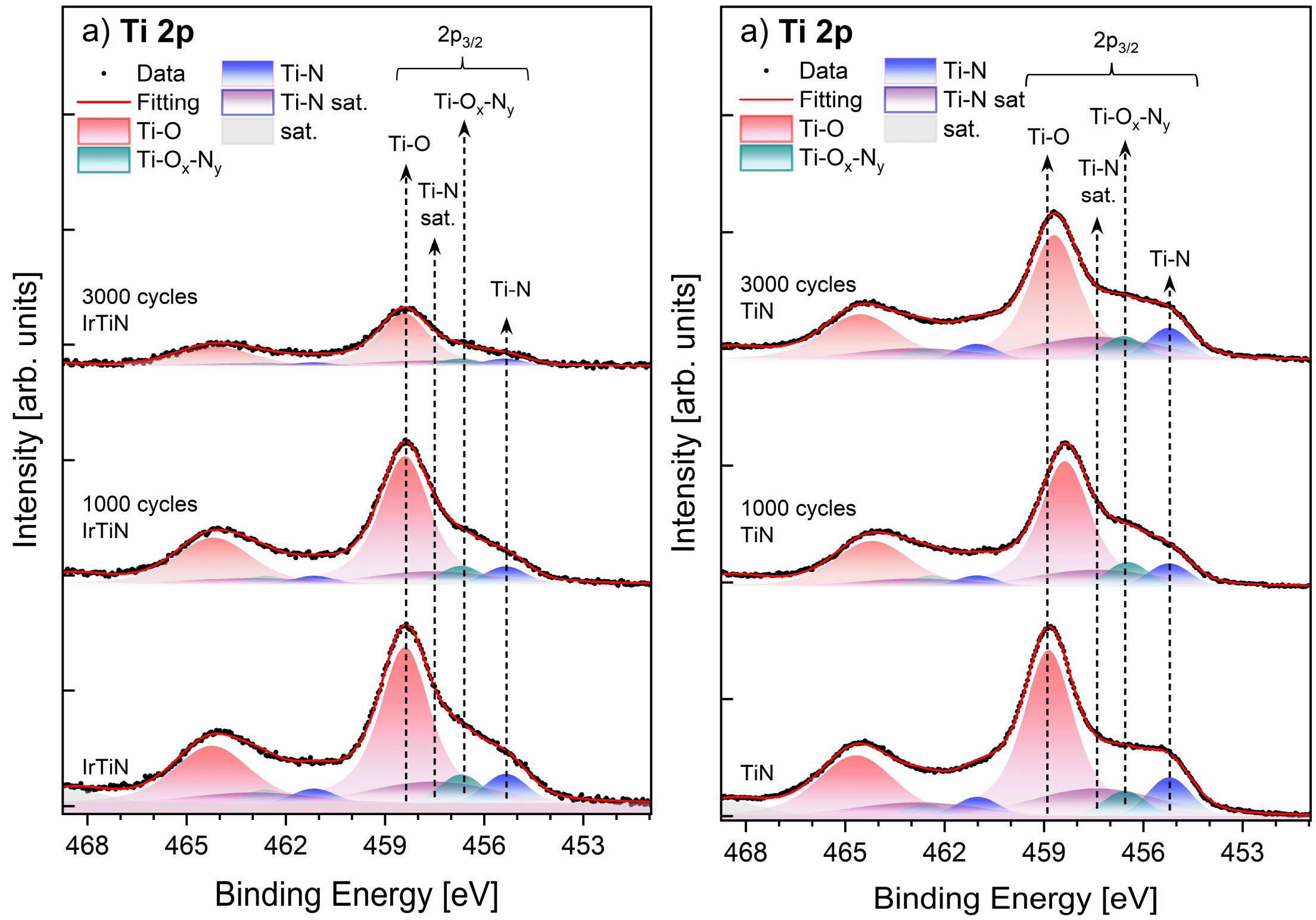

Fig. S34. Ti 2p XPS spectra of the TiN and IrTiN catalyst (without Nafion ionomer) deposited on a gold disk and subjected to the AST 0–2 V protocol in 0.1 M $HClO_4$ using an RDE setup. Spectra were collected before degradation and after 1000 and 3000 cycles.

S17. DRT analysis of PEIS data

Laboratory measurements: 0–2 V AST protocol

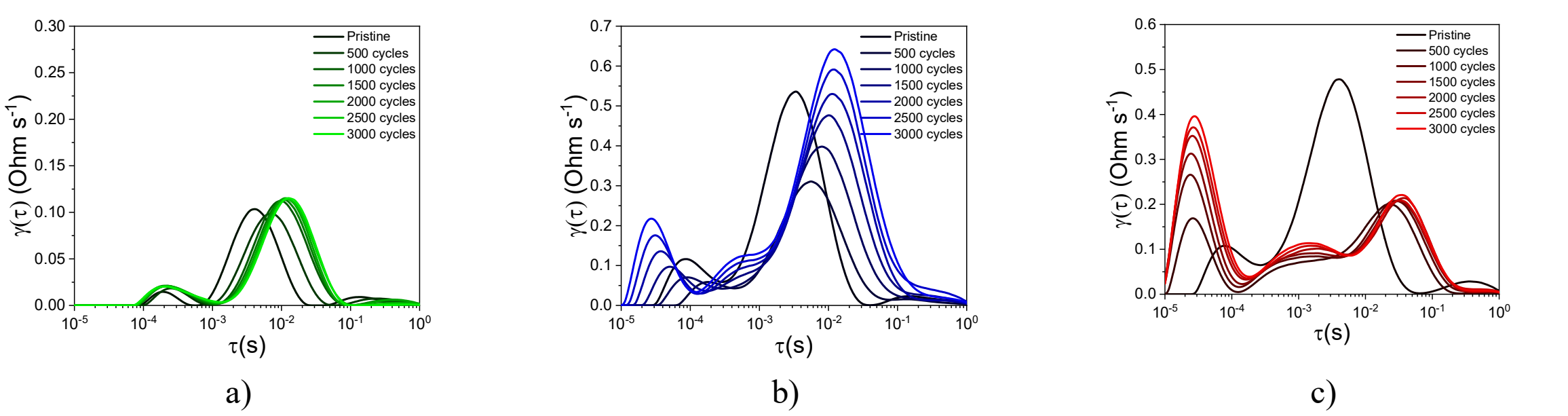

Fig. S35. DRT spectra derived from PEIS measurements at 1.5 V for water electrolysis cells with Ir/TiO_2, Ir/TiN, and Ir/TiC anodes, subjected to the 0–2 V AST protocol. Regularization parameter λ=0.1.

Laboratory measurements: 0–1.7 V AST protocol

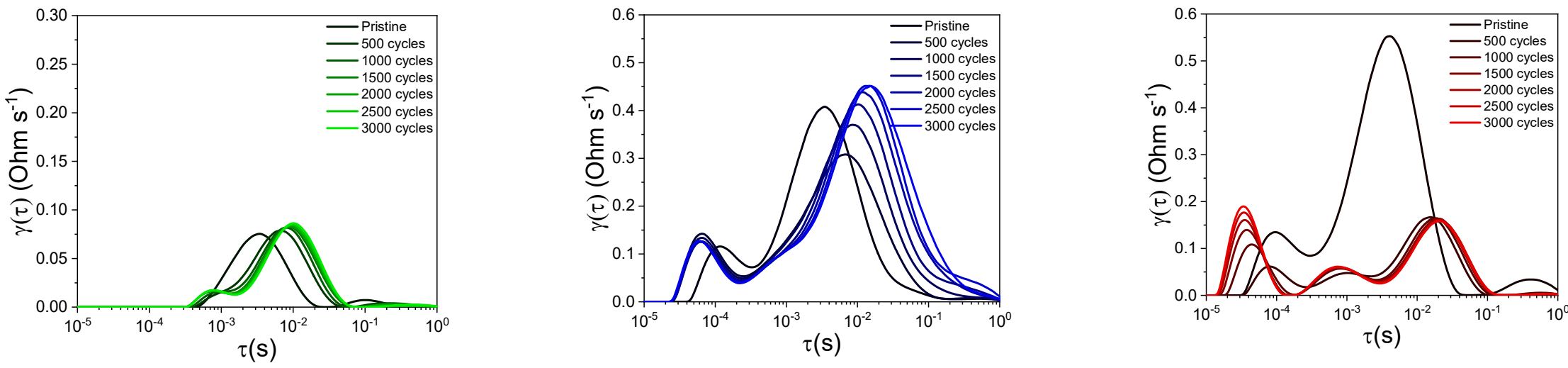

a) b) c)

Fig. S36. DRT spectra derived from PEIS measurements at 1.5 V for water electrolysis cells with Ir/TiO_2, Ir/TiN, and Ir/TiC anodes, subjected to the 0–1.7 V AST protocol. Regularization parameter λ=0.1.

The overall trends observed in the PEIS spectra and the corresponding DRT curves obtained during the operando (**Fig. S18**) and laboratory (**Fig. S35**) measurements are in good agreement. Similar behavior was also observed under the milder 0–1.7 V AST protocol (**Fig. S36**). It should be noted that the operando PEIS measurements were performed with only 6 points per decade due to limited synchrotron beamtime, whereas the in-house measurements were carried out with 25 points per decade, significantly improving the reliability and resolution of the DRT analysis. The DRT spectra reveal two main relaxation processes: a slow relaxation process at approximately 10^{-2} s associated with charge-transfer phenomena and a faster process below 10^{-3} s attributed to ion transport within the catalyst layer. In addition, the Ir/TiN and Ir/TiC samples exhibit the appearance and progressive increase in intensity of an additional intermediate relaxation peak during cycling. This feature is tentatively attributed to changes in ionic transport within the catalyst layer caused by Ti migration and ionomer contamination, in agreement with the EDX and post-mortem characterization results.

The DRT spectra after degradation show clear changes with applied potential (**Fig. S37-38**). At higher cell voltages, where the electrochemical reaction becomes more intensive, the relaxation peak shifts toward shorter relaxation times and decreases in intensity. This trend is consistent with the previous interpretation of the DRT evolution and further supports the relation of these peaks to ion transport processes in the catalyst layer.

Laboratory measurements: 0–2 V AST protocol

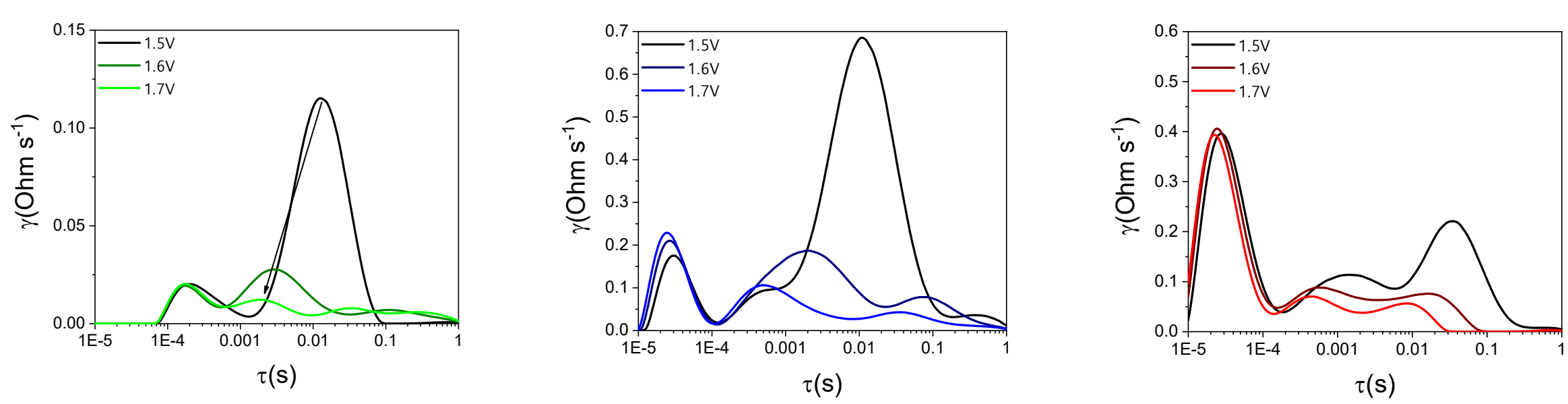

a) b) c)

Fig. S37. DRT spectra derived from PEIS data measured at 1.5, 1.6, and 1.7 V for water electrolysis cells with Ir/TiO_2, Ir/TiN, and Ir/TiC anodes, subjected to the 0–2 V AST protocol. The data shown correspond to measurements performed after 3000 cycles. Regularization parameter λ=0.1.

Laboratory measurements: 0–1.7 V AST protocol

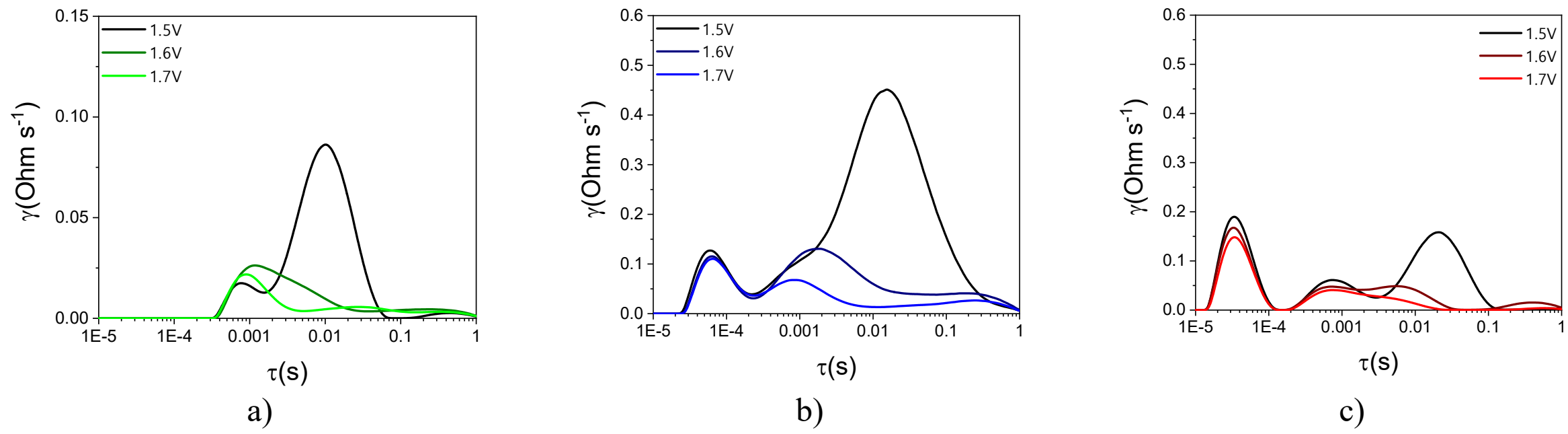

Fig. S38. DRT spectra derived from PEIS data measured at 1.5, 1.6, and 1.7 V for water electrolysis cells with Ir/TiO_2, Ir/TiN, and Ir/TiC anodes, subjected to the 0–1.7 V AST protocol. The data shown correspond to measurements performed after 3000 cycles. Regularization parameter λ=0.1.

S18. Lateral conductivity measurements using PEIS

Electrochemical impedance spectroscopy and lateral resistance measurements. PEIS measurements were performed using a BioLogic SP-300 potentiostat (BioLogic Science Instruments, France). The measurements were carried out on catalyst-coated membranes (CCMs) based on Nafion N212, prepared following the same procedure described in the manuscript. The lateral resistance of the samples was measured on the anode side of the CCMs using a four-point probe configuration. Impedance spectra were recorded at open-circuit potential using a 10 mV sinusoidal perturbation in the frequency range from 200 kHz to 100 mHz with 6 logarithmically spaced points per decade. The resistance was determined from the high-frequency intercept of the Nyquist plot with the real impedance axis. No normalization with respect to catalyst layer thickness was applied. For pristine samples, measurements were performed on three independently prepared CCMs, with three measurements recorded for each CCM.

Table S7. Resistance of the CCM before and after degradation tests in the water electrolyzer cell.

№	Samples	Resistance (Ohm)	№	Samples	Resistance (Ohm)
	Before AST			After AST	
1	TiO_2	5310±632	7	IrTiO_2-$AST_{0\text{-}2V}$	no stable value
2	TiN	4790±545	8	IrTiN-$AST_{0\text{-}2V}$	no stable value
3	TiC	51±6	9	IrTiC-$AST_{0\text{-}2V}$	no stable value
4	IrTiO_2 initial	93±14	10	IrTiO_2-$AST_{0\text{-}1.7V}$	no stable value
5	IrTiN initial	80±13	11	IrTiN-$AST_{0\text{-}1.7V}$	no stable value
6	IrTiC initial	77±18	12	IrTiC-$AST_{0\text{-}1.7V}$	637±304

Note: All samples were catalyst-coated membranes (CCMs) containing Nafion ionomer in the catalyst layer. "No stable value" indicates that a stable resistance response could not be obtained during the four-point probe measurement.

Measurements of degraded samples were generally not possible, most likely due to membrane swelling, which prevented stable electrical contact and may have disrupted the conductive network within the catalyst layer. The only exception was the Ir/TiC-$AST_{0–1.7\ V}$ sample, however, its reliability remains uncertain. Such ex situ measurements may not reflect the actual conductivity under electrolyzer operating conditions, where current is collected through the porous transport layer (PTL).

S19. Ionic conductivity measurements using PEIS

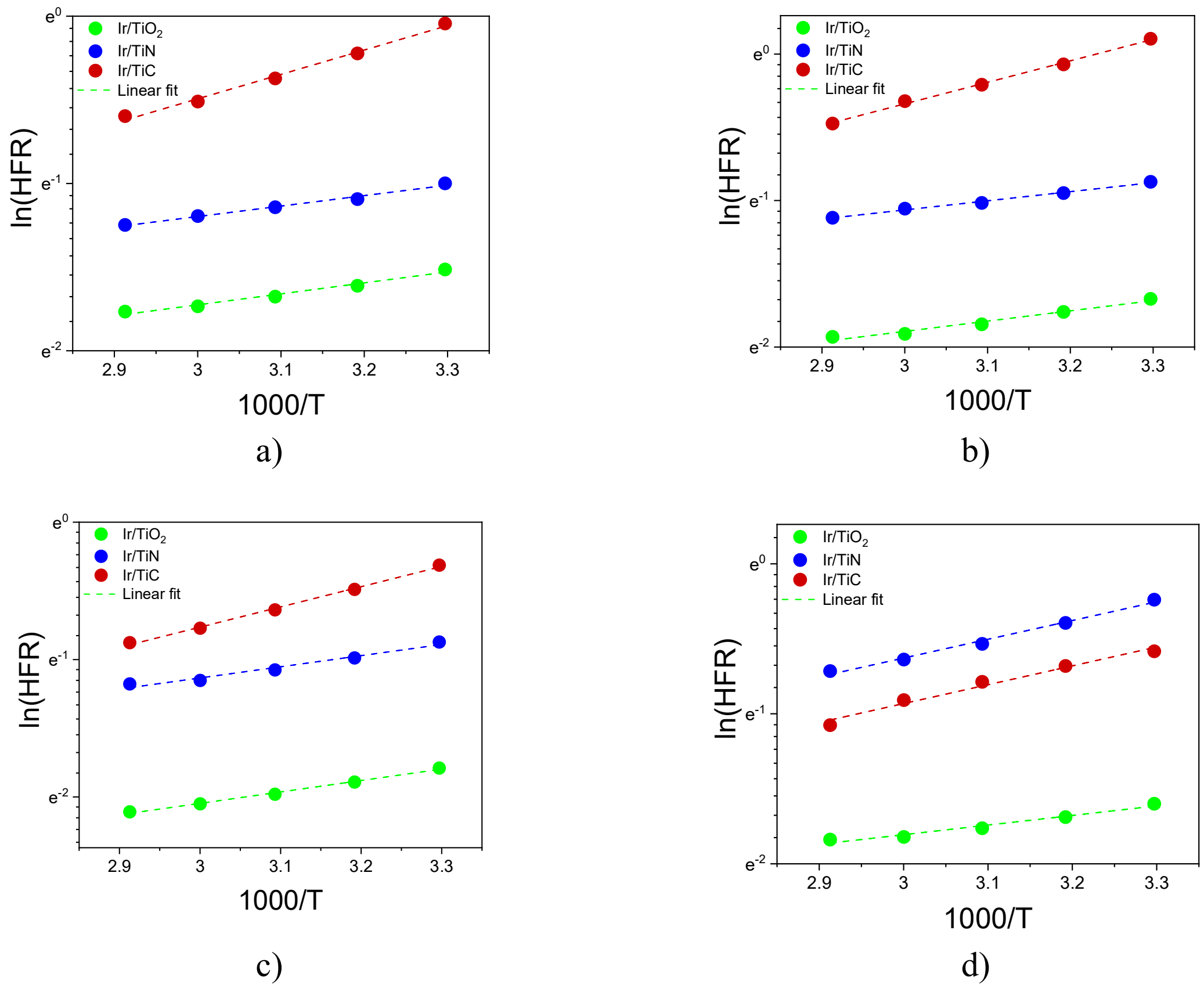

Fig. S39 Temperature dependencies of ln(HFR) for MEA incorporating Ir/TiO_2, Ir/TiN, and Ir/TiC passing (a,b) 0-2V, (c) 0-1.7V, and 0.4 A/cm^2 (for 25 hours) stress tests.

Table S8. Fitting parameters of the temperature dependency of HFR.

$AST_{0\text{-}2V}$-1

System/Parameter	Ir/TiO_2	Ir/TiN	Ir/TiC
Slope (Ea/R)	0.65675 ± 0.05757	0.62535 ± 0.03958	1.45814 ± 0.07116
Intercept	-3.69583 ± 0.17858	-3.07383 ± 0.12276	-4.86849 ± 0.22075
R-Square	0.97747	0.98813	0.99291
**Calculated Ea (kJ mol^{-1})**	**5.46±0.48**	**5.20±0.33**	**12.12±0.59**

$AST_{0\text{-}2V}$-2

System/Parameter	Ir/TiO_2	Ir/TiN	Ir/TiC
Slope (Ea/R)	0.70177 ± 0.0694	0.62329 ± 0.02935	1.46885 ± 0.04742
Intercept	-3.99846 ± 0.21528	-2.9338 ± 0.09104	-4.7464 ± 0.14708
R-Square	0.9715	0.99339	0.99688
**Calculated Ea (kJ mol^{-1})**	**5.83±0.58**	**5.18±0.24**	**12.21±0.39**

$AST_{0\text{-}1.7V}$

System/Parameter	Ir/TiO_2	Ir/TiN	Ir/TiC
Slope (Ea/R)	0.83189 ± 0.03653	0.81139 ± 0.08295	1.47236 ± 0.04956
Intercept	-4.54227 ± 0.11333	-3.56786 ± 0.25731	-5.18104 ± 0.15373
R-Square	0.99425	0.9696	0.99661
**Calculated Ea (kJ mol^{-1})**	**6.91±0.30**	**6.74±0.67**	**12.23±0.41**

At 0.4 Acm^{-2} constant current test for 25 hours.

System/Parameter	Ir/TiO_2	Ir/TiN	Ir/TiC
Slope (Ea/R)	0.64093 ± 0.06983	1.25039 ± 0.07205	1.25667 ± 0.10699
Intercept	-3.72866 ± 0.21661	-4.37952 ± 0.22349	-4.70183 ± 0.3319
R-Square	0.96561	0.99014	0.97872
**Calculated Ea (kJ mol^{-1})**	**5.33±0.58**	**10.39±0.60**	**10.44±0.89**

High-frequency resistances (HFRs) of MEAs incorporating Ir/TiO_2, Ir/TiN, and Ir/TiC anodes were measured after accelerated stress testing using 0–2.0 V and 0–1.7 V protocols. In all cases, ln(HFR) varied linearly with reciprocal temperature, indicating Arrhenius-like behavior of the measured ohmic resistance:

$$R_{HFR}(T) = R_0 \exp\left(\frac{Ea}{RT}\right),$$

where R= 8.31 J mol^{-1}K^{-1} – a gas constant, Ea – the apparent activation energy associated with the temperature dependence of HFR, T – temperature.

After the stress tests, the absolute HFR values followed the trend Ir/TiO_2 < Ir/TiN < Ir/TiC, indicating different extents and/or modes of degradation in the corresponding MEAs. Repetition of the 0–2.0 V protocol gave similar results, supporting the reproducibility of the measurements. In the discussion below, we assume that the temperature dependence of the measured HFR is governed predominantly by the ion-conducting phase of the MEA, i.e., mainly the membrane, while recognizing that contributions from the catalyst layer, interfaces, and other series resistances may also be present. Although the Ir/TiO_2 and Ir/TiN -based MEAs exhibited different absolute HFR values, the corresponding apparent activation energies were similar. The apparent activation energies obtained for the TiO_2 and TiN-based systems after stress testing were in the range of ca. 5.2–5.8 kJ mol^{-1}, which corresponds to the value 5.79 kJ mol^{-1} reported in [27,28] and somewhat lower reported values of ~8 kJ mol^{-1} [28]. This difference may indicate that the measured HFR contains not only the thermally activated ionic resistance of the membrane/ionomer phase, but also an additional temperature-independent or weakly temperature-dependent contribution. Such a contribution could arise from passivation of conductive components, interfacial degradation, or structural changes in the catalyst layer. Within this framework, the similar Ea values observed for the TiO_2 and TiN-based systems, despite different absolute HFR values, suggest that the higher HFR in the TiN case may be associated, at least in part, with additional series resistance that is only weakly temperature dependent.

By contrast, the TiC-based system showed both the highest HFR and a substantially larger apparent activation energy (∼12.2 kJ mol^{-1}). This result is consistent with a stronger perturbation of the ion-conducting phase in the TiC-based MEA. One possible interpretation is that the more pronounced degradation of the TiC support gives rise to Ti-containing species that alter the ionic transport properties of the membrane and/or ionomer. Study [28] showed that membrane contamination by foreign ions leads to increase in Ea, so a similar effect may therefore be expected for Ti ions presence. However, because the measured HFR reflects the sum of multiple resistance contributions, the extracted Ea should be regarded as an apparent parameter of the overall ohmic pathway rather than a direct measure of membrane proton transport alone.

Under the 0–1.7 V stress protocol, all systems exhibited slightly lower HFR values than after the 0–2.0 V protocol, consistent with less extensive degradation. For the TiO_2 and TiN-based systems, the apparent activation energies increased and approached values more typical of Nafion-based ion-conducting media. This trend is consistent with a decreased contribution from temperature-independent resistance under milder stress conditions. In the TiC-based system, the apparent Ea remained similar to that obtained after the 0–2.0 V protocol. Although this similarity may indicate persistent modification of the ion-conducting phase, deconvolution of intrinsic membrane/ionomer transport from other resistive contributions is not possible from HFR data alone.

References

[1] Bogar M, Yakovlev Y, Novakova J, Darabut AM, Kriechbaum M, Amenitsch H, et al. A small angle X-ray scattering approach for investigating fuel cell catalyst degradation for both *ex situ* and *in operando* analyses. Int J Hydrog ENERGY 2024;58:1673–81. https://doi.org/10.1016/j.ijhydene.2024.01.261.

[2] Bogar M, Yakovlev Y, Pollastri S, Biagi R, Amenitsch H, Taccani R, et al. Capabilities of a novel electrochemical cell for operando XAS and SAXS investigations for PEM fuel cells and water electrolysers. J Power Sources 2024;615:235070. https://doi.org/10.1016/j.jpowsour.2024.235070.

[3] Humlíček J. Optimized computation of the Voigt and complex probability functions. J Quant Spectrosc Radiat Transf 1982;27:437–44. https://doi.org/10.1016/0022-4073(82)90078-4.

[4] Armstrong BH. Spectrum line profiles: the Voigt function. J Quant Spectrosc Radiat Transf 1967;7:61–88.

[5] Whiting EE. An empirical approximation to the Voigt profile. J Quant Spectrosc Radiat Transf 1968;8:1379–84. https://doi.org/10.1016/0022-4073(68)90081-2.

[6] Drayson SR. Rapid computation of the Voigt profile. J Quant Spectrosc Radiat Transf 1976;16:611–4. https://doi.org/10.1016/0022-4073(76)90029-7.

[7] Schreier F. The Voigt and complex error function: A comparison of computational methods. J Quant Spectrosc Radiat Transf 1992;48:743–62. https://doi.org/10.1016/0022-4073(92)90139-U.

[8] Yarusso DJ, Cooper SL. Microstructure of ionomers: interpretation of small-angle x-ray scattering data. Macromolecules 1983;16:1871–80. https://doi.org/10.1021/ma00246a013.

[9] Gebel G. Structural evolution of water swollen perfluorosulfonated ionomers from dry membrane to solution. Polymer 2000;41:5829–38. https://doi.org/10.1016/S0032-3861(99)00770-3.

[10] Gommes CJ, Prieto G, De Jongh PE. Small-Angle Scattering Analysis of Empty or Loaded Hierarchical Porous Materials. J Phys Chem C 2016;120:1488–506. https://doi.org/10.1021/acs.jpcc.5b09556.

[11] Asset T, Gommes CJ, Drnec J, Bordet P, Chattot R, Martens I, et al. Disentangling the Degradation Pathways of Highly Defective PtNi/C Nanostructures – An Operando Wide and Small Angle X-ray Scattering Study. ACS Catal 2019;9:160–7. https://doi.org/10.1021/acscatal.8b02665.

[12] Gommes CJ, Asset T, Drnec J. Small-angle scattering by supported nanoparticles: exact results and useful approximations. J Appl Crystallogr 2019;52:507–19. https://doi.org/10.1107/S1600576719003935.

[13] Sharma RV, Sharma KC. The structure factor and the transport properties of dense fluids having molecules with square well potential, a possible generalization. Phys Stat Mech Its Appl 1977;89:213–8. https://doi.org/10.1016/0378-4371(77)90151-0.

[14] Pontoni D, Finet S, Narayanan T, Rennie AR. Interactions and kinetic arrest in an adhesive hard-sphere colloidal system. J Chem Phys 2003;119:6157–65. https://doi.org/10.1063/1.1601605.

[15] Bogar M, Yakovlev Y, Sandbeck DJS, Cherevko S, Matolínová I, Amenitsch H, et al. Interplay Among Dealloying, Ostwald Ripening, and Coalescence in $Pt_X Ni_{100-X}$ Bimetallic Alloys under Fuel-Cell-Related Conditions. ACS Catal 2021;11:11360–70. https://doi.org/10.1021/acscatal.1c01111.

[16] Kregar A, Kravos A, Katrašnik T. Methodology for Evaluation of Contributions of Ostwald Ripening and Particle Agglomeration to Growth of Catalyst Particles in PEM Fuel Cells. Fuel Cells 2020;20:487–98. https://doi.org/10.1002/fuce.201900208.

[17] Kregar A, Katrašnik T. Theoretical analysis of particle size re-distribution due to Ostwald ripening in the fuel cell catalyst layer. Open Phys 2019;17:779–89. https://doi.org/10.1515/phys-2019-0081.

[18] Thamaphat K, Limsuwan P, Ngotawornchai B. Phase Characterization of TiO_2 Powder by XRD and TEM n.d. Kasetsart Journal (Natural Science). 2008, 42:357–361.

[19] Sigwadi R, Dhlamini MS, Mokrani T, Ṋemavhola F, Nonjola PF, Msomi PF. The proton conductivity and mechanical properties of Nafion®/ ZrP nanocomposite membrane. Heliyon 2019;5:e02240. https://doi.org/10.1016/j.heliyon.2019.e02240.

[20] Yang JH, Kim KH, Bark CW, Choi HW. The effect of dye-sensitized solar cell based on the composite layer by anodic TiO_2 nanotubes. Nanoscale Res Lett 2014;9:671. https://doi.org/10.1186/1556-276X-9-671.

[21] Si Q, Matsui M, Horiba T, Yamamoto O, Takeda Y, Seki N, et al. Carbon paper substrate for silicon–carbon composite anodes in lithium-ion batteries. J Power Sources 2013;241:744–50. https://doi.org/10.1016/j.jpowsour.2013.05.090.

[22] Gurgenc T, Macit CK, Biryan F, Gurgenc E, Ozel C, Bellucci S. Investigation of dielectric parameters and AC conductivity of ZnO: TiC nanocomposite powders. Appl Phys A 2023;129:649. https://doi.org/10.1007/s00339-023-06932-3.

[23] Cui Y, Li C, Li R, Wei Y, Lv D, Bu J, et al. Effect of synthesis temperatures on the composition, microstructure, and microwave absorption properties of titanium nitride porous nanofibers prepared using ammonia reduction nitridation process. J Mater Sci Mater Electron 2023;34:1036. https://doi.org/10.1007/s10854-023-10471-1.

[24] Alderman OLG, Benmore CJ, Tamalonis A, Sendelbach S, Heald S, Weber R. Continuous Structural Transition in Glass-Forming Molten Titanate $BaTi_2O_5$. J Phys Chem C 2016; 120, 26974–85. https://doi.org/10.1021/acs.jpcc.6b08248.

[25] Masai H, Sakurai H, Koreeda A, Fujii Y, Ohkubo T, Miyazaki T, et al. Photocatalytic hydrogen generation of monolithic porous titanium oxide-based glass–ceramics. Sci Rep 2020;10:11615. https://doi.org/10.1038/s41598-020-68410-7.

[26] U.S. Environmental Protection Agency (EPA). Method 9214: Potentiometric determination of fluoride in aqueous samples with selective electrode, 1996.

[27] Matos BR, Goulart CA, Santiago EI, Muccillo R, Fonseca FC. Proton conductivity of perfluorosulfonate ionomers at high temperature and high relative humidity. Appl Phys Lett 2014;104:091904. https://doi.org/10.1063/1.4867351.

[28] Hongsirikarn K, Goodwin JG, Greenway S, Creager S. Effect of cations (Na^+, Ca^{2+}, Fe^{3+}) on the conductivity of a Nafion membrane. J Power Sources 2010;195:7213–20. https://doi.org/10.1016/j.jpowsour.2010.05.005.
